# Biocatalytic Conversion of Furans into Pyrrolinones Using a Class I Unspecific Peroxygenase

**DOI:** 10.1021/acscatal.5c05307

**Published:** 2025-09-04

**Authors:** Benjamin Melling, Katy A. S. Cornish, Jared Cartwright, Nicholas P. Mulholland, William P. Unsworth, Gideon Grogan

**Affiliations:** † Department of Chemistry, 8748 University of York, Heslington, York YO10 5DD, U.K.; ‡ Syngenta, Jealott’s Hill International Research Centre, Bracknell, Berkshire RG42 6EY, UK; § Department of Biology, 8748 University of York, Heslington, York YO10 5DD U.K.

**Keywords:** biocatalysis, peroxygenase, UPO, furan, 4-pyrrolin-2-one

## Abstract

The biocatalytic conversion of furans and amines into 4-pyrrolin-2-ones can be achieved using the Class I Unspecific Peroxygenase artUPO. The reactions are broad in scope and typically operate in good yield, using a simple, easily scalable H_2_O_2_ driven protocol, in which exogenous enzyme cofactors are not required. All of these biotransformations were performed on a preparative scale to deliver synthetically useful quantities of pyrrolinones. A cascade mechanism likely operates, in which UPO-mediated oxidative ring opening of the furan first delivers a reactive 1,4-dicarbonyl intermediate, followed by condensation with the added amine and rearrangement to the pyrrolinone product outside the UPO active site.

## Graphical abstract

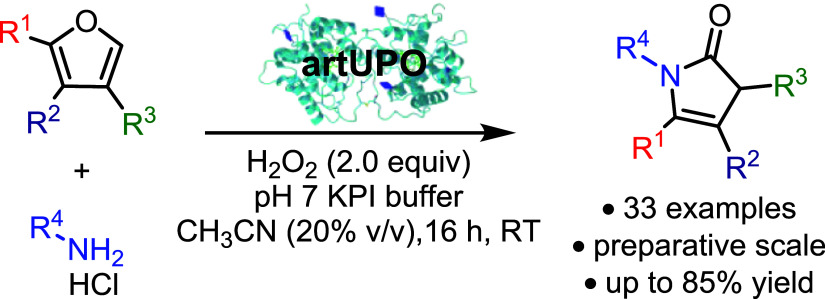

## Introduction

Unspecific peroxygenases (UPOs) are heme oxygenases that catalyze the oxygenation of organic substrates at the expense only of exogenous hydrogen peroxide (H_2_O_2_).^1–4^ As they are easy to prepare on a large scale^5^ and inexpensive to apply, they are now becoming established as biocatalysts with significant potential for scalable applications in synthesis. In addition to small-scale demonstrations of their application in the oxygenation of simple hydrocarbons,^6^ fatty acids,^7,8^ aromatics^9,10^ and pharmaceuticals^11^ UPOs have now been applied at scale in the oxygenation of butane^12^ and cyclohexane.^13^ In addition, the emerging availability of a diversity of natural UPOs^14–17^ and mutants^18,19^ means that a range of selectivities can be accessed, suitable for diverse reaction outcomes.

UPOs can be broadly divided into two classes based primarily on their molecular weight, with Class I and Class II UPOs having masses of around 26 kDa and 44 kDa, respectively.^14,20^ A significant focus of our previous work on preparative UPO oxygenation reactions has been the comparison of the reactions catalyzed by representative Class I and Class II UPOs. In particular, we have focused on the development of novel oxygenation reactions catalyzed by the Class I UPO, artUPO and the Class II UPO from *Agrocybe aegerita* (r*Aae*UPO-PaDa-I-H). In selected cases, these two UPOs are able to catalyze remarkably divergent transformations of the same substrates; for example, r*Aae*UPO-PaDa-I-H and artUPO promote the stereodivergent oxygenation of sulfides of type **1** to form (*R*)- and (*S*)-sulfoxides respectively (Scheme 1A).^21^ The same UPOs can also promote divergent reaction outcomes with respect to chemoselectivity; for example, when challenged with 3-carene **3**, r*Aae*UPO-PaDa-I-H afforded carboxylic acid **4a** as its major oxygenation product via methyl group oxygenation, whereas artUPO converted the same substrate **3** into epoxide **4b** and enone **4c**, via alkene epoxidation and allylic methylene oxidation.^22^

**Scheme 1:**
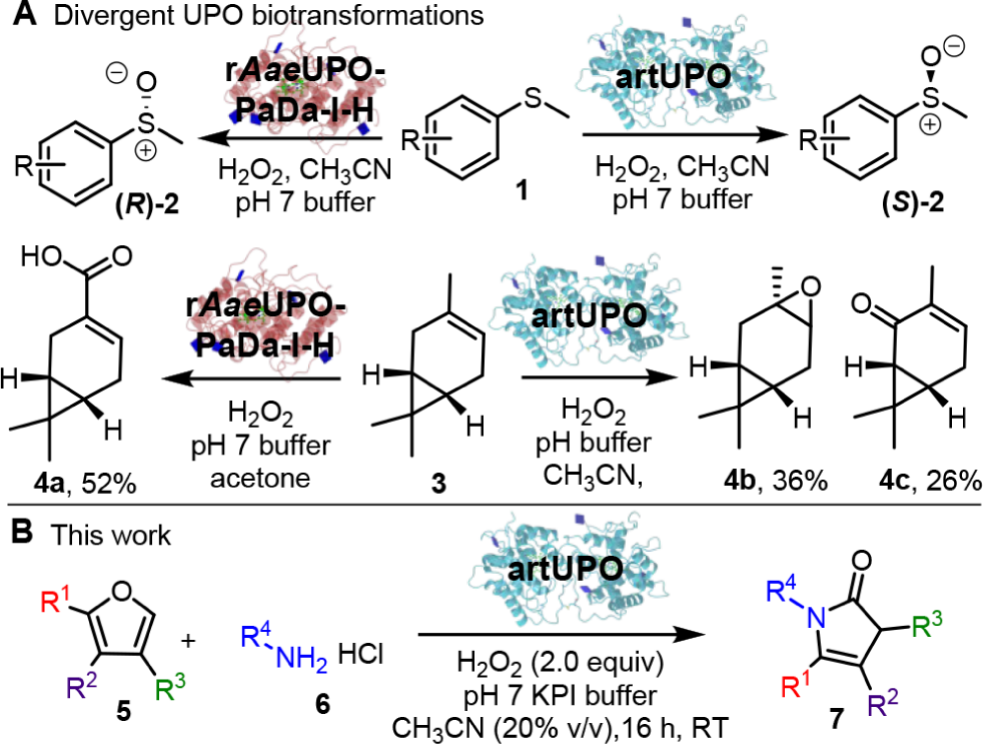
A: Divergent UPO Reactions (Previous Work) and B: Biocatalytic Conversion of Furans into 4-Pyrrolin-2-ones using artUPO (This Work)

In common with cytochromes P450 (P450s), the other large family of heme oxygenases that has been intensively investigated for applications in synthesis,^23–25^ UPOs also display “promiscuous” activity for transformations other than simple hydroxylations. The epoxidation of certain furan substrates, for example, can lead to Achmatowicz-type rearrangements to give dihydropyrans.^26^ At low pHs and in the presence of halide ions, UPOs can form hypohalous acids that enable halogenation reactions.^27^ In addition UPOs have been shown to catalyze the oxidation of silanes^28^ and phenol coupling reactions.^29^ More recently we have also shown that mutants of artUPO catalyze asymmetric cyclopropanation reactions^30^ in the mode of both P450s^31^ and myoglobin (Mb) mutants.^32^

This report is focused on the discovery of a new promiscuous UPO reaction: the biocatalytic conversion of furans **5** and amines **6** into 4-pyrrolin-2-ones **7** by the Class I artUPO. This represents a new-to-biocatalysis transformation, discovered by serendipity as part of our efforts to further explore the divergent reactivity of Class I and II UPOs. In total, 33 novel biotransformations to form pyrrolinones are reported. All biotransformations were performed on preparative scale, and delivered pure products in typically good yield, using a simple, scalable protocol.

## Results and Discussion

The new reactivity described in this report originated during studies to explore the preparative scale UPO-catalyzed oxygenation of fenfuram **5a**. Fenfuram is a fungicide used in seed-treatment, and studying its metabolites was of interest to our coworkers in the agrochemical industry. First, fenfuram **5a** was treated with r*Aae*UPO-PaDa-I-H and H_2_O_2_ as the stoichiometric oxidant, in an aqueous pH 7.0 buffer with acetonitrile as organic cosolvent at RT. The major product in this biotransformation was phenol **8**, which was isolated in 31% yield. Oxygenation of aromatics is a relatively common UPO transformation,^9,10^ hence the formation of this product was not a surprise. However, the analogous reaction using artUPO, under broadly the same conditions, gave an altogether more unexpected result. In this reaction, the major product formed was pyrrolinone **7a**, which was isolated in 35% yield (Scheme 2A).

**Scheme 2:**
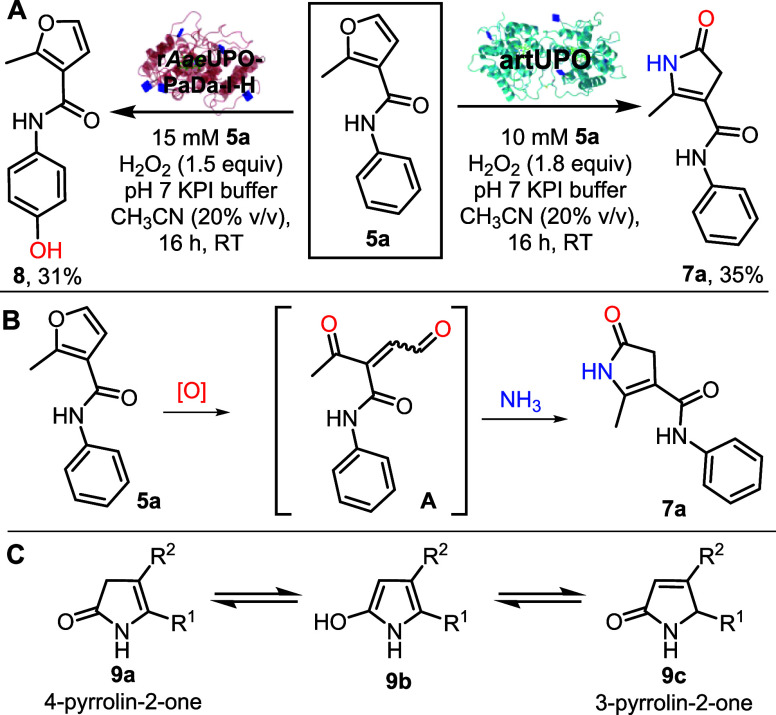
Divergent UPO Reactions of Fenfuram and Pyrrolinone Formation: A) Divergent UPO Reactions of Fenfuram 5a; B) General Pyrrolinone Formation; C) Pyrrolinone Tautomers

The most surprising aspect of this reaction was the incorporation of nitrogen into the product, given that there was no immediately obvious nitrogen source in the reaction mixture. However, it is notable that neither of the UPOs used in this study are applied as pure enzymes. In the case of artUPO, it is added to the biotransformation as a fermentation secretate (see our previous study for a description of its expression)^21^ including residual species used during its production in *Pichia pastoris*. Most pertinent to the formation of **7a** is that ammonium hydroxide was used as a base to regulate the pH during the fermentation. Given this, it is reasonable that the nitrogen in **7a** derived from adventitious ammonia in the artUPO secretate, enabling a reaction of the type summarized in Scheme 2B. It is well-known that furans can undergo ring-opening to form 1,4-dicarbonyls (or equivalents, of the form **A**) under oxidative conditions,^26^ and in the presence of ammonia, such an intermediate may be trapped and converted into **7a** *via in situ* condensation reactions.

4-Pyrrolin-2-ones (also known as 2-pyrrolin-5-ones) are able to exist in one of three tautomeric forms (Scheme 2C). For many substitution patterns, the 3-pyrrolin-2-one form **9c** is the most abundant tautomer.^33^ However, for pyrrolinones substituted with electron withdrawing groups in their 4-position (such as **7a**), the 4-pyrrolin-2-one tautomeric form **9a** is typically favored. 4-Pyrrolin-2-ones are common motifs in natural products and biologically active compounds,^34^ and they can also serve as versatile precursors to a range of other products, including more complex alkaloid scaffolds.^33^ Nonenzymatic synthetic methods to prepare 4-pyrrolin-2-ones have been reported, including methods based on condensation reactions,^34–36^ oxidation,^37,38^ and others.^39,40^

To the best of our knowledge, no biocatalytic methods for the preparation of 4-pyrrolin-2-ones had been reported prior to this study. We therefore set out to test whether our serendipitously discovered biocatalytic pyrrolinone formation could be applied more generally. First, we were keen to explore whether amines other than ammonia were compatible. Thus, fenfuram **5a** and ethylamine hydrochloride **6a** were incubated with artUPO and H_2_O_2_ (2 equiv). The biotransformation was performed using conditions similar to those used in previous UPO studies,^21,22,26,27,30^ in a pH 7 aqueous buffer with acetonitrile as cosolvent. H_2_O_2_ was added dropwise over 5 h *via* syringe pump, to minimize oxidative stress on the UPO (Scheme 3). Under these conditions, fenfuram **5a** and ethylamine hydrochloride **6a** were successfully converted into pyrrolinone **7b** in 45% yield on a preparative scale (100 mg of **7b** isolated). Notably the same ammonia-containing artUPO secretate tested in the fenfuram transformation was used, but none of ammonia adduct **7a** was observed. This suggests that the added ethylamine is able to outcompete adventitious ammonia in the reaction.

**Scheme 3:**
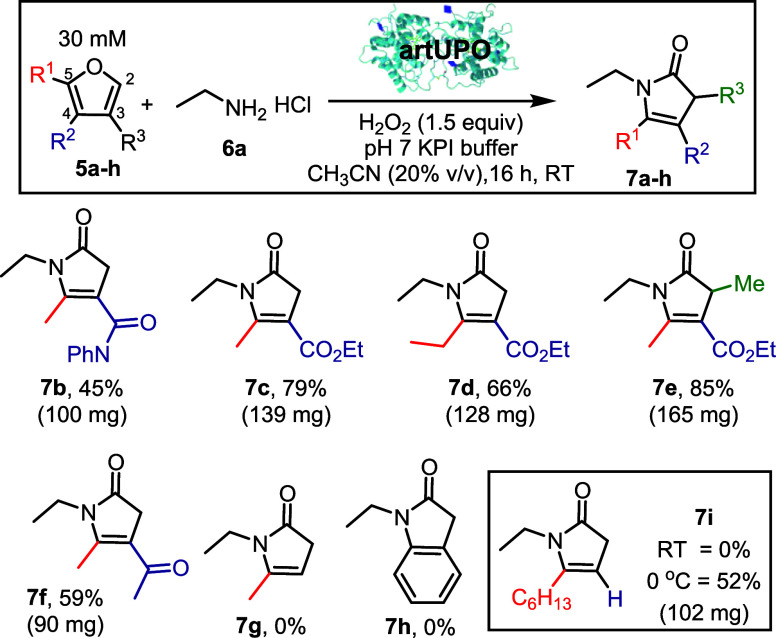
Conversion of Furans into Pyrrolinones using artUPO: Furan Scope^a^

a See Materials and Methods section, General Procedure 1, and Supporting Information for full synthetic etails.

The UPO mediated reactions of ethylamine hydrochloride **6a** with other substituted furans were tested next. The amide moiety of fenfuram could be replaced by an ethyl ester, with pyrrolinones **7c** and **7d** each obtained in higher yields (79% and 85%, both on <100 mg scale) than the analogous fenfuram transformations. Substitution of the furan 3-position is well tolerated, with pyrrolinone **7e** isolated in 85% yield using the standard procedure. Ketone substituted pyrrolinone **7f** was also obtained in good yield in the same way. The presence of an electron-withdrawing group at the furan 4-position (R^2^ in **5**) appears to be important in enabling an efficient reaction. For example, three furans without electron deficient 4-substituents were tested (to form **7g**–**7i**) using the standard conditions, and a complex mixture of products was obtained in each case. It is likely that the electron withdrawing group plays a key role in stabilizing the intermediates formed on route to the pyrrolinone, and helps prevent unwanted side reactions. However, by maintaining a lower reaction temperature (0 °C for 18 h) a successful biotransformation transformation of a furan lacking a 4-substituent was achieved, with 102 mg of pyrrolinone **7i** (52% yield) isolated using this modified procedure.

Next attention turned to exploring the scope of the reaction with respect to the amine (Scheme 4). We started by testing the biotransformation of furan **5b** and benzylamine. Initial results were disappointing; using the Scheme 3 conditions, the pyrrolinone product **7j** was obtained in just 9% isolated yield. The major product observed in this biotransformation was unreacted furan **5b**, with the low conversion thought to be a result of the amine interacting competitively with the heme in the UPO active site. Based on this, we considered whether slow addition of the amine may lead to improved conversion. Thus, an alternative protocol was established whereby both the amine and H_2_O_2_ are added slowly (over 5 and 8 h respectively) and this led to a much-improved result; using this method furan **5b** was fully consumed and the desired product isolated in 65% yield.

**Scheme 4:**
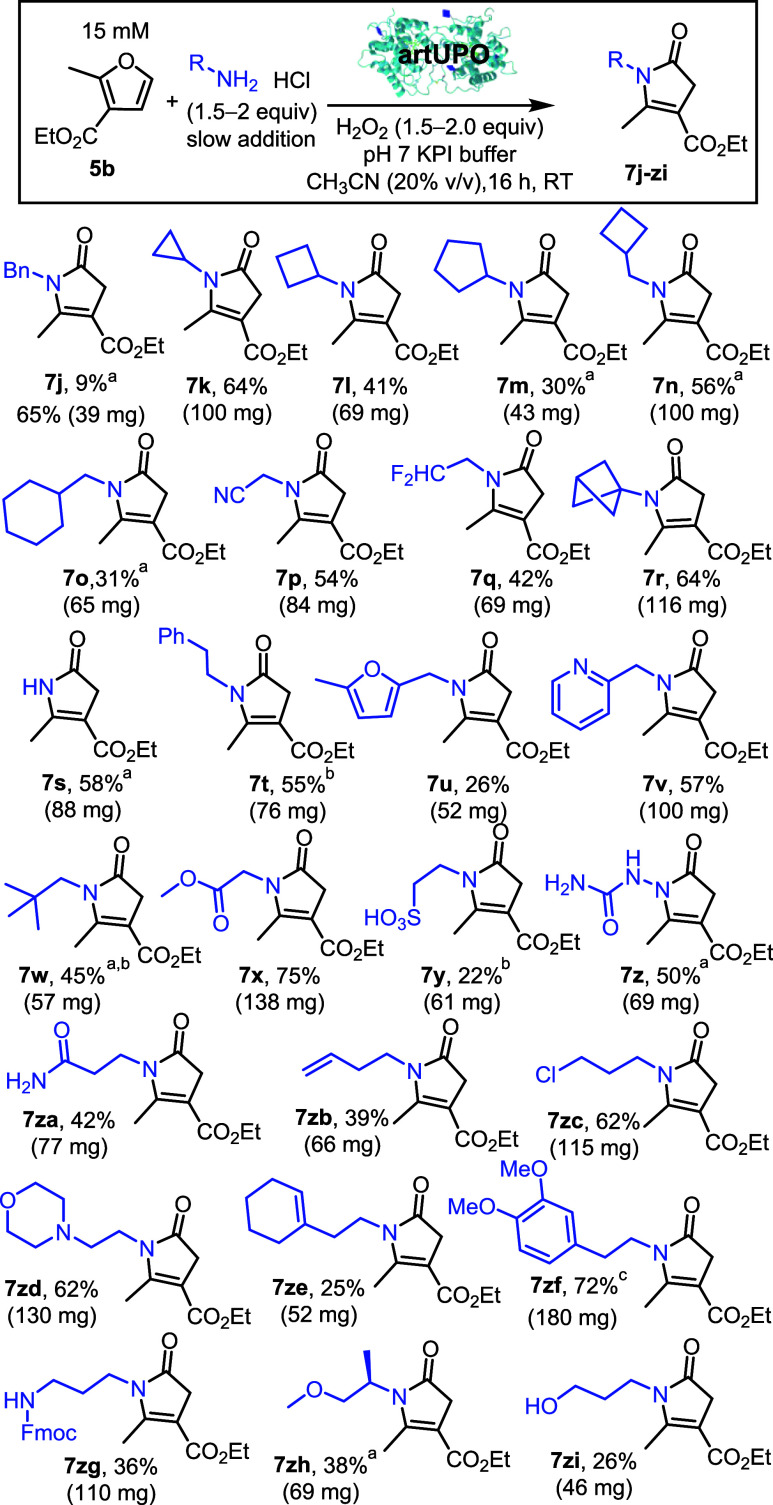
Conversion of Furans into Pyrrolinones using artUPO: amine Scope^a^ ^b^ ^c^ ^d^

a Amine added as a single portion; see Materials and Methods section, General Procedure 3, and Supporting Information for full synthetic details.
b The free amine rather than its HCl salt was used.
c Isolated as a 10:1 **7zf**:**7s** mixture.
d Unless stated, both the amine and H_2_O_2_ were added slowly *via* syringe pump, over 5 and 8 h respectively; see Materials and Methods section, General Procedure 2, and Supporting Information for full synthetic details.

This improved protocol was taken forward and used for the majority of the cases featured in Scheme 4 (see SI, Section 2.6 optimization of the amine addition method). All of these biotransformations were performed on preparative scale, and the yields refer to isolated products (39–180 mg) following column chromatography. Amines substituted with cyclic hydrocarbons were all compatible, either using the slow amine addition method, or direct amine addition at the start (**7k**–**7o**). More functionalized amines substituted with nitrile (**7p**), fluoroalkyl (**7q**), or bridged bicyclic groups (**7r**) also worked well using the standard protocol. Pyrrolinone **7s** was prepared using added ammonium chloride as the amine source, analogous to the serendipitous formation of **7a** described earlier. Amines substituted with aromatics and heteroaromatics (to form **7t**–**7v**) were also compatible; the successful formation of **7u** is notable despite its modest yield, given that the furan group on the amine survived the process without itself undergoing oxidation. Indeed, the functional group tolerance of the transformation is very high, with pyrrolinones successfully prepared from a wide range of functionalized amines, including sterically hindered groups (**7w**), esters (**7x**), sulfonic acids (**7y**), ureas (**7z**), amides (**7za**), alkenes (**7zb**), chloroalkanes (**7zc**), morpholine (**7zd**), cyclohexenes (**7ze**), anisoles (**7zf**), carbamate protected amines (**7zg**), ethers (**7zh**), and alcohols (**7zi**). This wide functional group compatibility attests to the generality of the oxidation and rearrangement cascade, and to the robustness of the UPO to tolerate a range of reactive groups.

A plausible mechanism for the overall reaction is proposed in Scheme 5. Control reactions confirm that artUPO is essential to the reaction, with no conversion observed when standard furan and amine substrates were reacted with H_2_O_2_ under the standard conditions in its absence. It is therefore likely that the reaction is initiated via furan oxidation via the heme Fe­(IV) oxo complex commonly known as “Compound I”, which is formed by reaction between artUPO and H_2_O_2_.^1–4^ UPO-mediated oxidative ring opening of a furan and subsequent rearrangement has been seen previously for the related peroxygenase r*Aae*UPO-PaDa–I-H in the context of a biocatalytic Achmatowicz reaction.^26^ It likely that a similar oxidative ring-opening (**5b → B → C** → **D**) operates in this case, to form an unsaturated keto-aldehyde of the form **D**, or a related water/amine adduct. Reaction of the aldehyde group of **D** with the amine can then initiate a cascade reaction to form the pyrrolinone; several related pathways can be envisaged here, broadly involving imine condensation (**D** → **E**), hydration and cyclization (**E** → **F**) and elimination/tautomerization (**F** → **7**). Nonenzymatic oxidative furan to pyrrolinone transformations are known,^33^ for example reactions driven by oxidative furan opening with singlet oxygen and subsequent amine trapping.^41^ Considering this, and noting the very broad amine compatibility, we think it is likely that the amine condensation steps take place spontaneously under the reaction conditions, outside the UPO active site.^42,43^

**Scheme 5:**
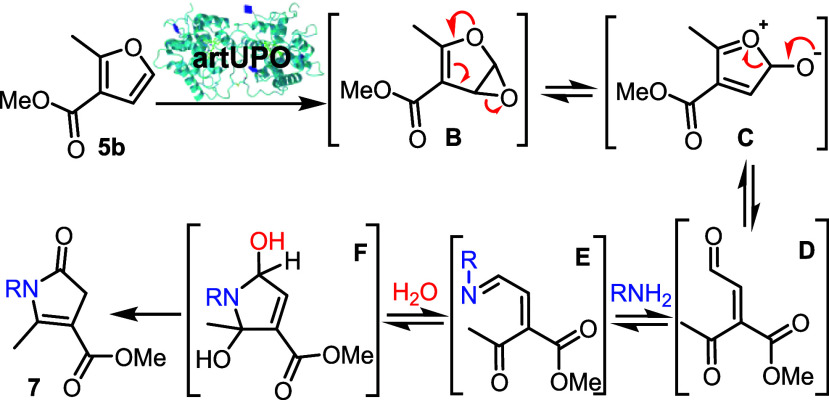
Proposed Mechanism

## Materials and Methods

### Enzyme Production

The cloning and expression of the artUPO used in this study and its preparation from fermentations of *Pichia pastoris* has been described previously.^21^ The enzyme was added to reactions in the form of the crude secretate from the *Pichia* fermentations.

#### General Procedure 1

The conversion of furans into pyrrolinones using artUPO, with amine added prior to hydrogen peroxide infusion (used for Scheme 3 results)

To a 100 mL round-bottom flask with containing a stirrer bar was added KPi buffer (24 mL, 100 mM, pH 7.00) and ethylamine hydrochloride salt (1.80 mmol, 146 mg, 2.00 equiv) at RT. After stirring for 10 min, artUPO secretate (1.00 mL) was added, followed by addition of a solution of the corresponding furan **5** (0.900 mmol) in MeCN (6.00 mL). The reaction was initiated by the slow addition (syringe pump addition) of an aqueous H_2_O_2_ solution (139 μL of a 30% H_2_O_2_ solution diluted up to 4.00 mL with water, 1.35 mmol, 0.75 mL/h) followed by overnight stirring. The reaction was extracted with EtOAc (3 × 30 mL), and the combined organic phase washed with saturated brine (40 mL), dried over MgSO_4_, filtered, and the solvent removed *in vacuo* to afford the crude material, which was purified by column chromatography (see individual compound data for chromatography conditions).

#### General Procedure 2

The conversion of furans into pyrrolinones using artUPO, with slow addition of amine with hydrogen peroxide (used for most Scheme 4 results)

To a 100 mL round-bottom flask containing a stirrer bar was added KPi buffer (53.0 mL per mmol of furan, 100 mM, pH 7.00), artUPO secretate (2.00 mL per mmol of furan), and a solution of furan **5b** (1.00 equiv) dissolved in MeCN (20% v/v of reaction mixture). The reaction was initiated by the slow addition (syringe pump addition) of an aqueous H_2_O_2_ solution (1.50–2.00 equiv in 4.00 mL water, 0.50 mL/h) alongside infusion of an aqueous amine or amine hydrochloride solution (1.50–2.00 equiv in 4.00 mL water, 0.75 mL/h) followed by overnight stirring. The reaction was extracted with EtOAc (3 × 30 mL), and the combined organic phase washed with saturated brine (40 mL), dried over MgSO_4_, filtered, and the solvent removed *in vacuo* to afford the crude material, which was purified by column chromatography (see individual compound data for chromatography conditions).

#### General Procedure 3

The conversion of furans into pyrrolinones using artUPO: Amine added prior to hydrogen peroxide infusion (used for selected Scheme 4 results)

To a 100 mL round-bottom flask with containing a stirrer bar was added KPi buffer (40 mL per mmol of furan, 100 mM, pH 7.00) and the amine hydrochloride salt or free amine (2.00 equiv) at RT. After stirring for 10 min (s), artUPO secretate (1.33 mL per mmol of furan), furan **5b** (1.00 equiv) in MeCN (20% v/v of reaction mixture) was added. The reaction was initiated by the slow addition (syringe pump addition) of an aqueous H_2_O_2_ solution (1.50 – 2.00 equiv in 4.00 mL, 0.75 mL/h) followed by overnight stirring. The reaction was extracted with EtOAc (3 × 30 mL), and the combined organic phase washed with saturated brine (40 mL), dried over MgSO_4_, filtered, and the solvent removed *in vacuo* to afford the crude material, which was purified by column chromatography (see individual compound data for chromatography conditions).

## Conclusion

In summary, the first biocatalytic method for the conversion of furans **5** and amines **6** into pyrrolinones **7** has been developed, catalyzed by artUPO. The process is broad in scope (33 examples) and all biotransformations were performed on preparative scale, using a simple RT protocol. Crucially, no exogenous cofactors are required, with H_2_O_2_ used as the stoichiometric oxidant, added dropwise via syringe pump addition. A diverse array of functionalized pyrrolinones is accessible using the same general method, with an especially broad scope demonstrated with respect to the amine partner, attesting to the UPO’s broad tolerance of reactive amines. The overall cascade is proposed to operate via an initial UPO-catalyzed oxidative furan ring opening, followed by condensation and rearrangement with the amine, likely outside the UPO active site. This new reaction adds to the ever-growing toolbox of useful, preparative scale biotransformations achievable using UPOs.

## Supplementary Material



## Notes

Experimental procedures, additional reaction optimization details, compound characterization data, and NMR spectra (PDF)

## Abbreviations

UPO: unspecific peroxygenase
P450s: cytochromes P450
Mb: myoglobin
