# Supplementary material for: Biocatalytic Conversion of Furans into Pyrrolinones Using a Class I Unspecific Peroxygenase
Source: ACS Catal. 2025 Sep 4;15(18):16115–20. doi: 10.1021/acscatal.5c05307 (PMC12455563; doi:10.1021/acscatal.5c05307)
Supplement: Supplementary file 1 [file cs5c05307_si_001.pdf]

Biocatalytic Conversion of Furans into Pyrrolinones Using a Class I Unspecific Peroxygenase.

Benjamin Melling,[a] Katy A. S. Cornish, Nicholas P. Mulholland,[b] Jared Cartwright,[c] William P. Unsworth*[a] and Gideon Grogan*[a]

[a]Department of Chemistry, University of York, Heslington, York, U.K., YO10 5DD.
[b] Syngenta, Jealott's Hill International Research Centre, Bracknell, Berkshire RG42 6EY, UK
[c] Department of Biology, University of York, Heslington, York, U.K., YO10 5DD.

*william.unsworth@york.ac.uk; gideon.grogan@york.ac.uk

Supporting Information

Table of Contents

1) General Information	S3
2) Experimental Procedures	S4–S8
3) Compound data	S10–S29
4) ^{1}H and ^{13}C NMR spectra	S30–S63
5) References	S64

1. General Information

All reactions were performed in oven-dried glassware under a nitrogen atmosphere. Unless specified, all reagents and starting materials were purchased from commercial companies and used as received. Anhydrous solvents were freshly obtained from pure solvent system following standard procedures. Analytical thin layer chromatography (TLC) was performed using pre-coated silica gel plates. A Razel A 99 syringe pump was used for the slow addition of solutions. Visualisation was achieved by UV light (254 nm) or $KMnO_4$ and ninhydrin as stain. Flash chromatography was performed using silica gel and gradient solvent system (eluent: hexane: ethyl aetate/hexane: DCM). ^{1}H and ^{13}C NMR spectra were recorded on 400MHz Jeol ECS and Bruker AV and AM spectrometers. Chemical shifts (ppm) were recorded with tetramethylsilane (TMS) as the internal reference standard. Multiplicities are given as: s (singlet), br s (broad singlet), d (doublet), t (triplet), dd (doublet of doublets), dt (double of triples), ddd (doublet of doublet of doublets), ddt (doublet of doublet of triplets), dtt (doublet of triplet of triplets), ddq (doublet of doublet of quintets), dddd (doublet of doublet of doublet of doublets), dtd (doublet of triplet of doublets), dt (doublet of triplets), dp (doublet of pentents), dq (doublet of quintets), td (triplet of doublets), tdd (triplet of doublet of doublets), tt (triplet of triplets), qd (quintet of doublets), qt (quintet of triplets) or m (multiplet). The number of protons (*n*) for a given resonance is indicated by *n*H and coupling constants are reported as a *J* value in Hz. High resolution mass spectra (HRMS) were obtained on a LC/HRMS TOF spectrometer using simultaneous electrospray (ESI).

The cloning and expression of the artUPO used in this study and its preparation from fermentations of *Pichia pastoris* has been described previously.[1]

2) Experimental Procedures

2.1 Synthesis of 2-methyl-5-oxo-N-phenyl-4,5-dihydro-1H-pyrrole-3-carboxamide (7a)

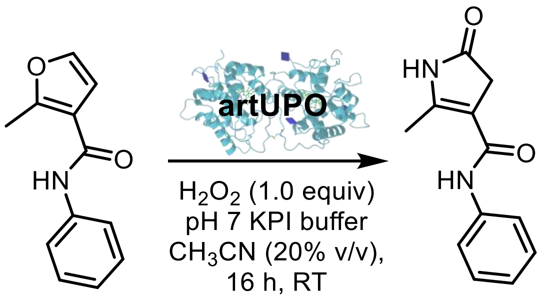

To a two-neck 100 mL round bottom flask, fitted with a reflux condenser, and containing a magnetic stirrer bar, was added KPi buffer (20.0 mL, pH 7.00) then artUPO secretate (1.00 mL) at 30 °C. A solution of Fenfuram **5a** in MeCN (0.250 mmol, 5.00 mL) was added before the reaction was initiated by the slow, continuous addition of an aqueous H_2O_2 solution (46.0 µL of a 30% H_2O_2 solution diluted up to 1.20 mL in water, 0.450 mmol, 0.8 mL/h). The reaction was monitored by LC/MS, and after full consumption of the starting material (2 h after the H_2O_2 infusion was complete) the reaction was extracted with EtOAc (3 x 30 mL) and the combined organic phases washed with saturated brine (30 mL), dried over $MgSO_4$, filtered, and the solvent removed *in vacuo* to afford the crude material an orange oil. Purification via flash chromatography on silica gel (gradient eluent: cyclohexane/EtOAc = 3:7 to EtOAc) afforded **7a** as an orange solid (19 mg; 35% yield). ^{1}H NMR (400 MHz, *methanol-d$_4$*) δ 7.57 – 7.52 (m, 2H; (CONH(C=C***H***)), 7.33 – 7.28 (m, 2H; CONH(C=CH=C***H***)), 7.12 – 7.07 (m, 1H; CONH(C=CH=CH=C***H***)), 3.44 (q, *J* = 2.3 Hz, 2H; NC(O)C***H$_2$***), 2.36 (t, *J* = 2.3 Hz, 3H; C(C***H$_3$***)=C); ^{13}C NMR (101 MHz, *methanol-d$_4$*) δ 179.4 (N***C***(O)CH$_2$), 165.2 (C=C(***C***ONHPh)), 152.3 (***C***(CH$_3$)=C), 139.7 (CONH(***C***=CH)), 129.7 (CONH(C=CH=***C***H)), 125.3 (CONH(C=CH=CH=***C***H)), 122.5 (CONH(C=***C***H)), 107.6 (C=***C***(CONHPh)), 38.1 (NC(O)***C***H$_2$), 13.3 (C(***C***H$_3$)=C); ^{14}N NMR (61 MHz, *methanol-d$_4$*) δ 156.84 (s, ***N***C(O)CH$_2$), 129.16 (s, C=C(CO***N***HPh)); HRMS (ESI, m/z) calculated formula for $C_{12}H_{12}N_2NaO_2$ (M Na)$^+$ 239.0791, found 239.0795; IR : v_{max}/cm^{-1} 3241, 1713, 1620, 1531, 1441, 1334, 1228, 753.

2.2 Synthesis of N-(4-hydroxyphenyl)-2-methylfuran-3-carboxamide (8)

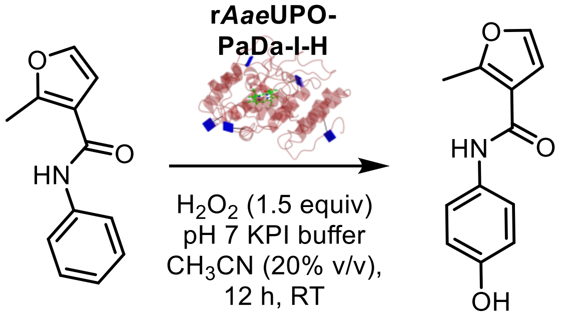

To a 100 mL round bottom flask containing a magnetic stirrer bar, was added KPi buffer (40.0 mL, pH 7.00) then artUPO secretate (1.00 mL) at RT. A solution of Fenfuram **5a** in MeCN (0.75 mmol, 10.0 mL) was added before the reaction was initiated by the slow, continuous addition of an aqueous H_2O_2 solution (116 µL of a 30% H_2O_2 solution diluted up to 4.0 mL with water, 1.13 mmol, 0.25 mL/h). After overnight stirring, the reaction was extracted with EtOAc (3 x 30 mL) and the combined organic phase washed with saturated brine (30 mL), dried over $MgSO_4$, filtered, and the solvent removed *in vacuo* to afford the crude material a yellow oil. Purification via flash chromatography on silica gel (eluent: hexane/EtOAc = 3:1) afforded **8** as a white solid (50 mg; 31% yield); M.p. 65 °C (decomp.); ^{1}H NMR (400 MHz, *methanol-d$_4$*) δ 7.40 – 7.35 (m, 3H; C(O)NH(C=C***H***=CH) and ***H***CO), 6.85 (d, *J* = 2.2 Hz, 1H; C***H***=HCO), 6.79 – 6.75 (m, 2H; C(O)NH(C=CH=C***H***)), 2.56 (s, 3H; C=C(C***H$_3$***)); ^{13}C NMR (101 MHz, *methanol-d$_4$*) δ 164.8 ((CH_3)***C***=C(CONHPh)), 158.6 (***C***(O)NHPh), 155.7 (C(O)NH(C=CH=CH=***C***(OH)), 141.7, 131.3 (CONH(***C***=CH)), 124.6 (HC=H***C***O), 117.2 ((CH_3)C=***C***(CONHPh)), 116.2 (C(O)NH(C=CH=***C***H)), 110.0 (H***C***=HCO), 13.6 (C=C(***C***H_3)); HRMS (ESI, m/z) calculated for $C_{12}H_{11}NNaO_3$ (M Na)$^+$ 240.0631, found 240.0634; IR : v_{max}/cm^{-1} 3313, 2921, 2852, 1607, 1513, 1228, 1095, 825, 732.

General Procedure 2.3. Amine Added Prior to Hydrogen Peroxide Infusion (Scheme 3 results)

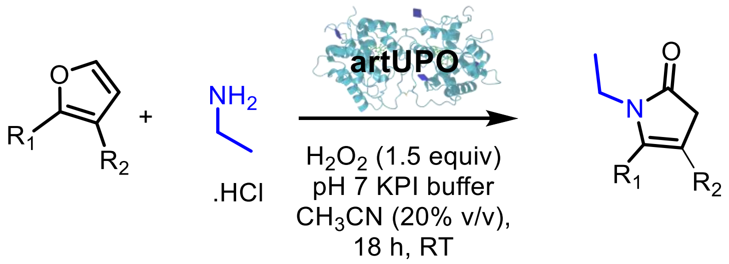

To a 100 mL round bottom flask with containing a stirrer bar was added KPi Buffer (24 mL, 100 mM, pH 7.00) and ethylamine hydrochloride salt (1.80 mmol, 146 mg, 2.00 eq.) at RT. After stirring for 10 min, artUPO secretate (1.00 mL) was added, followed by addition of a solution of the corresponding furan (0.900 mmol) in MeCN (6.00 mL). The reaction was initiated by the slow addition (syringe pump addition) of an aqueous H_2O_2 solution (139 µL of a 30% H_2O_2 solution diluted up to 4.00 mL with water, 1.35 mmol, 0.75 mL/h) followed by overnight stirring. The reaction was extracted with EtOAc (3 x 30 mL), and the combined organic phase washed with saturated brine (40 mL), dried over $MgSO_4$, filtered, and the solvent removed *in vacuo* to afford the crude material, which was purified by column chromatography (see individual compound data for chromatography conditions).

General Procedure 2.4. Slow Addition of Amine with Hydrogen Peroxide

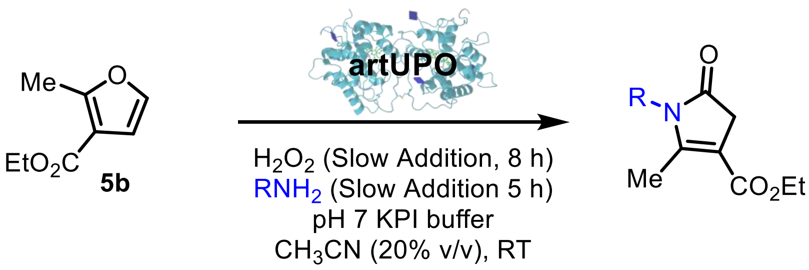

To a 100 mL round bottom flask containing a stirrer bar was added KPi Buffer (53.0 mL per mmol of furan, 100 mM, pH 7.00), artUPO secretate (2.00 mL per mmol of furan), and a

solution of furan **5b** (1.00 eq.) dissolved in MeCN (20% v/v of reaction mixture). The reaction was initiated by the slow addition (syringe pump addition) of an aqueous H_2O_2 solution (1.50 – 2.00 eq. in 4.00 mL water, 0.50 mL/h) alongside infusion of an aqueous amine or amine hydrochloride solution (1.50 – 2.00 eq. in 4.00 mL water, 0.75 mL/h) followed by overnight stirring. The reaction was extracted with EtOAc (3 x 30 mL), and the combined organic phase washed with saturated brine (40 mL), dried over $MgSO_4$, filtered, and the solvent removed *in vacuo* to afford the crude material, which was purified by column chromatography (see individual compound data for chromatography conditions).

General Procedure 2.5. Amine Added Prior to Hydrogen Peroxide Infusion (15 mM scale)

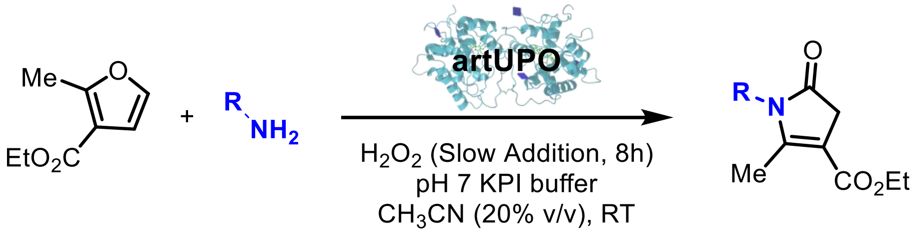

To a 100 mL round bottom flask with containing a stirrer bar was added KPi Buffer (40 mL per mmol of furan, 100 mM, pH 7.00) and the amine hydrochloride salt or free amine (2.00 eq.) at RT. After stirring for 10 min (s), artUPO secretate (1.33 mL per mmol of furan), furan (1.00 eq.) in MeCN (20% v/v of reaction mixture) was added. The reaction was initiated by the slow addition (syringe pump addition) of an aqueous H_2O_2 solution (1.50 – 2.00 eq. in 4.00 mL, 0.75 mL/h) followed by overnight stirring. The reaction was extracted with EtOAc (3 x 30 mL), and the combined organic phase washed with saturated brine (40 mL), dried over $MgSO_4$, filtered, and the solvent removed *in vacuo* to afford the crude material, which was purified by column chromatography (see individual compound data for chromatography conditions).

2.6 Optimisation of the Amine Addition Method

To investigate the best conditions for amine addition, we performed the oxidation in the presence of benzylamine hydrochloride (Table S1) according to the addition methods outlined in general procedure **2.3** (first entry) and **2.4** (second and third entries) respectively. We then compared the two methods of amine addition against 4 additional amine substrates using general procedure **2.4** and **2.5** (Table S2).

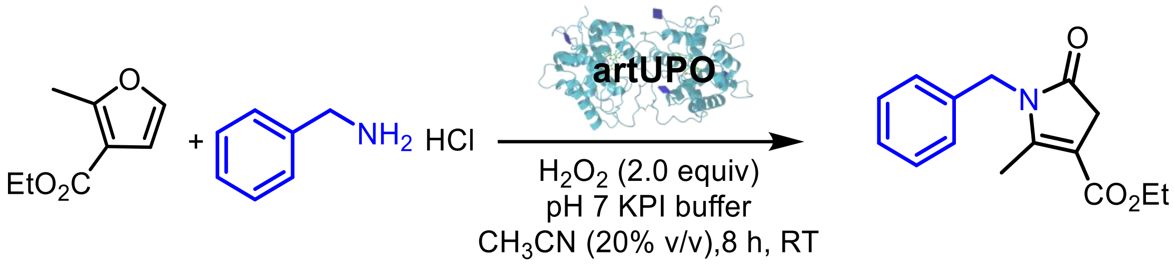

**Furan/ mmol dm^{-3}**	**Amine (equiv.)**	**Method of Amine Addition**	**Method of H_2O_2 addition**	**Yield (%)**
30	2.0	Added as a single portion at the start before H_2O_2	Slow addition (8 h)	9
30	2.0	Slow Addition (5 h)	Slow addition (8 h)	19
15	1.5	Slow Addition (5 h)	Slow addition (8 h)	65

Table S1

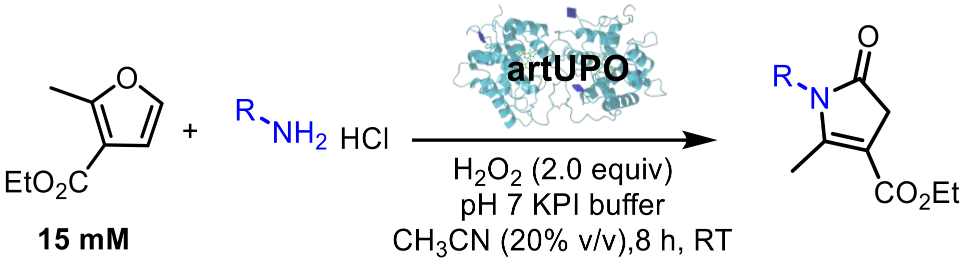

Amine	Method of Amine Addition	Yield (%)
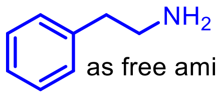 as free amine	Added as a single portion at the start before H_2O_2	0
	Slow Addition (5 h)	55
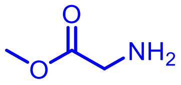	Added as a single portion at the start before H_2O_2	0
	Slow Addition (5 h)	75
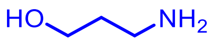	Added as a single portion at the start before H_2O_2	0
	Slow Addition (5 h)	26
as free amine 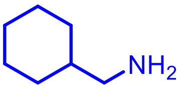	Added as a single portion at the start before H_2O_2	32
	Slow Addition (5 h)	28

Table S2

3) Compound data

1-Ethyl-2-methyl-5-oxo-N-phenyl-4,5-dihydro-1H-pyrrole-3-carboxamide (7b)

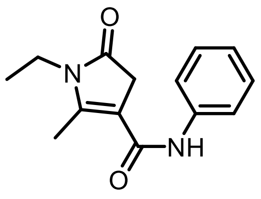

Synthesised from 2-methyl-*N*-phenyl-3-furancarboxamide **5a** (0.9 mmol, 196 mg) and ethylamine hydrochloride (1.80 mmol, 146 mg) according to general procedure **2.3**. Purification via flash chromatography on silica gel (eluent: hexane/ EtOAc = 3:2) afforded the title compound as a pale-yellow solid (100 mg, 45% yield); M.p. 130 – 133 °C; ^{1}H NMR (400 MHz, *chloroform-d*) δ 7.53 – 7.49 (m, 2H; (NH)CCH=C***H***=CH), 7.35 – 7.28 (m, 2H; (NH)CC***H***=CH=CH), 7.12 – 7.06 (m, 2H; ((N***H***)CCH=CH=C***H***), 3.59 (q, *J* = 7.2 Hz, 2H; NC***H***$_2$CH$_3$), 3.30 (q, *J* = 2.4 Hz, 2H; NC(O)C***H***$_2$), 2.54 (t, *J* = 2.4 Hz, 3H; C=CC***H***$_3$), 1.19 (t, *J* = 7.2 Hz, 3H; NCH$_2$C***H***$_3$); ^{13}C NMR (101 MHz, *chloroform-d*) δ 174.5 (N***C***(O)CH$_2$), 162.4 (C=C(***C***ONHPh)), 153.0 (C=***C***(CH$_3$)), 138.0 ((NH)***C***CH), 129.1 ((NH)C***C***H=CH=CH)), 124.4 ((NH)CCH=CH=***C***H), 120.5 ((NH)CCH=***C***H=CH), 105.2 (C=***C***(CONHPh)), 36.4 (NC(O)***C***H$_2$), 35.1 (N***C***H$_2$CH$_3$), 14.6 (NCH$_2$***C***H$_3$), 12.2 (C=C(***C***H$_3$)); HRMS (ESI, m/z) calculated for $C_{14}H_{16}N_2NaO_2$ (M Na)$^+$ 267.1104, found 267.1107; IR : v_{max}/cm^{-1} 1710, 1655, 1531, 1489, 1323, 1227, 1159, 760, 695, 574.

Ethyl 1-ethyl-2-methyl-5-oxo-4,5-dihydro-1H-pyrrole-3-carboxylate (7c)

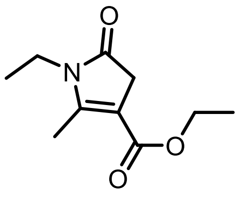

Synthesised from 2-methyl-3-ethoxycarbonyl-furan **5b** (0.90 mmol, 139 mg) ethylamine hydrochloride (1.80 mmol, 146 mg) according to general procedure **2.3**. Purification via flash chromatography on silica gel (eluent: hexane/ EtOAc = 3:2) afforded the title compound as a yellow solid (139 mg, 79% yield); M.p. 68 – 70 °C; ^{1}H NMR (400 MHz, *chloroform-d*) δ 4.18 (q, *J* = 7.1 Hz, 2H; OC***H***$_2$CH$_3$), 3.57 (q, *J* = 7.1 Hz, 2H; NC***H***$_2$), 3.24 (q, *J* = 2.3 Hz, 2H; NC(O)C***H***$_2$), 2.45 (t, *J* = 2.3 Hz, 3H; C=CC***H***$_3$), 1.28 (t, *J* = 7.1 Hz, 3H; OCH$_2$C***H***$_3$), 1.17 (t, *J* = 7.1 Hz, 3H; NCH$_2$C***H***$_3$); ^{13}C NMR (101 MHz, *chloroform-d*) δ 176.0 (N***C***(O)CH$_2$), 164.4 (***C***O$_2$Et), 154.0 (C=***C***(CH$_2$CH$_3$)), 103.6

(C=***C***(CO$_2$Et)), 59.9 (O***C***H$_2$CH$_3$), 36.8 (NC(O)***C***H$_2$), 35.0 (N***C***H$_2$), 14.7, 14.6, 12.3 (C=C(***C***H$_3$)); HRMS (ESI, m/z) calculated for $C_{10}H_{15}NNaO_3$ (M Na)$^+$ 220.0950, found 220.0949; IR: v_{max}/cm^{-1} 2982, 1684, 1627, 1395, 1318, 1212, 1063, 992, 787, 739, 562.

Ethyl 1,2-diethyl-5-oxo-4,5-dihydro-1H-pyrrole-3-carboxylate (7d)

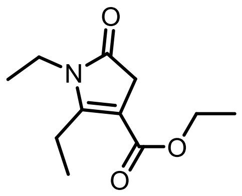

Synthesised from ethyl 2-ethyl-3-furoate **5c** (0.90 mmol, 152 mg) and ethylamine hydrochloride (1.80 mmol, 146 mg) according to general procedure **2.3**. Purification via flash chromatography on silica gel (eluent: hexane/ EtOAc = 3:2) afforded the title compound as a yellow oil (128 mg, 66% yield); ^{1}H NMR (400 MHz, *chloroform-d*) δ 4.19 (q, *J* = 7.1 Hz, 2H; OC***H***$_2$CH$_3$), 3.56 (q, *J* = 7.2 Hz, 2H; NC***H***$_2$CH$_3$), 3.24 (t, *J* = 1.2 Hz, 2H; NC(O)C***H***$_2$), 2.85 (qt, *J* = 7.6, 1.2 Hz, 2H; C=C(C***H***$_2$CH$_3$)), 1.28 (t, *J* = 7.1 Hz, 3H; OCH$_2$C***H***$_3$), 1.21 (t, *J* = 7.6, 3H; C=C(CH$_2$C***H***$_3$)), 1.20 (t, *J* = 7.2 Hz, 3H; NCH$_2$C***H***$_3$); ^{13}C NMR (101 MHz, *chloroform-d*) δ 176.4 (N***C***(O)CH$_2$), 164.0 (***C***O$_2$Et), 159.7 (C=***C***(CH$_2$CH$_3$)), 102.3 (C=***C***(CO$_2$Et)), 59.8 (O***C***H$_2$CH$_3$), 36.8 (NC(O)***C***H$_2$), 35.1 (N***C***H$_2$), 19.4 (N***C***H$_2$), 14.8 (C=C(CH$_2$***C***H$_3$)), 14.6 (OCH$_2$***C***H$_3$), 13.0 (NCH$_2$***C***H$_3$); HRMS (ESI, m/z) calculated for $C_{11}H_{17}NNaO_3$ (M Na)$^+$ 234.1101, found 234.1104; IR : v_{max}/cm^{-1} 2980, 1724, 1683, 1619, 1462, 1393, 1234, 1084, 1025, 757, 538.

Ethyl 1-ethyl-2,4-dimethyl-5-oxo-4,5-dihydro-1H-pyrrole-3-carboxylate (7e)

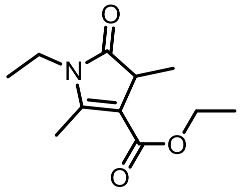

Synthesised from ethyl 2,4-dimethylfuran-3-carboxylate **5d**[2] (0.90 mmol, 151 mg) ethylamine hydrochloride (1.80 mmol, 146 mg) according to general procedure **2.3**. Purification via flash chromatography on silica gel (eluent: hexane/ EtOAc = 3:2) afforded the title compound as a white solid (161 mg, 85% yield); Mp 56 – 58 °C; ^{1}H NMR (400 MHz, *chloroform-d*) δ 4.22 (dq, *J* = 18.0, 7.2 Hz, 1H; OC***H***$_2$), 4.15 (dq, *J* = 18.0, 7.2 Hz, 1H; OC***H***$_2$), 3.56 (q, *J* = 7.2 Hz, 2H; NC***H***$_2$CH$_3$), 3.22 – 3.15 (m, 1H; NC(O)C***H***(CH$_3$)), 2.44 (d, *J* = 2.2 Hz, 3H; C=CC***H***$_3$), 1.36 (d, *J* = 7.5 Hz, 3H; NC(O)CH(C***H***$_3$)), 1.29 (t, *J* = 7.2 Hz, 3H; OCH$_2$***C***H$_3$), 1.16 (t, *J* = 7.2 Hz, 3H; NCH$_2$C***H***$_3$); ^{13}C

NMR (101 MHz, *chloroform-d*) δ 179.9 ($N\boldsymbol{C}(O)CH_2$), 164.5 ($\boldsymbol{C}O_2Et$), 153.2 ($C{=}\boldsymbol{C}(CH_3)$), 109.6 ($C{=}\boldsymbol{C}(CO_2Et)$), 59.6 ($O\boldsymbol{C}H_2CH_3$), 41.9 ($NC(O)\boldsymbol{C}H(CH_3)$), 34.8 ($N\boldsymbol{C}H_2CH_3$), 15.5 ($NC(O)CH(\boldsymbol{C}H_3)$), 14.6, 14.5, 12.2 ($C{=}C(\boldsymbol{C}H_3)$); HRMS (ESI, m/z) calculated for $C_{11}H_{17}NNaO_3$ $(M\ Na)^+$ 234.1101, found 234.1110; IR : v_{max}/cm^{-1} 2937, 1711, 1686, 1615, 1366, 1253, 1164, 1033, 790, 567, 456.

4-Acetyl-1-ethyl-5-methyl-1,3-dihydro-2H-pyrrol-2-one (7f)[3]

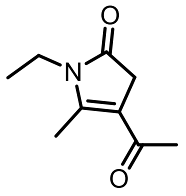

Synthesised from 3-acetyl 2-methyl furan **5e**[4] (0.90 mmol, 112 mg) ethylamine hydrochloride (1.80 mmol, 146 mg) according to general procedure **2.3**. Purification via flash chromatography on silica gel (gradient eluent: hexane/ EtOAc = 3:2 to EtOAc); as a yellow solid (90 mg, 59% yield); ^{1}H NMR (400 MHz, *chloroform-d*) δ 3.59 (q, *J* = 7.2 Hz, 2H; $NC\boldsymbol{H_2}CH_3$), 3.31 (q, *J* = 2.4 Hz, 2H; $NC(O)C\boldsymbol{H_2}$), 2.48 (t, *J* = 2.3 Hz, 3H; $C{=}C(C\boldsymbol{H_3})$), 2.19 (s, 3H; $C(O)\boldsymbol{C}H_3$), 1.18 (t, *J* = 7.2 Hz, 3H; $NCH_2C\boldsymbol{H_3}$); ^{13}C NMR (101 MHz, *chloroform-d*) δ 193.0 ($\boldsymbol{C}(O)CH_3$), 175.3 ($N\boldsymbol{C}(O)CH_2$), 153.9 ($C{=}\boldsymbol{C}(CH_3)$), 112.1 ($C{=}\boldsymbol{C}(COCH_3)$), 37.4, 34.9 ($N\boldsymbol{C}H_2CH_3$), 29.7 ($C(O)\boldsymbol{C}H_3$), 14.5 ($NCH_2\boldsymbol{C}H_3$), 12.9 ($C{=}C(\boldsymbol{C}H_3)$).

1-Ethyl-5-hexyl-1,3-dihydro-2H-pyrrol-2-one (7i)

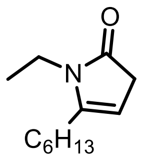

Synthesised from 2-hexylfuran **5f** (1.00 mmol, 152 mg) and ethylamine hydrochloride (2.00 mmol, 162 mg) according to general procedure **2.3** at 0 °C. Purification via flash chromatography on silica gel (eluent: hexane/ EtOAc = 2:3); as a yellow oil (102 mg, 52% yield); ^{1}H NMR (400 MHz, *chloroform-d*) δ 4.64 (tt, *J* = 7.4, 2.2 Hz, 1H; $C{=}C\boldsymbol{H}$), 3.49 (q, *J* = 7.4 Hz, 2H; $NC\boldsymbol{H_2}CH_3$), 2.59 (dddt, *J* = 9.9, 6.1, 2.3, 1.1 Hz, 2H), 2.49 – 2.42 (m, 2H), 2.03 – 1.96 (m, 2H; $NC(O)C\boldsymbol{H_2}$), 1.43 – 1.23 (m, 4H), 1.10 (t, *J* = 7.4 Hz, 3H; $NCH_2C\boldsymbol{H_3}$), 0.91 – 0.87 (m, 3H; $=C((CH_2)_5C\boldsymbol{H_3})$; ^{13}C NMR (101 MHz, *chloroform-d*) δ 175.3 ($N\boldsymbol{C}(O)CH_2$), 138.8 ($\boldsymbol{C}(C_6H_{13}){=}CH$), 100.8 ($C{=}\boldsymbol{C}H$), 34.6 ($N\boldsymbol{C}H_2CH_3$), 31.6 ($\boldsymbol{C}H_2$), 30.0 ($\boldsymbol{C}H_2$), 29.1 ($\boldsymbol{C}H_2$), 26.8 ($NC(O)\boldsymbol{C}H_2$), 22.7 ($\boldsymbol{C}H_2$), 21.5 ($\boldsymbol{C}H_2$), 14.2 ($=C((CH_2)_5\boldsymbol{C}H_3)$), 12.0

($NCH_2\boldsymbol{C}H_3$); HRMS (ESI, m/z) calculated for $C_{12}H21NNaO$ (M Na) $^+$ 218.1521, found 218.1523; IR: v_{max}/cm^{-1} 2928, 1717, 1671, 1410, 1343, 1241, 1148, 653.

Ethyl 1-benzyl-2-methyl-5-oxo-4,5-dihydro-1H-pyrrole-3-carboxylate (7j)

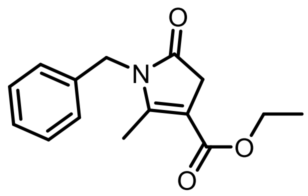

Synthesised from 2-methyl-3-ethoxycarbonyl-furan **5b** (0.230 mmol, 35 mg) and benzyl amine hydrochloride (50.0 mg, 0.350 mmol, 1.50 eq.) according to general procedure **2.4** using KPi Buffer (12 mL), MeCN (3 mL), and aq. H_2O_2 solution (0.460 mmol in 4.00 mL H_2O); Purification via flash chromatography on silica gel (eluent: hexane/ EtOAc = 7:3); as a yellow solid [3] (39 mg, 65% yield); ^{1}H NMR (400 MHz, *chloroform-d*) δ 7.35 – 7.30 (m, 2H), 7.28 (t, *J* = 1.6 Hz, 1H), 7.22 – 7.15 (m, 2H), 4.76 (s, 2H; $NC\boldsymbol{H_2}$), 4.18 (q, *J* = 7.1 Hz, 2H; $OC\boldsymbol{H_2}CH_3$), 3.37 (q, *J* = 2.3 Hz, 2H; $NC(O)C\boldsymbol{H_2}$), 2.33 (t, *J* = 2.3 Hz, 3H; $C{=}CC\boldsymbol{H_3}$), 1.28 (t, *J* = 7.1 Hz, 3H; $OCH_2C\boldsymbol{H_3}$); ^{13}C NMR (101 MHz, *chloroform-d*) δ 176.2 ($N\boldsymbol{C}(O)CH_2$), 164.4 ($\boldsymbol{C}O_2Et$), 154.2 ($C{=}\boldsymbol{C}CH_3$), 136.6, 129.0, 127.9, 127.0, 103.9 ($C{=}\boldsymbol{C}(CO_2Et)$), 60.0 ($O\boldsymbol{C}H_2CH_3$), 43.6, 36.8 ($NC(O)\boldsymbol{C}H_2$), 14.6 ($OCH_2\boldsymbol{C}H_3$), 12.8 ($C{=}C\boldsymbol{C}H_3$).

Ethyl 1-cyclopropyl-2-methyl-5-oxo-4,5-dihydro-1H-pyrrole-3-carboxylate (7k)

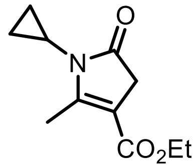

Synthesised from 2-methyl-3-ethoxycarbonyl-furan **5b** (0.750 mmol, 116 mg) and cyclopropyl amine hydrochloride (138 mg, 1.50 mmol, 2.00 eq.) according to general procedure **2.4** using KPi Buffer (40 mL), MeCN (10 mL), and aq. H_2O_2 solution (1.50 mmol in 4.00 mL H_2O). Purification via flash chromatography on silica gel (eluent: hexane/EtOAc = 7:3); as a yellow solid (100 mg, 64% yield); Mp. 71 – 72 °C; ^{1}H NMR (400 MHz, *chloroform-d*) δ 4.17 (q, 2H, *J* = 6.7 Hz; $OC\boldsymbol{H_2}CH_3$), 3.19 (q, *J* = 2.4 Hz, 2H; $NCOC\boldsymbol{H_2}$), 2.52 (t, *J* = 2.4 Hz, 3H; $C{=}CC\boldsymbol{H_3}$), 2.50 – 2.42 (m, 1H; $NC\boldsymbol{H}$), 1.27 (t, *J* = 6.7 Hz, 3H; $OCH_2C\boldsymbol{H_3}$), 1.04 – 0.97 (m, 2H), 0.88 – 0.80 (m, 2H); ^{13}C NMR (101 MHz, *chloroform-d*) δ 176.7 ($N\boldsymbol{C}O$), 164.4 (***CO2Et***), 156.1, 103.3, 59.9, 37.0 ($NCO\boldsymbol{C}H_2$), 22.6, 14.6 ($OCH_2\boldsymbol{C}H_3$), 13.1 ($C{=}C\boldsymbol{C}H_3$), 6.9; HRMS (ESI, m/z) calculated for

$C_{11}H_{15}NNaO_3$ (M Na)$^+$ 232.0950, found 232.0940; IR : v_{max}/cm^{-1} 1723, 1681, 1629, 1481, 1364, 1328 , 1202, 1052, 752, 556.

Ethyl 1-cyclobutyl-2-methyl-5-oxo-4,5-dihydro-1H-pyrrole-3-carboxylate (7l)

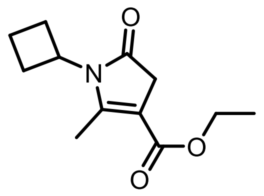

Synthesised from 2-methyl-3-ethoxycarbonyl-furan **5b** (0.750 mmol, 116 mg) and cyclobutylamine hydrochloride (161 mg, 1.50 mmol, 2.00 eq.) according to general procedure **2.4** using KPi Buffer (40.0 mL), MeCN (10.0 mL), and aq. H_2O_2 solution (1.50 mmol in 4.00 mL H_2O). Purification via flash chromatography on silica gel (eluent: hexane/EtOAc = 7:3); as a yellow oil (69 mg, 41% yield); ^{1}H NMR (400 MHz, *chloroform-d*) δ 4.26 – 4.19 (m, 1H; NC***H***), 4.17 (q, *J* = 7.1 Hz, 2H; OC***H₂***CH₃), 3.20 (q, *J* = 2.4 Hz, 2H; NCOC***H₂***), 2.94 – 2.81 (m, 2H; (C***H***HCH₂C***H***H), 2.42 (t, *J* = 2.4 Hz, 3H; C=CC***H₃***), 2.25 – 2.12 (m, 2H; (CH***H***CH₂CH***H***)), 1.91 – 1.77 (m, 1H; CH₂CH***H***CH₂), 1.77 – 1.61 (m, 1H; CH₂C***H***HCH₂), 1.26 (t, *J* = 7.1 Hz, 3H; OCH₂C***H₃***); ^{13}C NMR (101 MHz, *chloroform-d*) δ 176.9 (N***C***O), 164.5 (***C***O₂Et), 154.9 (=***C***CH₃), 103.4 (C=***C***(CO₂Et)), 59.8 (O***C***H₂CH₃), 48.0 (N***C***H), 37.5 (NCO***C***H₂), 28.3 (***C***H₂CH₂***C***H₂), 15.4 (CH₂***C***H₂CH₂), 14.6 (OCH₂***C***H₃), 12.9 (C=C***C***H₃); HRMS (ESI, m/z) calculated for $C_{12}H_{17}NNaO_3$ (M Na)$^+$ 246.1106, found 246.1106; IR : v_{max}/cm^{-1} 2951, 1724, 1686, 1627, 1445, 1377, 1332, 1264, 1219, 1151, 1134, 1095, 1059, 1014, 755, 616.

Ethyl 1-cyclopentyl-2-methyl-5-oxo-4,5-dihydro-1H-pyrrole-3-carboxylate (7m)

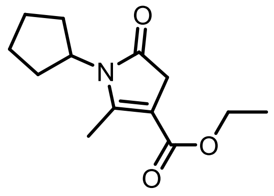

Synthesised from 2-methyl-3-ethoxycarbonyl-furan **5b** (0.600 mmol, 93.0 mg), cyclopentylamine hydrochloride (108 mg, 0.900 mmol, 1.5 eq.) according to general procedure **2.5** using KPi Buffer (24.0 mL), MeCN (6 mL), and aq. H_2O_2 solution (1.20 mmol in 4.00 mL H_2O). Purification via flash chromatography on silica gel (eluent: hexane/EtOAc = 7:3); as a yellow oil (43 mg, 30% yield) ^{1}H NMR (400 MHz, *chloroform-d*) δ 4.26 – 4.07 (m, 3H), 3.21 (q, *J* = 2.4 Hz, 2H; NC(O)C***H₂***), 2.46 (t, *J* = 2.4 Hz, 3H), 2.14 – 2.01 (m, 2H), 1.98 – 1.76 (m, 4H), 1.65 – 1.53 (m, 3H), 1.37 –

1.17 (m, 6H), 0.91 – 0.84 (m, 1H); ^{13}C NMR (101 MHz, *chloroform-d*) δ 176.3, 164.5, 155.1, 103.6, 59.8, 54.0, 37.4, 29.5, 25.3, 22.8, 14.6, 13.0; HRMS (ESI, m/z) calculated for $C_{13}H_{19}NNaO_3$ (M Na)$^+$ 260.1263, found 260.1260; IR : v_{max}/cm^{-1} 2980, 1723, 1693, 1629, 1404, 1216, 1148, 1062, 756.

Ethyl 1-(cyclobutylmethyl)-2-methyl-5-oxo-4,5-dihydro-1H-pyrrole-3-carboxylate (7n)

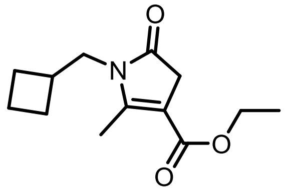

Synthesised from 2-methyl-3-ethoxycarbonyl-furan **5b** (0.750 mmol, 116 mg) and cyclobutylmethylamine hydrochloride (188 mg, 1.50 mmol, 2.00 eq) according to general procedure **2.5** using KPi Buffer (32.0 mL), MeCN (8.00 mL), and aq. H_2O_2 solution (1.50 mmol in 4.00 mL H_2O). Purification via flash chromatography on silica gel (gradient eluent: hexane/EtOAc = 1:4 to hexane/EtOAc = 7:3); as a yellow solid (100 mg, 56% yield); Mp 99 – 101 °C; ^{1}H NMR (400 MHz, *chloroform-d*) δ 4.18 (q, *J* = 7.1 Hz, 2H; OC***H***$_2$CH$_3$), 3.54 (d, *J* = 7.3 Hz, 2H; NC***H***$_2$), 3.25 (q, *J* = 2.4 Hz, 2H; NC(O)C***H***$_2$), 2.54 (hept, *J* = 7.5 Hz, 1H; NCH$_2$C***H***), 2.42 (t, *J* = 2.4 Hz, 3H; C=CC***H***$_3$), 2.06 – 1.94 (m, 2H; CH***H***CHCH***H***), 1.89 – 1.81 (m, 2H; C***H***$_2$), 1.80 – 1.71 (m, 2H; C***H***HCHC***H***H), 1.28 (t, *J* = 7.1 Hz, 3H; OCH$_2$C***H***$_3$); ^{13}C NMR (101 MHz, *chloroform-d*) δ 176.5 (N***C***O), 164.5 (***C***O$_2$Et), 154.4 (C=***C***CH$_3$), 103.4 (C=***C***(CO$_2$Et)), 59.8 (O***C***H$_2$CH$_3$), 45.2 (N***C***H$_2$), 36.7 (NC(O)***C***H$_2$), 35.4 (NCH2CH), 26.4 (***C***H$_2$CH***C***H$_2$), 18.3 (***C***H$_2$), 14.6 (OCH$_2$***C***H$_3$), 12.6 (C=C***C***H$_3$); HRMS (ESI, m/z) calculated for $C_{13}H_{19}NNaO_3$ (M Na)$^+$ 260.1257, found 260.1265; IR : v_{max}/cm^{-1} 2972, 2939, 1713, 1687, 1623, 1398, 1214, 1062, 999, 844, 725, 573.

Ethyl 1-(cyclohexylmethyl)-2-methyl-5-oxo-4,5-dihydro-1H-pyrrole-3-carboxylate (7o)

Synthesised from 2-methyl-3-ethoxycarbonyl-furan **5b** (0.800 mmol, 123 mg) and cyclohexyl methylamine hydrochloride (134 mg, 1.20 mmol, 1.50 eq.) according to general procedure **2.5** using KPi Buffer (32.0 mL), MeCN (8.00 mL), and aq. H_2O_2 solution (1.60 mmol in 4.00 mL H_2O). Purification via flash chromatography on silica gel (gradient eluent: hexane/ EtOAc =

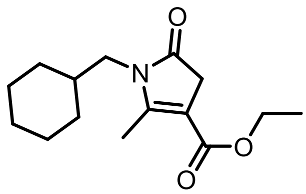

4:1 to hexane/EtOAc = 1:1); as a yellow oil (65 mg, 31% yield); ^{1}H NMR (400 MHz, *chloroform-d*) δ 4.17 (q, *J* = 7.1 Hz, 2H; OC***H***$_2$CH$_3$), 3.32 (d, *J* = 7.1 Hz, 2H; NC***H***$_2$), 3.25 (q, *J* = 2.4 Hz, 2H; NC(O)C***H***$_2$), 2.41 (t, *J* = 2.4 Hz, 3H), 1.78 – 1.54 (m, 6H; NCH$_2$C***H***), 1.27 (t, *J* = 7.2 Hz, 3H; OCH$_2$C***H***$_3$), 1.24 – 1.10 (m, 3H), 1.01 – 0.87 (m, 2H); ^{13}C NMR (101 MHz, *chloroform-d*) δ 176.4 (N***C***(O)CH$_2$), 164.4 (***C***O$_2$Et), 154.7 (C=***C***CH$_3$), 103.4 (C=***C***(CO$_2$Et)), 59.8 (O***C***H$_2$CH$_3$), 46.4 (N***C***H$_2$), 37.8 (NCH$_2$***C***H), 36.7 (NC(O)***C***H$_2$), 30.9 (***C***H$_2$), 26.3 (***C***H$_2$), 25.8 (***C***H$_2$), 14.6 (OCH$_2$***C***H$_3$), 12.7 (C=C***C***H$_3$); HRMS (ESI, m/z) calculated for $C_{15}H_{23}NNaO_3$ (M Na) $^{+}$ 288.1570, found 288.1581; IR : v_{max}/cm^{-1} 2925, 2852, 1725, 1691, 1626, 1449, 1396, 1224, 1059, 930, 722.

Ethyl 1-(cyanomethyl)-2-methyl-5-oxo-4,5-dihydro-1H-pyrrole-3-carboxylate (7p)

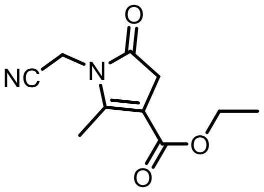

Synthesised from 2-methyl-3-ethoxycarbonyl-furan **5b** (0.750 mmol, 116 mg) and 2-aminoacetonitrile hydrochloride (138 mg, 1.50 mmol, 2.00 eq.) according to general procedure **2.4** using KPi Buffer (40.0 mL), MeCN (10.0 mL), and aq. H_2O_2 solution (1.50 mmol in 4.00 mL H_2O). Purification via flash chromatography on silica gel (gradient eluent: hexane/EtOAc = 7:3 to hexane/ EtOAc); as a yellow solid (84 mg, 54% yield); Mp 61 – 63 °C; ^{1}H NMR (400 MHz, *chloroform-d*) δ 4.47 (s, 2H; NC***H***$_2$CN), 4.21 (q, *J* = 7.1 Hz, 2H; OC***H***$_2$CH$_3$), 3.33 (q, *J* = 2.4 Hz, 2H; NCOC***H***$_2$), 2.56 (t, *J* = 2.4 Hz, 3H; C=CC***H***$_3$), 1.30 (t, *J* = 7.1 Hz, 3H; OCH$_2$C***H***$_3$); ^{13}C NMR (101 MHz, *chloroform-d*) δ 174.5 (N***C***O), 163.8 (***C***O$_2$Et), 150.6 (=***C***CH$_3$), 114.2(***C***N), 105.6 (C=***C***(CO$_2$Et)), 60.4 (O***C***H$_2$CH$_3$), 36.3 (NCO***C***H$_2$), 27.5 (N***C***H$_2$), 14.5 (OCH$_2$***C***H$_3$), 12.3 (C=C***C***H$_3$); HRMS (ESI, m/z) calculated for $C_{10}H_{12}N_2NaO_3$ (M Na) $^{+}$ 231.0746, found 231.0742; IR : v_{max}/cm^{-1} 2976, 1723, 1663, 1628, 1478, 1369, 1248, 1135, 1096, 906, 719, 584.

Ethyl 1-(2,2-difluoroethyl)-2-methyl-5-oxo-4,5-dihydro-1H-pyrrole-3-carboxylate (7q)

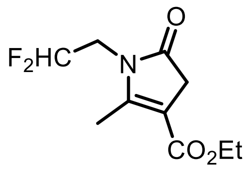

Synthesised from 2-methyl-3-ethoxycarbonyl-furan **5b** (0.750 mmol, 116 mg) and 2,2-difluoroethylamine hydrochloride (174 mg, 1.50 mmol, 2.00 eq.) according to general procedure **2.4** using KPi Buffer (40.0 mL), MeCN (10.0 mL), and aq. H_2O_2 solution (1.50 mmol in 4.00 mL H_2O). Purification via flash chromatography on silica gel (eluent: hexane/EtOAc = 7:3); as a light brown oil (73 mg, 42% yield); ^{1}H NMR (400 MHz, *chloroform-d*) δ 5.91 (tt, *J* = 55.8, 4.3 Hz, 1H; C***H***F$_2$), 4.20 (q, *J* = 7.1 Hz, 2H; OC***H$_2$***CH$_3$), 3.87 (td, *J* = 13.6, 4.3 Hz, 2H; C***H$_2$***CHF$_2$), 3.31 (q, *J* = 2.4 Hz, 2H; NCOC***H$_2$***), 2.45 (t, *J* = 2.4 Hz, 3H; C=CC***H$_3$***), 1.29 (t, *J* = 7.1 Hz, 3H; OCH$_2$C***H$_3$***); ^{13}C NMR (101 MHz, *chloroform-d*) δ 176.2 (N***C***O), 164.1 (***C***OOCH$_2$CH$_3$), 152.9 (N***C***=C), 112.6 (t, *J* = 243.5 Hz; ***C***HF$_2$), 104.8 (NC=***C***), 60.2 (O***C***H$_2$CH$_3$), 42.5 (t, *J* = 28.0 Hz; ***C***H$_2$CHF$_2$), 36.4 (NCO***C***H$_2$), 14.5 (OCH$_2$***C***H$_3$), 12.3 (C=C***C***H$_3$); ^{19}F NMR (376 MHz, *chloroform-d*) δ -121.88 (dt, *J* = 55.8, 13.6 Hz); HRMS (ESI, m/z) calculated for $C_{10}H_{13}F_2NNaO_3$ (M Na)$^+$ 256.0761, found 256.0775; IR : v_{max}/cm^{-1} 2983, 1729, 1686, 1631, 1349, 1205, 1116, 1058, 980, 755, 589, 551.

Ethyl 1-(bicyclo[1.1.1]pentan-1-yl)-2-methyl-5-oxo-4,5-dihydro-1H-pyrrole-3-carboxylate (7r)

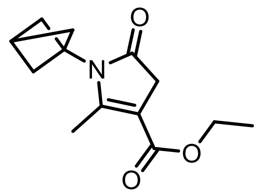

Synthesised from 2-methyl-3-ethoxycarbonyl-furan **5b** (0.450 mmol, 116 mg) and bicyclo[1.1.1]pentan-1-amine hydrochloride (107 mg, 0.900 mmol, 2.00 eq.) according to general procedure **2.4** using KPi Buffer (24.0 mL), MeCN (6.00 mL), and aq. H_2O_2 solution (0.900 mmol in 4.00 mL H_2O). Purification via flash chromatography on silica gel (eluent: hexane/ EtOAc = 7:3); as a yellow solid (114 mg, 64% yield); Mp 54 – 56 °C; ^{1}H NMR (400 MHz, *chloroform-d*) δ 4.16 (q, *J* = 7.1 Hz, 2H; OC***H$_2$***CH$_3$), 3.19 (q, *J* = 2.4 Hz, 2H; NC(O)C***H$_2$***), 2.51 – 2.47 (m, 4H; C=CC***H$_3$***), 2.35 (s, 6H; (NC(C***H$_2$***)$_3$)), 1.27 (t, *J* = 7.1 Hz, 3H; OCH$_2$C***H$_3$***); ^{13}C NMR (101 MHz,

chloroform-d) δ 176.5 (N***C***(O)CH_2) , 164.5 (***C***O_2Et), 155.0 (C=***C***CH_3), 103.3 (=***C***(CO_2Et), 59.9 (O***C***H_2CH_3) , 54.5 (NC(***C***H_2)$_3$), 50.1, 37.4, 26.1, 14.6 (OCH_2***C***H_3), 13.7 (C=C***C***H_3); HRMS (ESI, m/z) calculated for $C_{13}H_{17}NNaO_3$ $(MNa)^+$ 258.1106, found 258.1108; IR : v_{max}/cm^{-1} 2922, 1684, 1624, 1376, 1341, 1190, 1018, 874, 787 610, 501.

Ethyl 2-methyl-5-oxo-4,5-dihydro-1H-pyrrole-3-carboxylate (7s)

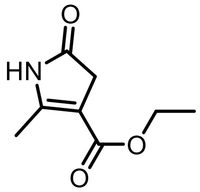

Synthesised from 2-methyl-3-ethoxycarbonyl-furan **5b** (0.900 mmol, 139 mg) and ammonium chloride (100 mg, 1.80 mmol) according to general procedure **2.3**. Purification via flash chromatography on silica gel (gradient eluent: hexane/ EtOAc = 1:1 to hexane/ EtOAc = 1:4); as an orange solid [5] (88 mg, 58% yield); ^{1}H NMR (400 MHz, *chloroform-d*) δ 7.89 (bs, 1H; N***H***), 4.19 (q, *J* = 7.1 Hz, 2H; OC***H₂***CH_3), 3.29 (q, *J* = 2.3 Hz, 2H; NC(O)C***H***$_2$), 2.36 (t, *J* = 2.3 Hz, 3H; C=CC***H***$_3$), 1.29 (t, *J* = 7.1 Hz, 3H; OCH_2C***H***$_3$); ^{13}C NMR (101 MHz, *chloroform-d*) δ 177.6 (N***C***(O)CH_2), 164.3 (***C***O_2Et), 151.3 (C=***C***CH_3), 104.6 (C=***C***(CO_2Et)), 60.0 (O***C***H_2CH_3), 37.5 (NC(O)C***H***$_2$), 14.6 (OCH_2***C***H_3), 13.6 (C=C***C***H_3).

Ethyl 2-methyl-5-oxo-1-phenethyl-4,5-dihydro-1H-pyrrole-3-carboxylate (7t)

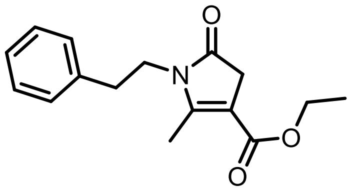

Synthesised from 2-methyl-3-ethoxycarbonyl-furan **5b** (0.500 mmol, 77 mg) and phenethyl amine (94.0 μL, 0.75 mmol, 1.50. eq.) according to general procedure **2.4** using KPi Buffer (26.0 mL), MeCN (6.00 mL), and aq. H_2O_2 solution (1.00 mmol in 4.00 mL H_2O). Purification via flash chromatography on silica gel (eluent: hexane/ EtOAc = 4:1); as a yellow solid (76 mg, 55% Yield); Mp 65 – 68 °C; ^{1}H NMR (400 MHz, *chloroform-d*) δ 7.32 – 7.27 (m, 2H), 7.26 – 7.21 (m, 1H), 7.18 – 7.15 (m, 2H), 4.17 (q, *J* = 7.1 Hz, 3H), 3.76 – 3.67 (m, 2H), 3.26 (q, *J* = 2.3 Hz, 2H), 2.86 (t, *J* = 7.4 Hz, 3H), 2.18 (t, *J* = 2.4 Hz, 4H), 1.28 (t, *J* = 7.1 Hz, 3H; OCH_2C***H***$_3$); ^{13}C NMR (101 MHz, *chloroform-d*) δ 176.1, 164.4, 154.2, 138.1, 129.0, 128.9, 127.0, 103.4, 59.9, 42.1, 36.7, 35.4, 14.6, 12.1; HRMS (ESI, m/z) calculated for $C_{16}H_{19}NNaO_3$

(M Na)$^+$ 296.1257, found 296.1264; IR : ν_{max}/cm^{-1} 2981, 1723, 1690, 1627, 1395, 1220, 1060, 963, 700, 551.

Ethyl 2-methyl-1-((5-methylfuran-2-yl)methyl)-5-oxo-4,5-dihydro-1H-pyrrole-3-carboxylate (7u)

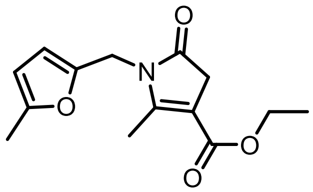

Synthesised from 2-methyl-3-ethoxycarbonyl-furan **5b** (0.750 mmol, 116 mg) and (5-methylfuran-2-yl)methanamine hydrochloride (182 mg, 1.50 mmol, 2.00 eq.) according to a modified version of general procedure **2.4** using KPi Buffer (40.0 mL), MeCN (10.0 mL), and aq. H_2O_2 solution (1.13 mmol in 4.00 mL H_2O). The difference compared general procedure **2.4** were that the amine and H_2O_2 solution were added at same rate (both 0.5 mL h^{-1}) and that a small amount of (5-methylfuran-2-yl)methanamine hydrochloride (9.1 mg, 0.075 mmol, 0.1 eq.) was added prior to peroxide addition). Purification via flash chromatography on silica gel (gradient eluent: hexane/EtOAc = 1:4 to hexane/EtOAc = 1:1); as an orange, solid (52 mg, 26% Yield); Mp 82 – 84°C; ^{1}H NMR (400 MHz, *chloroform-d*) δ 6.12 (d, *J* = 3.1 Hz, 1H), 5.88 – 5.85 (m, 1H), 4.63 (s, 2H), 4.18 (q, *J* = 7.1 Hz, 2H; OC***H***$_2$CH$_3$), 3.27 (q, *J* = 2.4 Hz, 2H), 2.52 (t, *J* = 2.4 Hz, 3H), 2.23 (s, 2H), 1.28 (t, *J* = 7.1 Hz, 3H); ^{13}C NMR (101 MHz, *chloroform-d*) δ 175.7 (N***C***O), 164.4 (***C***O$_2$Et), 154.0, 152.3, 147.7, 109.3, 106.5, 103.7, 59.9, 36.8, 36.7, 14.6, 13.7, 12.6; HRMS (ESI, m/z) calculated for $C_{14}H_{17}NNaO_4$ (M Na)$^+$ 286.1055, found 286.1050; IR : ν_{max}/cm^{-1} 1735, 1709, 1676, 1625, 1400, 1332, 1183, 1056, 965, 781, 724, 574.

Ethyl 2-methyl-5-oxo-1-(pyridin-2-ylmethyl)-4,5-dihydro-1H-pyrrole-3-carboxylate (7v)

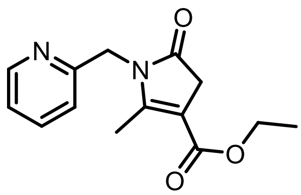

Synthesised from 2-methyl-3-ethoxycarbonyl-furan **5b** (0.750 mmol, 116 mg) and pyridin-2-ylmethanamine hydrochloride (200 mg, 1.50 mmol, 2.00 eq.) according to general procedure **2.4** using KPi Buffer (40.0 mL), MeCN (10.0 mL), and aq. H_2O_2 solution (1.50 mmol in 4.00 mL H_2O). Purification via flash chromatography on silica gel (gradient eluent: hexane/EtOAc = 2:3 to EtOAc); as a

yellow solid (113 mg, 57% yield); Mp 58 – 61°C; ^{1}H NMR (400 MHz, *chloroform-d*) δ 8.54 – 8.51 (m, 1H; NC***H***), 7.64 (td, *J* = 7.7, 1.8 Hz, 1H; NCHC***H***), 7.21 – 7.17 (m, 2H; NCHCHC***H***), 4.86 (s, 2H; NC$\boldsymbol{H_2}$), 4.18 (q, *J* = 7.1 Hz, 2H; OC$\boldsymbol{H_2}$$CH_3$), 3.36 (q, *J* = 2.4 Hz, 2H; NCOC$\boldsymbol{H_2}$), 2.40 (t, *J* = 2.4 Hz, 3H; NCOC$\boldsymbol{H_2}$), 1.28 (t, *J* = 7.1 Hz, 3H; $OCH_2C$$\boldsymbol{H_3}$); ^{13}C NMR (101 MHz, *chloroform-d*) δ 176.2 (N***C***O), 164.4 (***C***O_2Et), 156.1 (NCH_2***C***), 154.4 (=***C***CH_3), 149.7 (N***C***H), 137.1 (NCHCH***C***H), 122.8 (NCH***C***H), 121.7 (NCH***C***H), 103.9 (C=***C***(CO_2Et)), 59.9 (O***C***H_2CH_3), 45.5 (N***C***H_2), 36.8 (NCO***C***H_2), 14.6 (OCH_2***C***H_3), 12.8 (C=C***C***H_3); HRMS (ESI, m/z) calculated for $C_{14}H_{16}N_2NaO_3$ (M Na)$^+$ 283.1059, found 283.1059; IR : v_{max}/cm^{-1} 2960, 1726, 1682, 1679, 1590 1456, 1391, 1226, 1147, 1057, 820, 752, 717, 627.

Ethyl 2-methyl-1-neopentyl-5-oxo-4,5-dihydro-1H-pyrrole-3-carboxylate (7w)

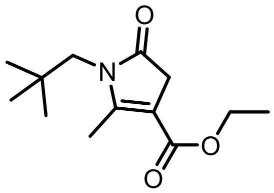

Synthesised from 2-methyl-3-ethoxycarbonyl-furan **5b** (0.600 mmol, 139 mg) and neopentyl amine (42.0 mg, 0.480 mmol, 0.800 eq.) according to general procedure **2.5** using KPi Buffer (32.0 mL), MeCN (8.00 mL), and aq. H_2O_2 solution (1.20 mmol in 4.00 mL H_2O). Purification via flash chromatography on silica gel (hexane/ EtOAc = 4:1); As a yellow oil (57 mg; 45% yield); ^{1}H NMR (400 MHz, *chloroform-d*) δ 4.17 (q, *J* = 7.2 Hz, 2H), 3.33 (s, 2H), 3.26 (q, *J* = 2.3 Hz, 2H; NC(O)C$\boldsymbol{H_2}$), 2.43 (t, *J* = 2.3 Hz, 3H; C=CC$\boldsymbol{H_3}$), 1.27 (t, *J* = 7.2 Hz, 3H), 0.93 (s, 9H); ^{13}C NMR (101 MHz, *chloroform-d*) δ 177.1 (N***C***(O)CH_2), 164.5 (***C***O_2Et), 155.5 (C=***C***CH_3), 103.5 (C=***C***(CO_2Et)), 59.8 (O***C***H_2CH_3), 51.0 (N***C***H_2), 36.6 (NC(O)***C***H_2), 34.2 (***C***$(CH_3)_3$), 28.5 (C(***C***$H_3)_3$), 14.6 (OCH_2***C***H_3), 13.4 (C=C***C***H_3); HRMS (ESI, m/z) calculated for $C_{13}H_{21}NNaO_3$ (M Na)$^+$ 264.1419, found 264.1419; IR: v_{max}/cm^{-1} 2960, 1729, 1690, 1623, 1478, 1388, 1343, 1217, 1147, 1057, 889, 726, 578.

Ethyl 1-(2-methoxy-2-oxoethyl)-2-methyl-5-oxo-4,5-dihydro-1H-pyrrole-3-carboxylate (7x)

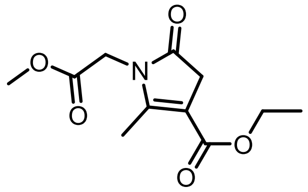

Synthesised from 2-methyl-3-ethoxycarbonyl-furan **5b** (0.750 mmol, 116 mg) and glycine methyl ester hydrochloride (187 mg, 1.50 mmol, 2.00 eq.) according to general procedure **2.4** using KPi Buffer (40.0 mL), MeCN (10.0 mL), and aq. H_2O_2 solution (1.50 mmol in 4.00 mL H_2O). Purification via flash chromatography on silica gel (gradient eluent: hexane/EtOAc = 3:2 to hexane/ EtOAc = 1:1); as a yellow solid (138 mg, 75% yield); Mp 75 – 77 °C; ^{1}H NMR (400 MHz, *chloroform-d*) δ 4.31 (s, 2H; NC***H₂***), 4.20 (q, *J* = 6.9 Hz, 2H; OC$\boldsymbol{H_2}$$CH_3$), 3.76 (s, 3H; OC$\boldsymbol{H_3}$), 3.33 (q, *J* = 2.3 Hz, 2H; NC(O)C$\boldsymbol{H_2}$), 2.36 (t, *J* = 2.3 Hz, 3H; C=CC$\boldsymbol{H_3}$), 1.29 (t, *J* = 7.3 Hz, 3H; $OCH_2C$$\boldsymbol{H_3}$); ^{13}C NMR (101 MHz, *chloroform-d*) δ 175.8 (N***C***O), 168.5 (***C***O_2Me), 164.2 (***C***O_2Et), 152.8 (C=***C***CH_3), 104.3 (C=***C***(CO_2Et)), 60.1 (O***C***H_2CH_3), 52.9 (O***C***H_3), 41.1 (N***C***H_2), 36.5 (NC(O)***C***H_2), 14.6 (OCH_2***C***H_3), 12.3 (C=C***C***H_3); HRMS (ESI, m/z) calculated for $C_{11}H_{15}NNaO_5$ (M Na)$^+$ 264.0842, found 264.0847; IR : v_{max}/cm^{-1} 2981, 1730, 1682, 1626, 1396, 1340, 1216, 1058, 982, 756, 591.

2-(4-(Ethoxycarbonyl)-5-methyl-2-oxo-2,3-dihydro-1H-pyrrol-1-yl)ethane-1-sulfonic acid (7y)

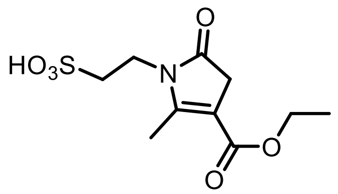

Synthesised from 2-methyl-3-ethoxycarbonyl-furan **5b** (0.750 mmol, 116 mg) and taurine hydrochloride (164 mg, 1.31 mmol, 1.75 eq.) according to general procedure **2.4** using KPi Buffer (40.0 mL), MeCN (10.0 mL), and aq. H_2O_2 solution (1.31 mmol in 4.00 mL H_2O). Additionally, using the following, alternative, extraction method. The reaction was extracted with $CHCl_3$/IPA (3:1, 30 mL) and the organic layer discarded. The aqueous phase was acidified with dropwise addition of HCl (aq) (pH ~ 1) then extracted thrice with $CHCl_3$/IPA (3:1, 3 x 30 mL). The combined organic phase was washed with saturated brine (50 mL), dried over

$MgSO_4$, filtered, and the solvent removed *in vacuo* to afford a yellow oil. Purification via flash chromatography on silica gel (gradient eluent: EtOAc/ MeOH = 4:1 to EtOAc/ MeOH = 7:3); as a dark yellow powder (61 mg, 22% yield); ^{1}H NMR (400 MHz, *dmso-d$_6$*) δ 4.09 (q, *J* = 7.1 Hz, 2H; $OC\boldsymbol{H_2}CH_3$), 3.71 (t, *J* = 7.1 Hz, 2H; $NC\boldsymbol{H_2}$), 3.14 (q, *J* = 2.3 Hz, 2H; $NC(O)C\boldsymbol{H_2}$), 2.65 (t, *J* = 7.1 Hz, 2H; $NCH_2C\boldsymbol{H_2}$), 2.44 (t, *J* = 2.3 Hz, 3H; $C{=}CC\boldsymbol{H_3}$), 1.20 (t, *J* = 7.1 Hz, 3H; $OCH_2C\boldsymbol{H_3}$); ^{13}C NMR (101 MHz, *dmso-d$_6$*) δ 175.1 ($N\boldsymbol{C}O$), 163.7 ($\boldsymbol{C}O_2Et$), 155.4 ($C{=}\boldsymbol{C}CH_3$), 101.7 ($C{=}\boldsymbol{C}(CO_2Et)$), 59.1 ($O\boldsymbol{C}H_2CH_3$), 49.1 ($NCH_2\boldsymbol{C}H_2$), 36.8 ($N\boldsymbol{C}H_2$), 35.9 ($NC(O)\boldsymbol{C}H_2$), 14.4 ($OCH_2\boldsymbol{C}H_3$), 12.1 ($C{=}C\boldsymbol{C}H_3$); HRMS (ESI, m/z) calculated for $C_{10}H_{14}O_6S$ $(M - H)^-$ 276.0547, found 276.0540; IR (film) : ν_{max}/cm^{-1} 3483, 2981, 1685, 1632, 1327, 1273, 1191, 1063, 565.

Ethyl 2-methyl-5-oxo-1-ureido-4,5-dihydro-1H-pyrrole-3-carboxylate (7z) [6]

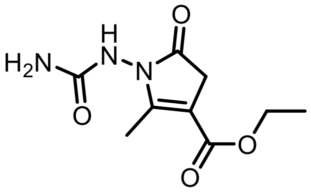

Synthesised from 2-methyl-3-ethoxycarbonyl-furan **5b** (0.600 mmol, 93 mg) and semicarbazide hydrochloride (133 mg, 1.20 mmol, 2.00 eq.) according to general procedure **2.5**, using KPi Buffer (24.0 mL), MeCN (6.00 mL), and aq. H_2O_2 solution (1.20 mmol in 4.00 mL H_2O). No further purification required; as a beige solid (69 mg, 50% yield); ^{1}H NMR (400 MHz, *dmso-d$_6$*) δ 8.55 (s, 1H), 6.34 (s, 1H), 4.12 (q, *J* = 6.4 Hz, 2H), 3.32 (d, *J* = 9.3 Hz, 1H; $NC(O)CH\boldsymbol{H}$), 3.22 – 3.15 (m, 1H; $NC(O)C\boldsymbol{H}H$), 2.21 (s, 3H; $C{=}CC\boldsymbol{H_3}$), 1.22 (t, *J* = 7.1 Hz, 3H; $OCH_2C\boldsymbol{H_3}$); ^{13}C NMR (101 MHz, *dmso-d$_6$*) δ 173.6 ($N\boldsymbol{C}(O)CH_2$), 163.5 ($\boldsymbol{C}O_2Et$), 59.3 ($O\boldsymbol{C}H_2CH_3$), 34.3 ($NC(O)\boldsymbol{C}H_2$), 14.4 ($OCH_2\boldsymbol{C}H_3$), 11.4 ($C{=}C\boldsymbol{C}H_3$); HRMS (ESI, m/z) calculated for $C_9H_{13}N_3NaO_4$ $(M\ Na)^+$ 250.0804, found 250.0796.

Ethyl 1-(3-amino-3-oxopropyl)-2-methyl-5-oxo-4,5-dihydro-1H-pyrrole-3-carboxylate (7za)

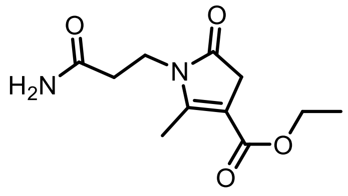

Synthesised from 2-methyl-3-ethoxycarbonyl-furan **5b** (0.750 mmol, 116 mg) and 3-aminopropanamide hydrochloride (187 mg, 1.50 mmol, 2.00 eq.) according to general procedure **2.4** using KPi Buffer (40.0 mL), MeCN (10.0 mL), and aq. H_2O_2 solution (1.50 mmol in 4.00 mL H_2O). Purification via flash chromatography on silica gel (eluent: DCM/MeOH = 19:1); as a white powder (77 mg, 42% yield); ^{1}H NMR (400 MHz, *dmso-d$_6$*) δ 7.41 (s, 1H; N***H***H), 6.89 (s, 1H; NH***H***), 4.08 (q, *J* = 6.8 Hz, 2H; OC***H$_2$***CH$_3$), 3.64 (t, *J* = 7.2 Hz, 1H; NC***H$_2$***), 3.17 (q, *J* = 2.3 Hz, 2H), 2.32 (t, *J* = 7.2 Hz, 1H; NCH$_2$C***H$_2$***), 1.20 (t, *J* = 6.8 Hz, 3H; OCH$_2$C***H$_3$***); ^{13}C NMR (101 MHz, *dmso-d$_6$*) δ 175.2, 171.9, 163.6, 154.9, 102.0, 59.1, 36.4, 36.0, 34.0, 14.4, 11.9; HRMS (ESI, m/z) calculated for $C_{11}H_{16}N_2NaO_4$ (M Na)$^+$ 263.1008, found 263.1006; HRMS (ESI, m/z) calculated for $C_{11}H_{16}N_2NaO_4$ (M Na)$^+$ 263.1008, found 263.1006; IR : v_{max}/cm^{-1} 3425, 2981, 1720, 1263, 1098, 728.

Ethyl 1-(but-3-en-1-yl)-2-methyl-5-oxo-4,5-dihydro-1H-pyrrole-3-carboxylate (7zb)

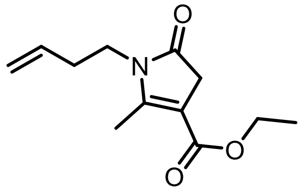

Synthesised from 2-methyl-3-ethoxycarbonyl-furan **5b** (0.750 mmol, 116 mg) and but-3-en-1-amine hydrochloride (161 mg, 1.50 mmol, 2.00 eq.) according to general procedure **2.4** using KPi Buffer (40.0 mL), MeCN (10.0 mL), and aq. H_2O_2 solution (1.50 mmol in 4.00 mL H_2O). Purification via flash chromatography on silica gel (gradient eluent: hexane/EtOAc = 4:1 to hexane/EtOAc = 7:3); as a yellow solid (66 mg, 39% yield); Mp 35 – 36 °C; ^{1}H NMR (400 MHz, *chloroform-d*) δ 5.72 (ddt, *J* = 17.3 10.3, 7.2 Hz, 1H; C***H***=CH$_2$), 5.08 – 5.04 (m, 1H; CH=CH***H***), 5.04 – 5.02 (m, 1H; CH=CH***H***), 4.16 (q, *J* = 7.1 Hz, 2H; OC***H$_2$***CH$_3$), 3.60 – 3.51 (m, 2H; C(O)NC***H$_2$***), 3.22 (q, *J* = 2.4 Hz, 2H; NCOC***H$_2$***), 2.42 (t, *J* = 2.4 Hz, 3H), 2.29 (qt, *J* = 7.2, 1.3 Hz, 2H;CH=CH$_2$C***H$_2$***), 1.26 (t, *J* = 7.1 Hz, 3H; OCH$_2$C***H$_3$***); ^{13}C NMR (101 MHz, *chloroform-d*) δ

176.1 (N***CO***), 164.3 (***C***O_2Et), 154.0 (=***C***CH_3), 134.2 (***C***H=CH_2), 117.9 (CH=***C***H_2), 103.5 (C=***C***(CO_2Et)), 59.8 (O***C***H_2CH_3), 39.6 (C(O)N***C***H_2), 36.7 (NCO***C***H_2), 33.6 (CH=CH_2***C***H_2), 14.5 (OCH_2***C***H_3), 12.5 (C=C***C***H_3); HRMS (ESI, m/z) calculated for $C_{12}H_{17}NNaO_3$ (M Na)$^+$ 246.1106, found 246.1101; IR : ν_{max}/cm^{-1} 2980, 1724, 1689, 1627, 1395, 1266, 1149, 1059, 756, 571.

Ethyl 1-(3-chloropropyl)-2-methyl-5-oxo-4,5-dihydro-1H-pyrrole-3-carboxylate (7zc)

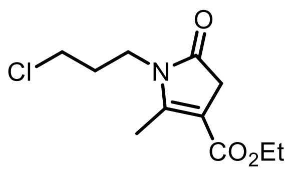

Synthesised from 2-methyl-3-ethoxycarbonyl-furan **5b** (0.750 mmol, 116 mg) and 3-chloropropylamine hydrochloride (195 mg, 1.50 mmol, 2.00 eq.) according to general procedure **2.4** using KPi Buffer (40.0 mL), MeCN (10.0 mL), and aq. H_2O_2 solution (1.50 mmol in 4.00 mL H_2O). Purification via flash chromatography on silica gel (eluent: hexane/EtOAc = 3:2); as a yellow solid (115 mg, 62% yield); Mp 72 – 74°C; ^{1}H NMR (400 MHz, *chloroform-d*) δ 4.19 (q, *J* = 7.1 Hz, 2H; OC***H_2***CH_3), 3.71 – 3.63 (m, 2H; C***H_2***N), 3.55 (t, *J* = 6.2 Hz, 2H; ClC***H_2***), 3.25 (q, *J* = 2.4 Hz, 2H; NCOC***H_2***), 2.47 (t, *J* = 2.4 Hz, 3H; C=CC***H_3***), 2.11 – 2.00 (m, 2H; (ClCH_2C***H_2***), 1.28 (t, *J* = 7.1 Hz, 3H; OCH_2C***H_3***); ^{13}C NMR (101 MHz, *chloroform-d*) δ 176.3 (N***C***O), 164.3 (***CO2Et***), 153.8 (=***C***CH_3), 103.9 (C=***C***(CO_2Et)), 60.0 (O***C***H2CH3), 42.1 (Cl***C***H_2), 37.9 (***C***H_2N), 36.7 (NCO***C***H_2), 31.8 (ClCH_2***C***H_2), 14.6 (OCH_2***C***H_3), 12.4 (C=C***C***H_3); HRMS (ESI, m/z) calculated for $C_{11}H_{16}ClNNaO_3$ (M Na)$^+$ 268.0716, found 268.0705; IR : ν_{max}/cm^{-1} 2985, 1717, 1679, 1627, 1392, 1343, 1221, 1158, 1057, 855, 776, 648, 582.

Ethyl 2-methyl-1-(2-morpholinoethyl)-5-oxo-4,5-dihydro-1H-pyrrole-3-carboxylate (7zd)

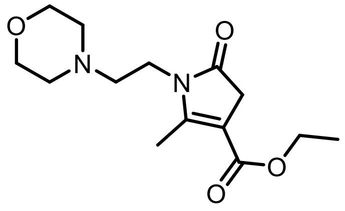

Synthesised from 2-methyl-3-ethoxycarbonyl-furan **5b** (0.750 mmol, 116 mg) and 2-morpholinoethan-1-amine hydrochloride (188 mg, 1.50 mmol, 2.00 eq.) according to general procedure **2.4** using KPi Buffer (40.0 mL), MeCN (10.0 mL), and aq. H_2O_2 solution (1.50 mmol in 4.00 mL H_2O). Purification via flash chromatography on silica gel (eluent: hexane/ EtOAc = 1:4 to EtOAc/MeOH = 19:1); as a brown oil (130 mg, 62% yield); ^{1}H NMR (400 MHz, chloroform-*d*) δ 4.18 (q, *J* = 7.2 Hz, 2H; OC***H₂***CH₃), 3.67 (t, *J* = 4.6 Hz, 4H; C***H₂***OC***H₂***), 3.62 (t, *J* = 6.7 Hz, 2H; C(O)NC***H₂***), 3.24 (q, *J* = 2.4 Hz, 2H; NCOC***H₂***), 2.54 – 2.47 (m, 6H), 2.46 (t, *J* = 2.4 Hz, 3H; C=CC***H₃***), 1.28 (t, *J* = 7.2 Hz, 3H; OCH₂C***H₃***); ^{13}C NMR (101 MHz, *chloroform-d*) δ 176.2 (N***C***O), 164.4 (***C***O₂Et), 154.2 (=***C***CH₃), 103.6 (C=***C***(CO₂Et)), 67.0 (C***H₂***OC***H₂***), 57.3 (C(O)NCH₂***C***H2), 54.0, 37.6 (C(O)N***C***H₂), 36.7 (NCO***C***H₂), 14.6 (OCH₂***C***H₃), 12.4 (C=C***C***H₃); HRMS (ESI, m/z) calculated for $C_{14}H_{23}N_2NaO_4$ (M H)$^+$ 283.1658, found 283.1672; IR : v_{max}/cm^{-1} 2856, 1688, 1628, 1395, 1230, 1147, 1060, 756.

Ethyl 1-(2-(cyclohex-1-en-1-yl)ethyl)-2-methyl-5-oxo-4,5-dihydro-1H-pyrrole-3-carboxylate (7ze)

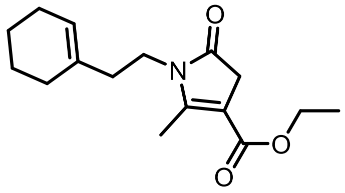

Synthesised from 2-methyl-3-ethoxycarbonyl-furan **5b** (0.750 mmol, 116 mg) and 2-(cyclohex-1-en-1-yl) ethan-1-amine hydrochloride (211 mg, 1.31 mmol, 1.75 eq.) according to general procedure **2.4** using KPi Buffer (40.0 mL), MeCN (10.0 mL), and aq. H_2O_2 solution (1.50 mmol in 4.00 mL H_2O). Purification via flash chromatography on silica gel (eluent: hexane/ EtOAc = 4:1 to hexane/EtOAc = 1:4); as a waxy white solid (52 mg, 25% yield); ^{1}H NMR (400 MHz, *chloroform-d*) δ 5.43 – 5.36 (m, 1H; C=C***H***), 4.17 (q, *J* = 7.1 Hz, 2H; OC***H₂***CH₃), 3.60

– 3.52 (m, 2H; NC***H₂***), 3.22 (q, *J* = 2.4 Hz, 2H; NCOC***H₂***), 2.42 (t, *J* = 2.4 Hz, 3H; C=CC***H₃***), 2.17 (t, *J* = 7.8 Hz, 2H; NCH_2C***H₂***), 2.01 – 1.90 (m, 4H; C***H₂***C=CHC***H₂***), 1.66 – 1.47 (m, 4H; C***H₂***C***H₂***), 1.29 (t, *J* =7.1 Hz 3H; OCH_2C***H₃***); ^{13}C NMR (101 MHz, *chloroform-d*) δ 176.0 (N***C***O), 164.5 (***C***O_2Et), 154.3 (=***C***CH_3), 134.1 (***C***=CH), 124.2 (C=***C***H), 103.3 (C=***C***(CO_2Et)), 59.8 (O***C***H_2CH_3), 39.1 (N***C***H_2), 37.5 (NCH_2***C***H_2), 36.7 (NCO***C***H_2), 28.4 (CH=C***C***H_2), 25.4 (C=CH***C***H_2), 22.9 (***C***H_2CH_2), 22.3 (CH_2***C***H_2), 14.6 (OCH_2***C***H_3), 12.4 (C=C***C***H_3); HRMS (ESI, m/z) calculated for $C_{16}H_{23}NNaO_3$ (M Na)$^+$ 300.1576, found 300.1576; IR : ν_{max}/cm^{-1} 2931, 1723, 1692, 1627, 1447, 1397, 1376, 1221, 1172, 1060, 755.

Ethyl 1-(3,4-dimethoxyphenethyl)-2-methyl-5-oxo-4,5-dihydro-1H-pyrrole-3-carboxylate (7zf)

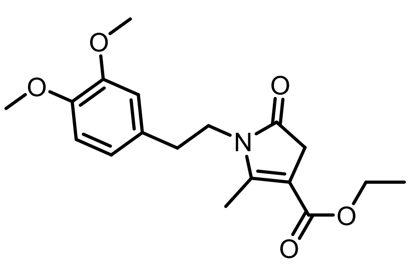

Synthesised from 2-methyl-3-ethoxycarbonyl-furan **5b** (0.750 mmol, 116 mg) and homoveratyl amine hydrochloride (244 mg, 1.13 mmol, 1.50 eq.) according to general procedure **2.4**, using KPi Buffer (40.0 mL), MeCN (10.0 mL), and aq. H_2O_2 solution (1.31 mmol in 4.00 mL H_2O). Purification via flash chromatography on silica gel (eluent: hexane/ EtOAc = 3:2); as a yellow solid (180 mg, 72% yield); (isolated as 10:1 mixture, with NH pyrrolinone **7s**); M.p. 70 – 73 °C; NMR data for **7zf** only: ^1H NMR (400 MHz, *chloroform-d*) δ 6.79 (d, *J* = 8.1 Hz, 1H; C=CH=C***H***=C(OMe)), 6.70 (dd, *J* = 8.1, 2.0 Hz, 1H; C=C***H***=CH=C(OMe)), 6.66 (d, *J* = 2.0 Hz, 1H; C=CH=CH=C(OMe)=C(OMe)=C***H***), 4.18 (q, *J* = 7.1 Hz, 2H; OC***H₂***CH_3), 3.86 (s, 3H; OC***H₃***), 3.85 (s, 3H; OC***H₃***), 3.70 (t, *J* = 7.4 Hz, 2H; NC***H₂***CH_2), 3.26 (q, *J* = 2.4 Hz, 2H; NC(O)C***H₂***), 2.81 (t, *J* = 7.4 Hz, 2H; NCH_2C***H₂***), 2.21 (t, *J* = 2.4 Hz, 3H; C=CC***H₃***), 1.35 – 1.21 (m, 3H; OCH_2C***H₃***); ^{13}C NMR (101 MHz, *chloroform-d*) δ 176.3 (N***C***O), 164.4 (***C***O_2Et), 154.3 (C=***C***CH_3), 149.2 (C=CH=CH=***C***(OMe)=C(OMe)=CH), 148.1 (C=CH=CH=C(OMe)=***C***(OMe)=CH), 130.6 (NCH_2CH_2***C***), 120.9 (C=***C***H=CH=C(OMe)),

112.1 (C=CH=CH=C(OMe)=C(OMe)=***C***H), 111.5 (C=CH=***C***H=C(OMe)), 103.4 (C=***C***(CO_2Et)), 59.9 (O***C***$H_2$$CH_3$), 56.0 (O***C***$H_3$), 42.2 (N***C***$H_2$$CH_2$), 36.7 (NC(O)***C***$H_2$), 34.9 ($NCH_2$***C***$H_2$), 14.6 ($OCH_2$***C***$H_3$), 12.3 (C=C***C***$H_3$); HRMS (ESI, m/z) calculated for $C_{18}H_{23}NNaO_5$ (M Na)$^+$ 356.1468, found 356.1466; IR : v_{max}/cm^{-1} 2829, 1721, 1690, 1617, 1515, 1398, 1218, 1139, 1031, 984, 753, 551.

Ethyl 1-(3-((((9H-fluoren-9-yl)methoxy)carbonyl)amino)propyl)-2-methyl-5-oxo-4,5-dihydro-1H-pyrrole-3-carboxylate (7zg)

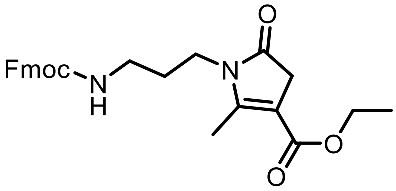

Synthesised from 2-methyl-3-ethoxycarbonyl-furan **5b** (0.750 mmol, 116 mg) and *N*-Fmoc-1,3-diaminopropane hydrochloride [7] (373 mg, 1.13 mmol, 1.50 eq.) in DMF (4.00 mL) according to general procedure **2.4**, using KPi Buffer (40.0 mL), MeCN (10.0 mL), and aq. H_2O_2 solution (1.13 mmol in 4.00 mL H_2O). Purification via flash chromatography on silica gel (gradient eluent: hexane/ EtOAc = 4:1 to hexane/EtOAc = 1:1); as a yellow solid (110 mg, 36% yield); Mp 146 – 147 °C; (decomp.); ^{1}H NMR (400 MHz, *chloroform-d*) δ 7.76 (d, *J* = 7.4 Hz, 2H; C***H***$_{Ar}$ Fmoc), 7.61 (d, *J* = 7.6 Hz, 2H; C***H***$_{Ar}$ Fmoc), 7.39 (t, *J* = 7.2 Hz, 2H; C***H***$_{Ar}$ Fmoc), 7.31 (td, *J* = 7.5, 1.3 Hz, 2H; C***H***$_{Ar}$ Fmoc), 5.62 (t, *J* = 6.6 Hz, 1H; N***H***), 4.37 (d, *J* = 7.2 Hz, 2H; C***H₂*** *Fmoc*), 4.29 – 4.14 (m, 3H; OC***H₂***CH_3 and C***H*** *Fmoc*), 3.60 (t, *J* = 6.5 Hz, 2H; NC***H₂***), 3.30 (q, *J* = 2.4 Hz, 2H; NC(O)C***H₂***), 3.18 (q, *J* = 6.4 Hz, 2H; NCH2CH2CH2), 2.46 – 2.40 (m, 3H; C=CC***H₃***), 1.77 – 1.70 (m, 2H; NCH_2C***H₂***), 1.29 (t, *J* = 7.1 Hz, 3H; OCH_2C***H₃***)); ^{13}C NMR (101 MHz, *chloroform-d*) δ 177.0 (N***C***O), 164.2 (***C***O_2Et), 156.6 (N***C***$O_2$$CH_2$*Fmoc*), 153.5 (C=***C***CH_3), 144.1 (***C***$_{Ar}$ Fmoc), 141.4 (***C***$_{Ar}$ Fmoc), 127.8 (***C***H_{Ar} Fmoc), 127. 2 (***C***H_{Ar} Fmoc), 125.2 (***C***H_{Ar} Fmoc), 120.1 (***C***H_{Ar} Fmoc), 104.3 (C=***C***(CO_2Et)), 66.9 (***C***H_2 *Fmoc*), 60.0 (O***C***$H_2$$CH_3$), 47.4 (***C***H *Fmoc*), 37.6 (NCH_2CH_2C***H₂***), 37.1 (N***C***H_2), 36.7 (NC(O)***C***H_2), 29.5 (NCH_2***C***H_2), 14.6 (OCH_2***C***H_3), 12.3 (C=C***C***H_3); HRMS (ESI, m/z)

calculated for $C_{26}H_{28}N_2NaO_5$ (M Na)$^+$ 471.1896, found 471.1895; IR (film) : v_{max}/cm^{-1} 3318, 2946, 1685, 1622, 1449, 1392, 1221, 1055, 755, 581.

Ethyl (R)-1-(1-methoxypropan-2-yl)-2-methyl-5-oxo-4,5-dihydro-1H-pyrrole-3-carboxylate (7zh)

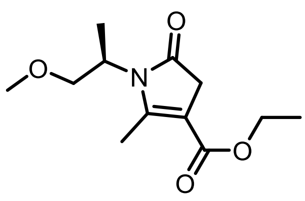

Synthesised from 2-methyl-3-ethoxycarbonyl-furan **5b** (0.600 mmol, 93.0 mg) and (R)-1-methoxypropan-2-amine hydrochloride (112 mg, 0.900 mmol, 1.50 eq) according to general procedure **2.5**; using KPi Buffer (24.0 mL), MeCN (6.00 mL), and aq. H_2O_2 solution (1.20 mmol in 4.00 mL H_2O). Purification via flash chromatography on silica gel (eluent: hexane/EtOAc = 1:1); as a yellow oil (69 mg, 38% yield); $[\alpha]_D^{20}$ = + 9.35 (c = 1.00 in chloroform); ^{1}H NMR (400 MHz, *chloroform-d*) δ 4.18 (q, *J* = 7.1 Hz, 2H; OC***H₂***CH₃), 4.02 – 3.95 (m, 1H; NC***H***(CH₃)CH₂), 3.89 (t, *J* = 9.2 Hz, 1H; NCH(CH₃)C***H***H), 3.47 (dd, *J* = 9.2, 5.0 Hz, 1H; NCH(CH₃)CH***H***), 3.31 (s, 3H; OC***H₃***), 3.22 (q, *J* = 2.3 Hz, 2H), 2.43 (t, *J* = 2.3 Hz, 3H), 1.40 (d, *J* = 6.9 Hz, 3H; NCH(C***H₃***)CH₂), 1.28 (t, *J* = 7.1 Hz, 3H; OCH₂C***H₃***); ^{13}C NMR (101 MHz, *chloroform-d*) δ 176.7 (N***C***(O)CH₂), 164.5 (***C***O₂Et), 155.3 (C=***C***CH₃), 103.6 (C=***C***(CO₂Et)), 73.3 (***C***H₂OCH₃), 59.8 (O***C***H₂CH₃), 59.1 (O***C***H₃), 49.9 (N***C***H(CH₃)CH₂), 37.3 (NC(O)***C***H₂), 15.3(NCH(***C***H₃)CH₂), 14.6 (OCH₂***C***H₃), 12.8 (C=C***C***H₃); HRMS (ESI, m/z) calculated for $C_{12}H_{19}NNaO_4$ (M Na)$^+$ 264.1212, found 264.1204; IR : v_{max}/cm^{-1} 2981, 1722, 1692, 1630, 1403, 1213, 1213, 1110, 1065, 757, 552.

Ethyl 1-(3-hydroxypropyl)-2-methyl-5-oxo-4,5-dihydro-1H-pyrrole-3-carboxylate (7zi)

Synthesised from 2-methyl-3-ethoxycarbonyl-furan **5b** (0.750 mmol, 116 mg) and 3-amino-1-propanol hydrochloride (157 mg, 1.5 mmol, 2.00 eq.) according to general procedure **2.4** using KPi Buffer (40.0 mL), MeCN (10.0 mL), and aq. H_2O_2 solution (1.50 mmol in 4.00 mL H_2O). Purification via flash chromatography on silica gel (gradient eluent: hexane/ EtOAc = 1:1 to

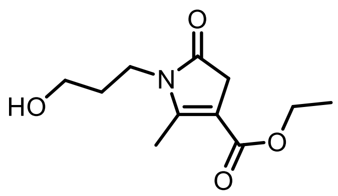

EtOAc); as a yellow oil (45 mg, 26% yield); ^{1}H NMR (400 MHz, *chloroform-d*) δ 4.16 (q, *J* = 7.2 Hz, 2H; OC***H***$_2$CH$_3$), 3.65 (t, *J* = 6.4 Hz, 2H; NC***H***$_2$CH$_2$), 3.53 (t, *J* = 5.7 Hz, 2H; NCH$_2$CH$_2$C***H***$_2$), 3.27 (q, *J* = 2.3 Hz, 2H; NC(O)C***H***$_2$), 2.42 (t, *J* = 2.3 Hz, 3H; C=CC***H***$_3$), 1.77 – 1.70 (m, 2H; NCH$_2$C***H***$_2$CH$_2$), 1.24 (d, *J* = 7.2 Hz, 3H; OCH$_2$C***H***$_3$); ^{13}C NMR (101 MHz, *chloroform-d*) δ 177.5 (N***C***(O)CH$_2$), 164.2 (***C***O$_2$Et), 153.7 (C=***C***CH$_3$), 104.4 (C=***C***(CO$_2$Et)), 60.0 (O***C***H$_2$CH$_3$), 58.4 (NCH$_2$CH$_2$C***H***$_2$), 36.7 (NC(O)***C***H$_2$), 36.4 (N***C***H$_2$CH$_2$CH$_2$), 31.9 (NCH$_2$***C***H$_2$CH$_2$), 14.5 (OCH$_2$***C***H$_3$), 12.2 (C=C***C***H$_3$); HRMS (ESI, m/z) calculated for $C_{11}H_{17}NNaO_4$ (M Na) $^{+}$ 250.1050, found 250.1053; IR: v_{max}/cm^{-1} 3417, 2997, 1689, 1488, 1346, 1230, 1061, 877, 755.

4) ^{1}H and ^{13}C spectra

NMR spectra for **7a**

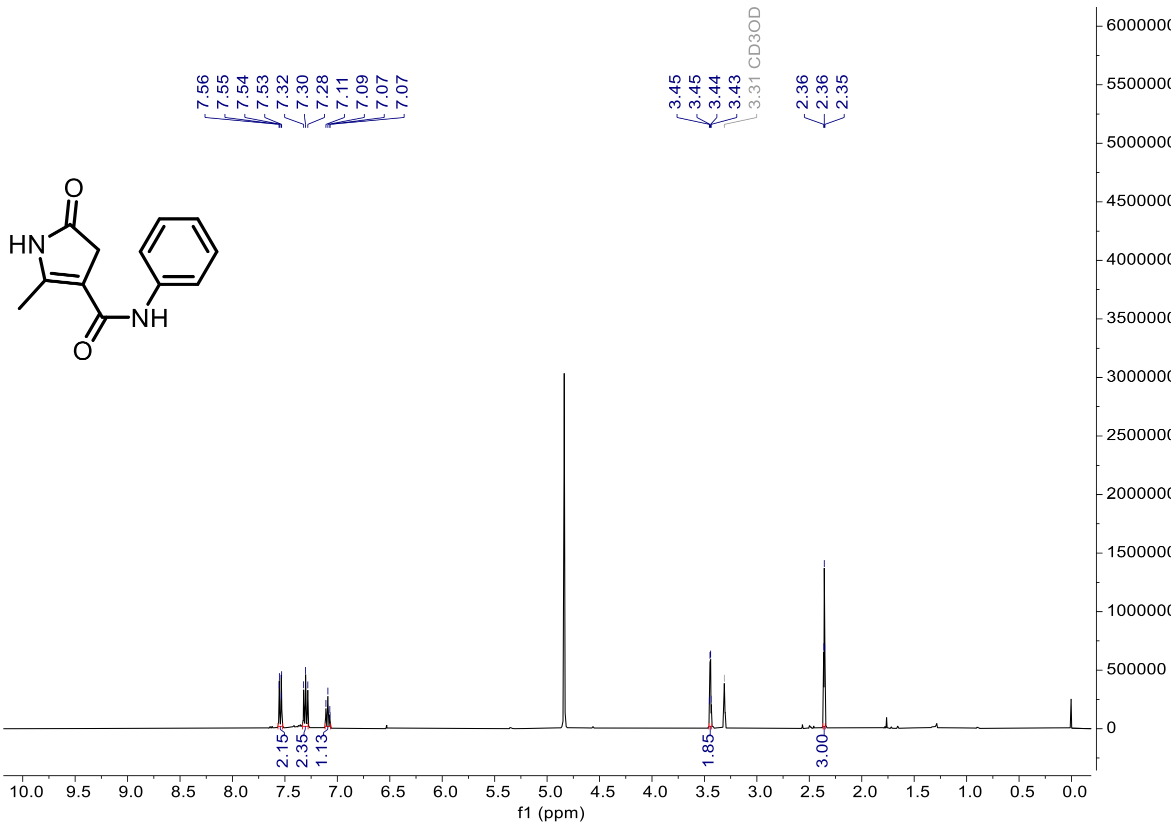

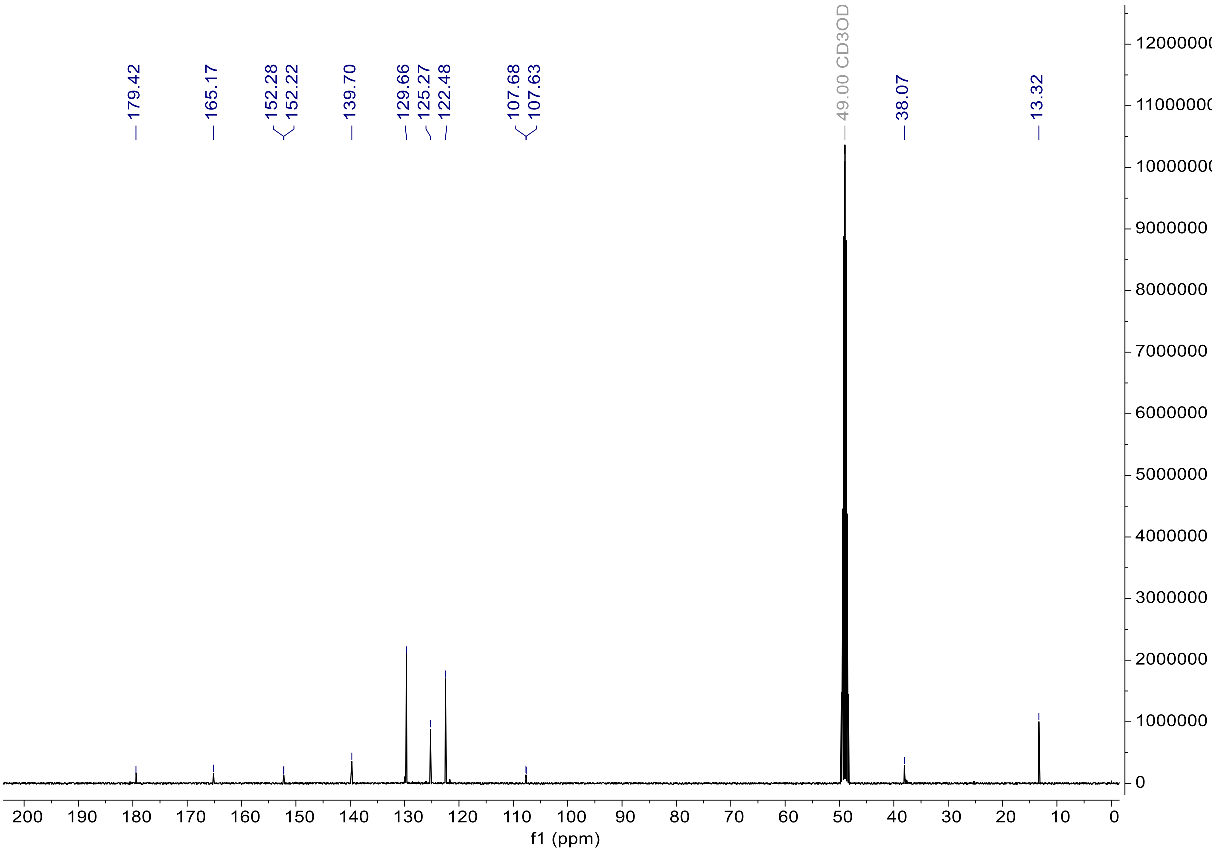

NMR spectra for **8**

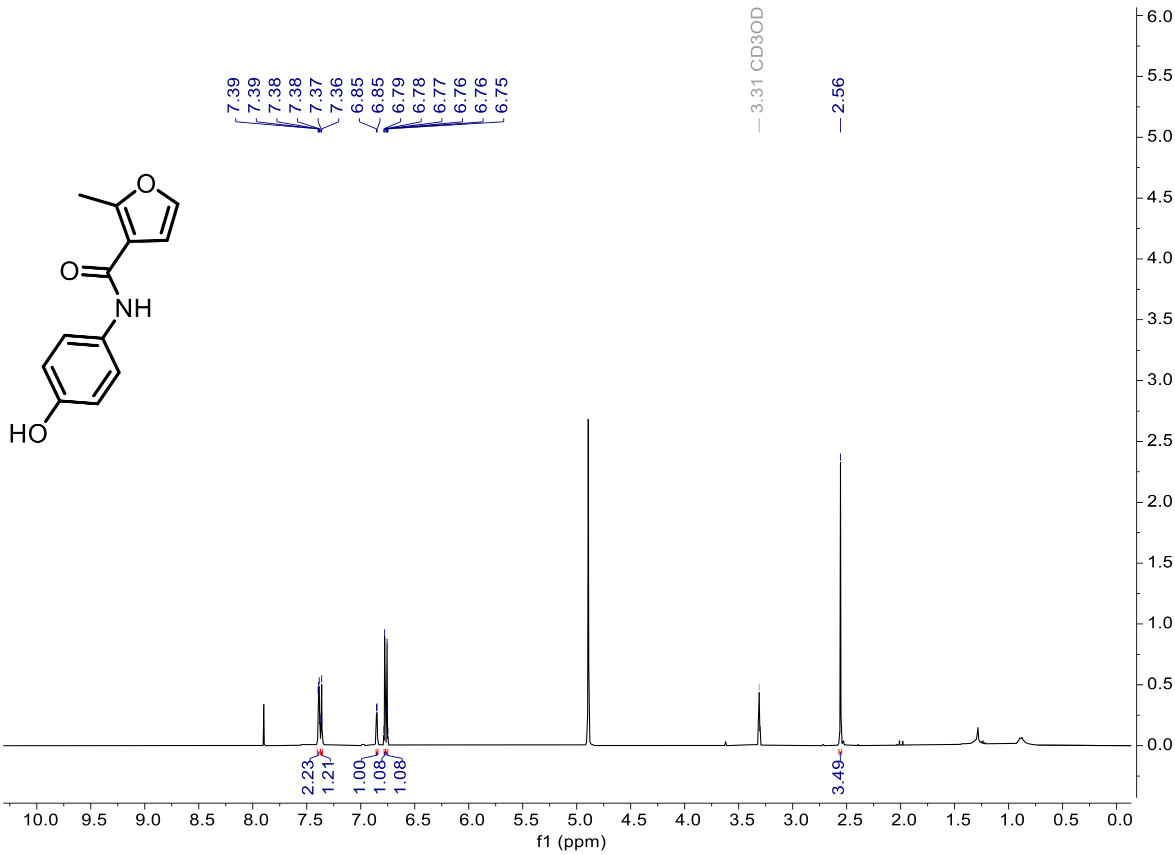

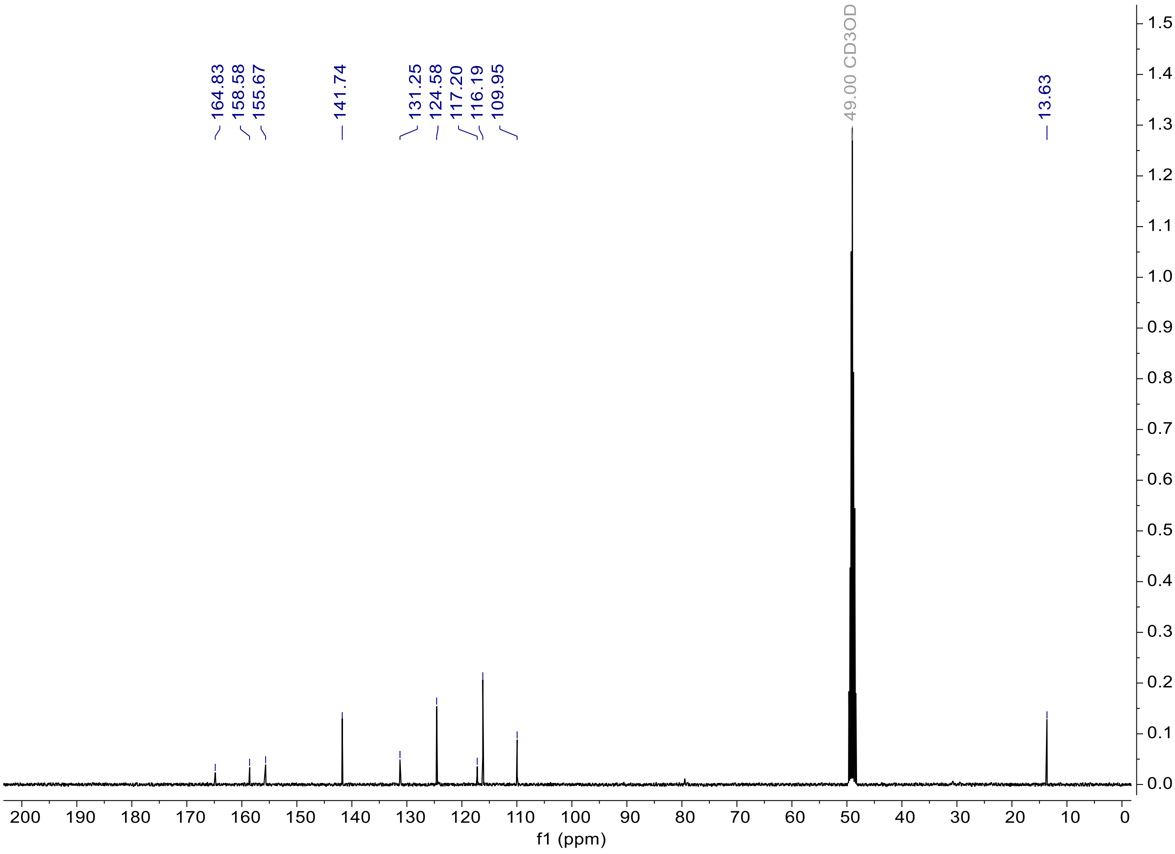

NMR spectra for **7b**

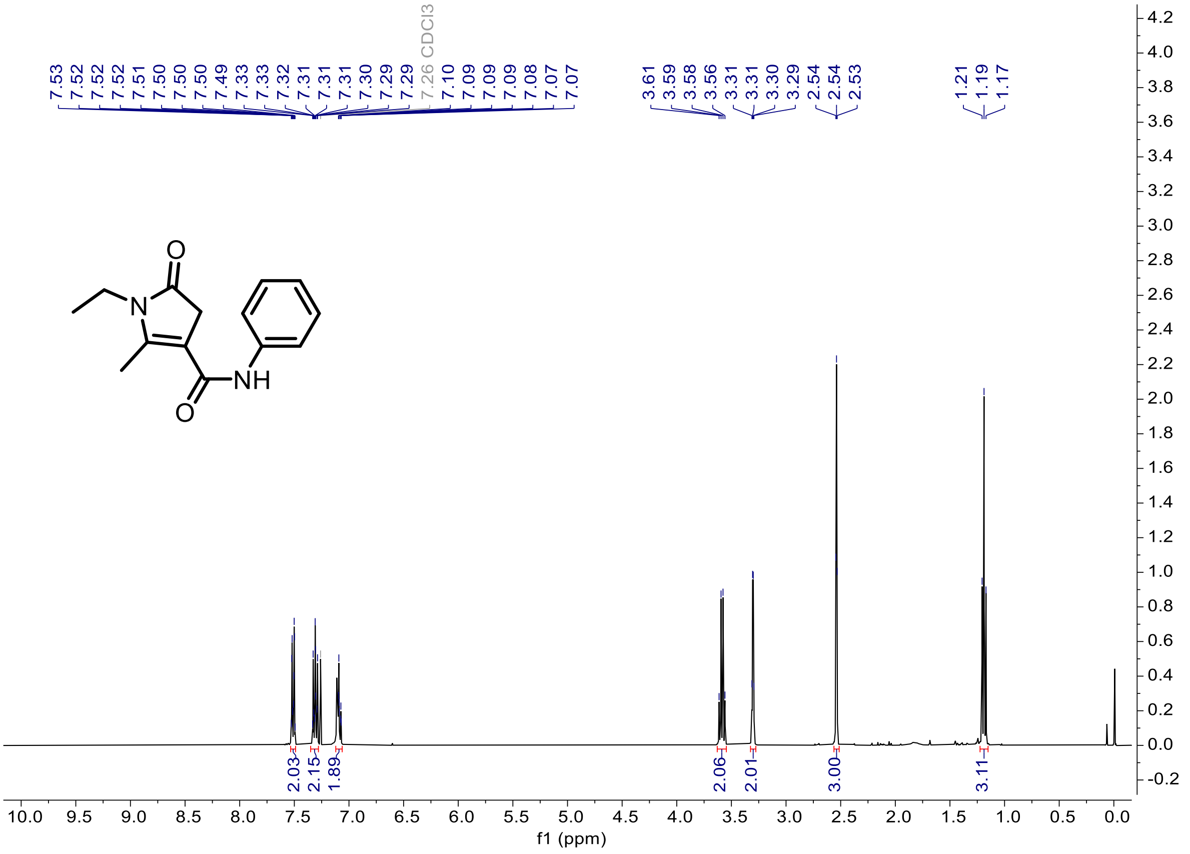

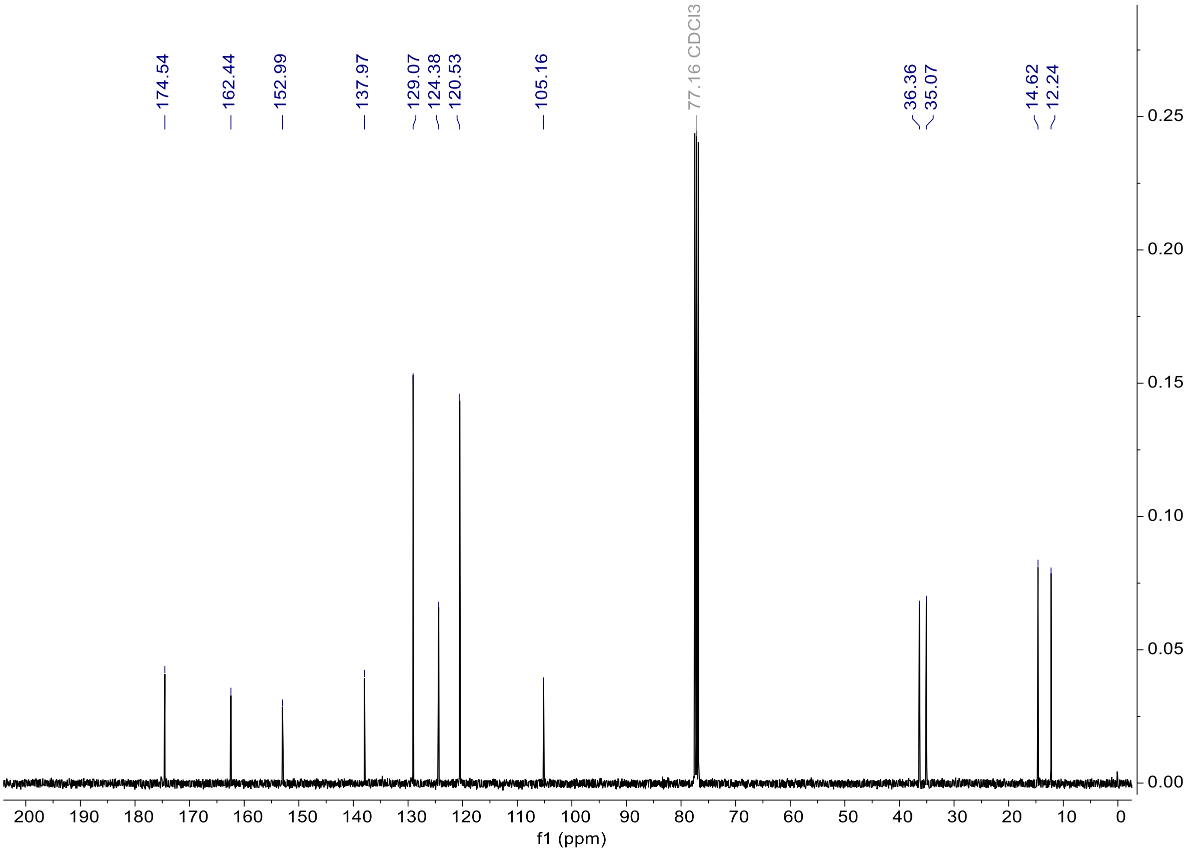

NMR spectra for **7c**

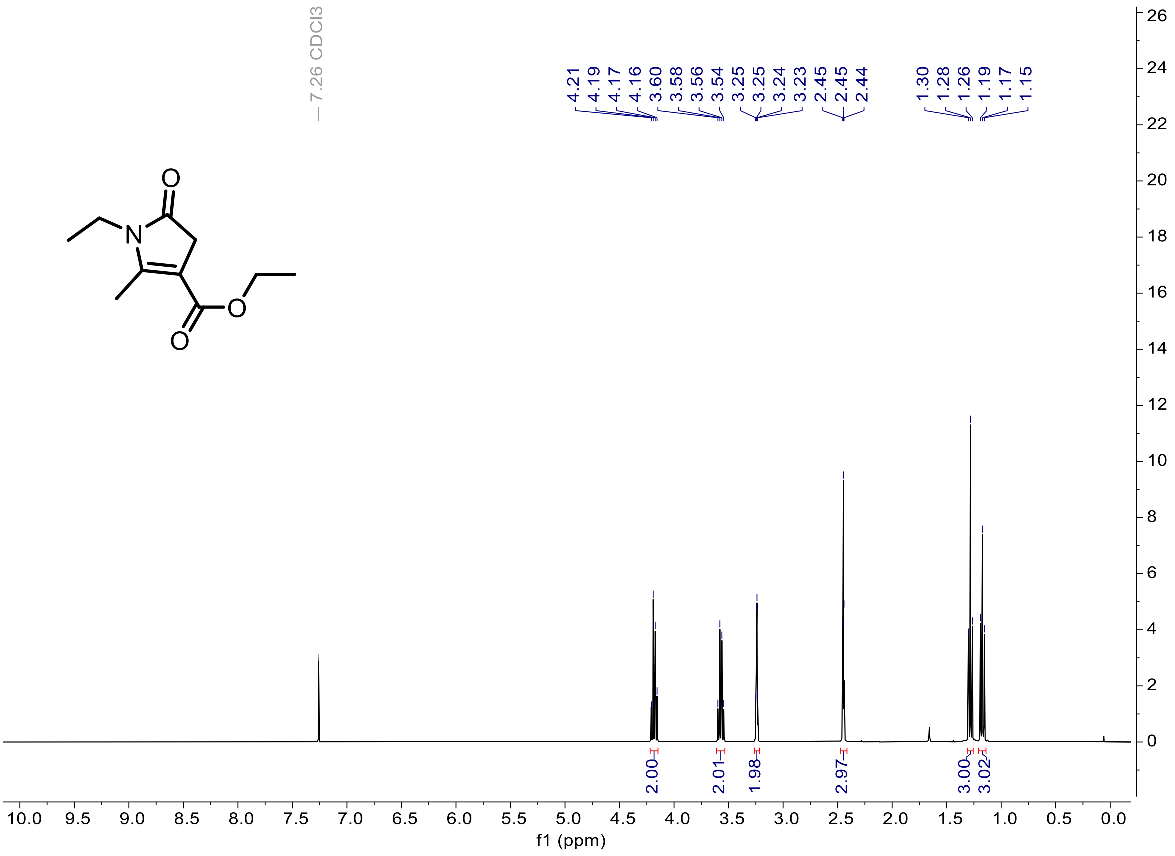

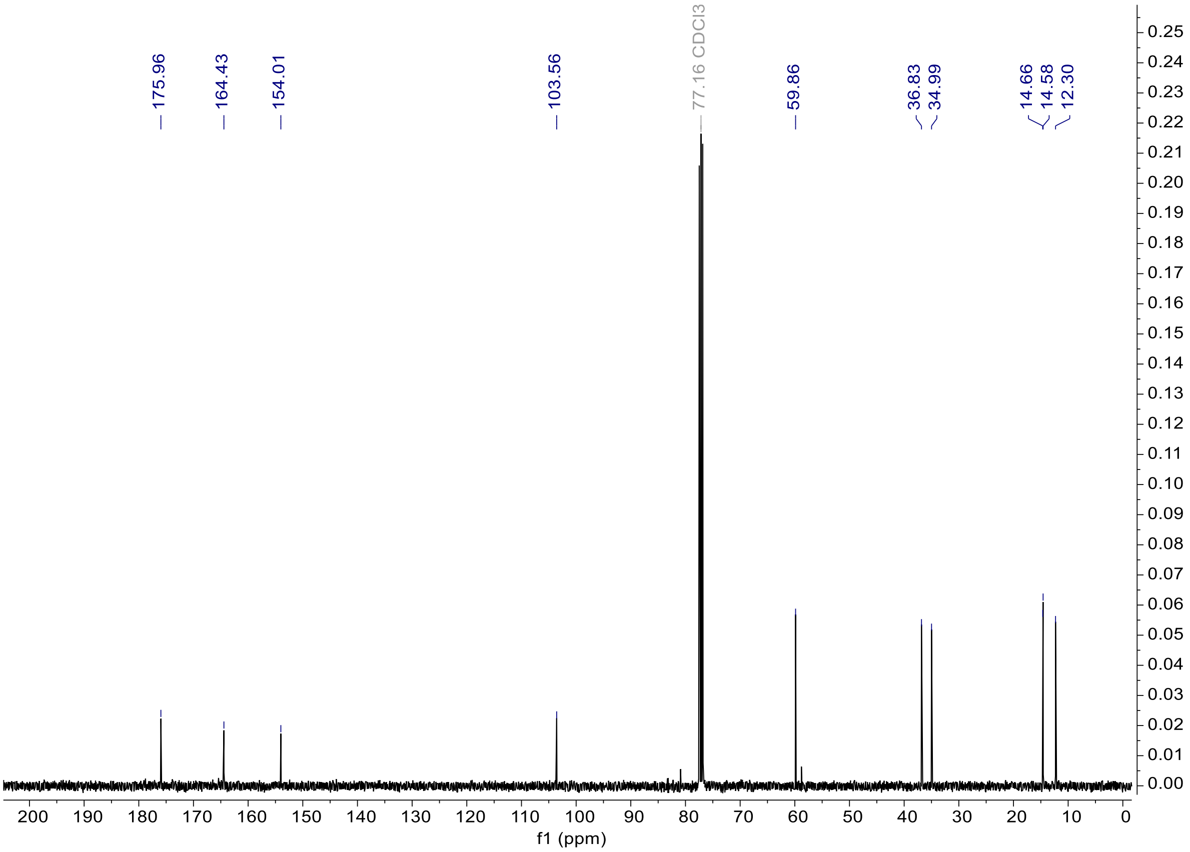

NMR spectra for **7d**

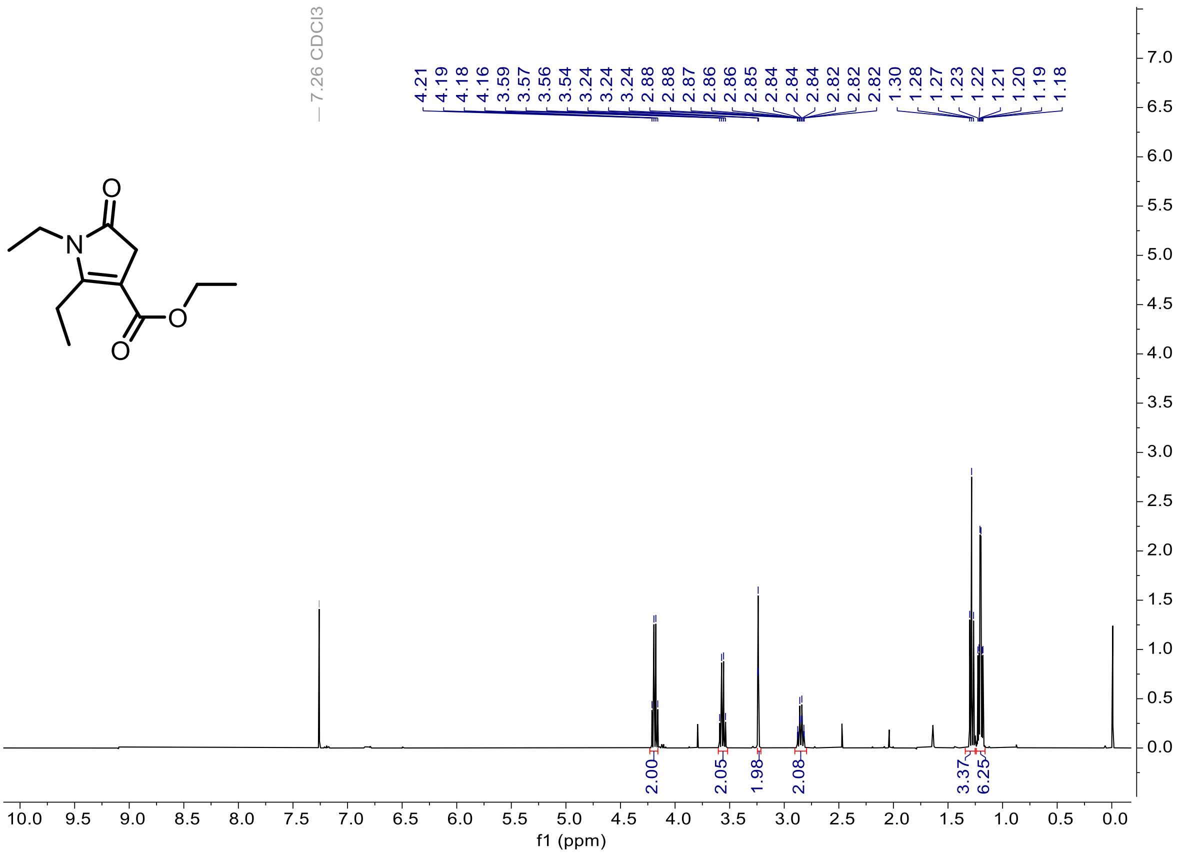

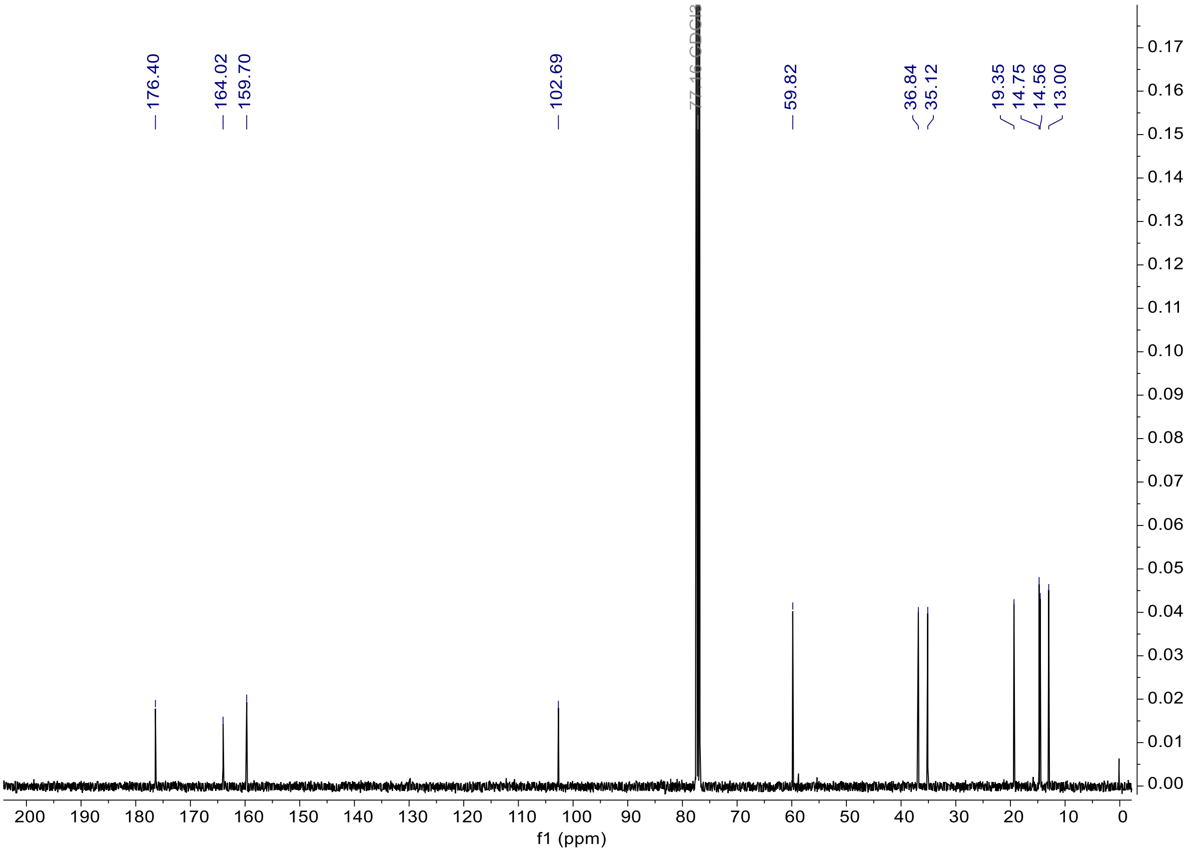

NMR spectra for **7e**

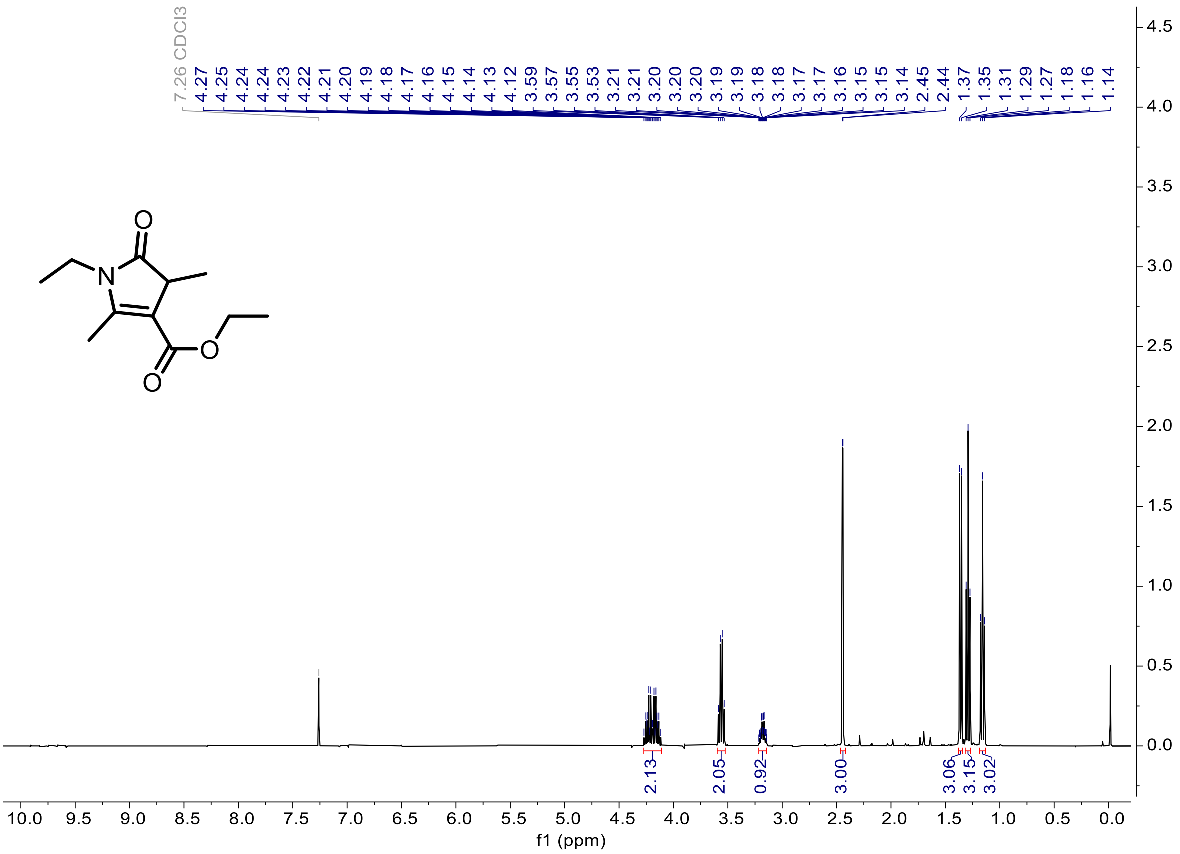

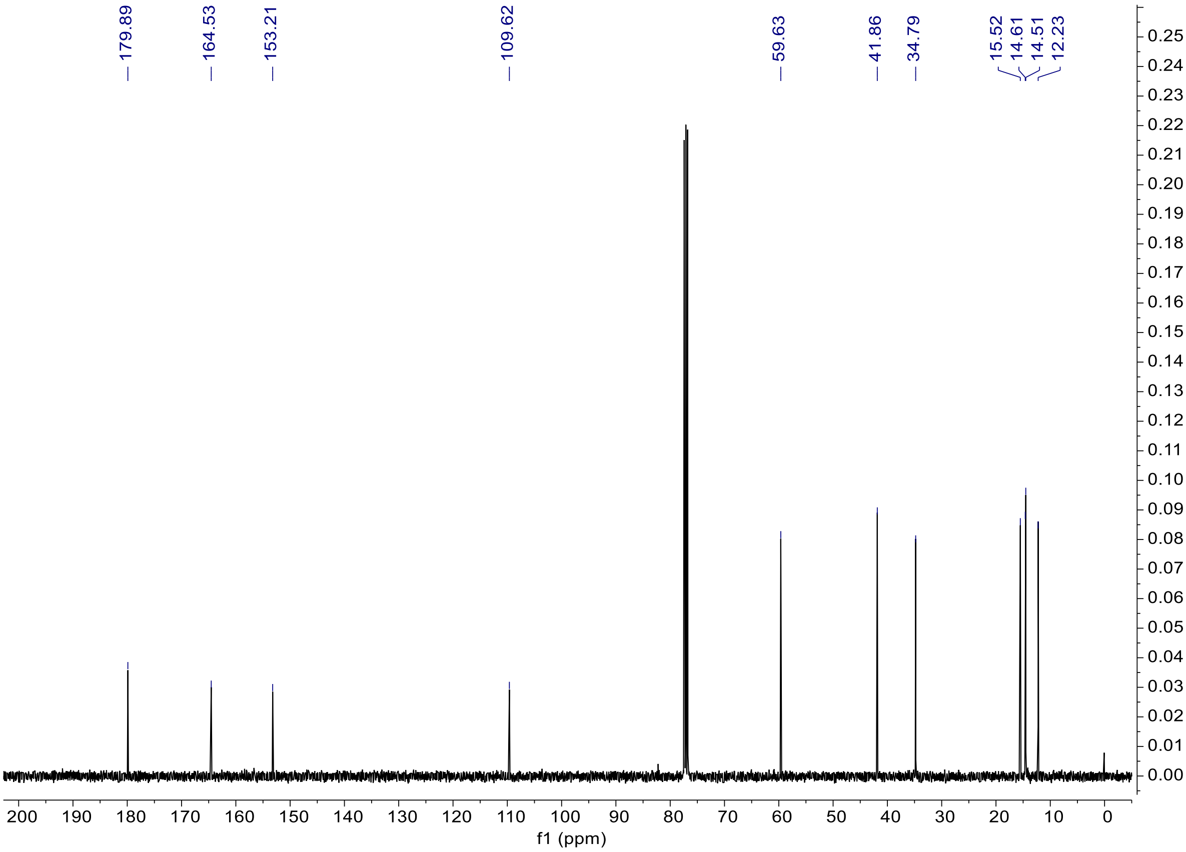

NMR spectra for **7f**

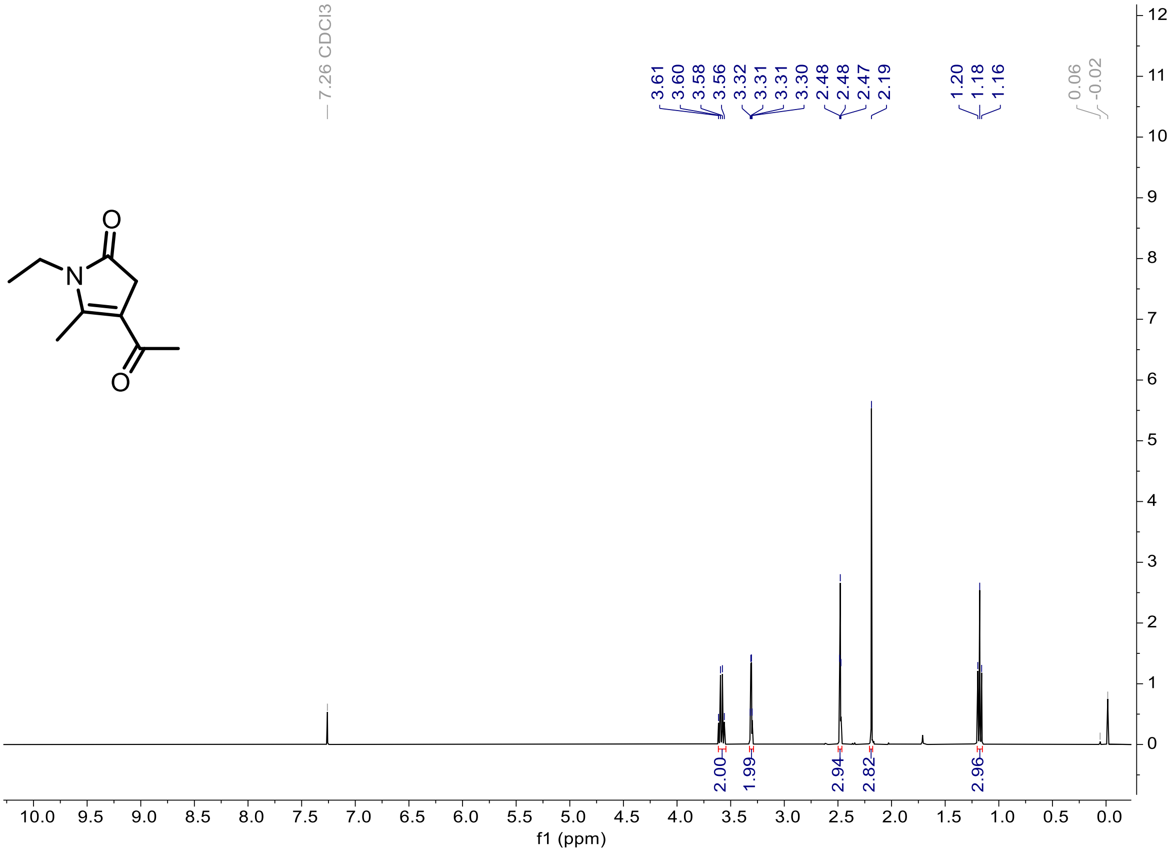

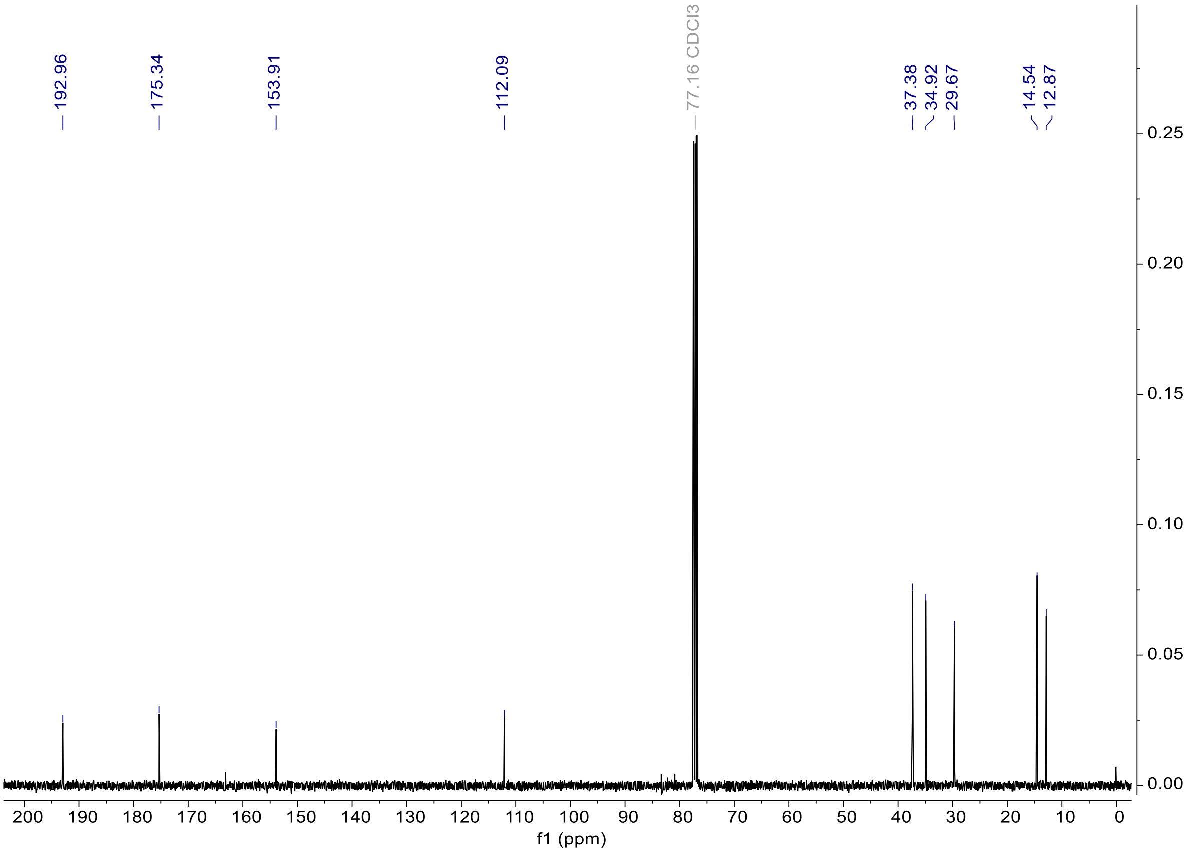

NMR spectra for **7i**

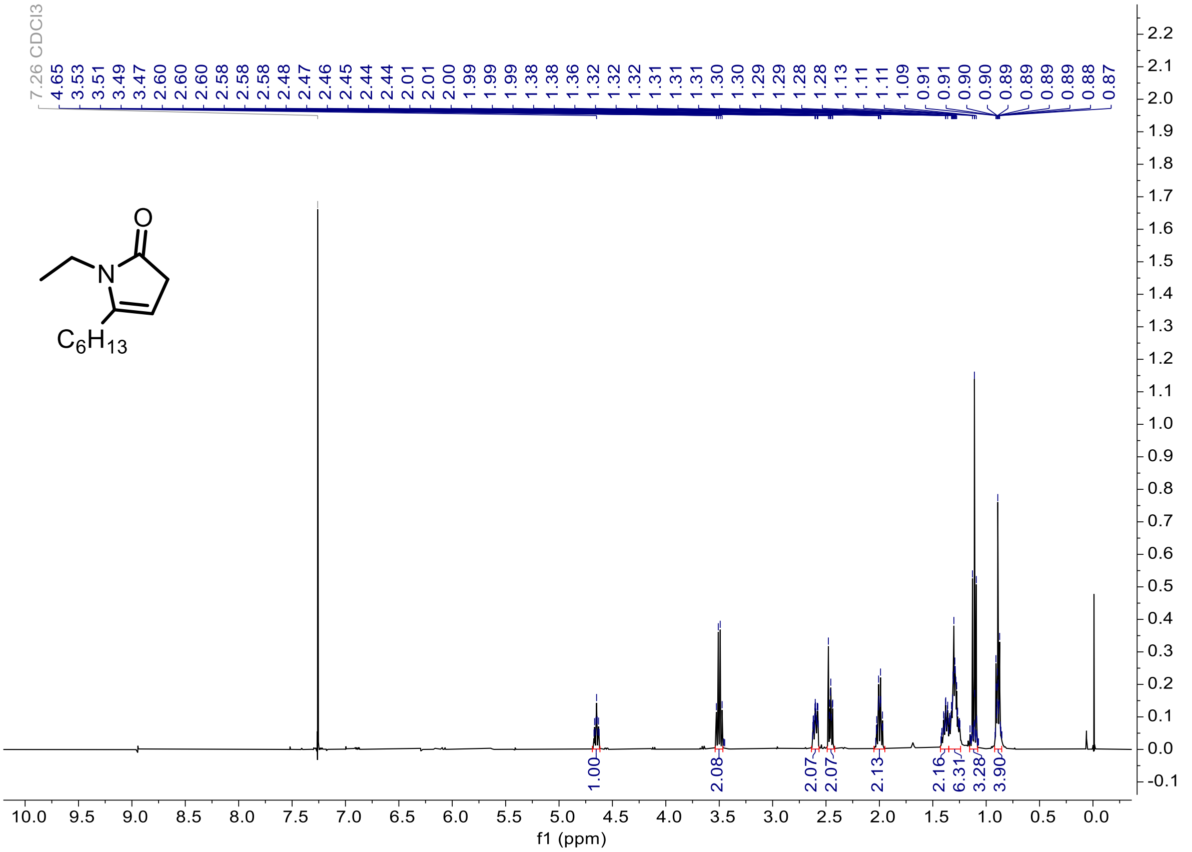

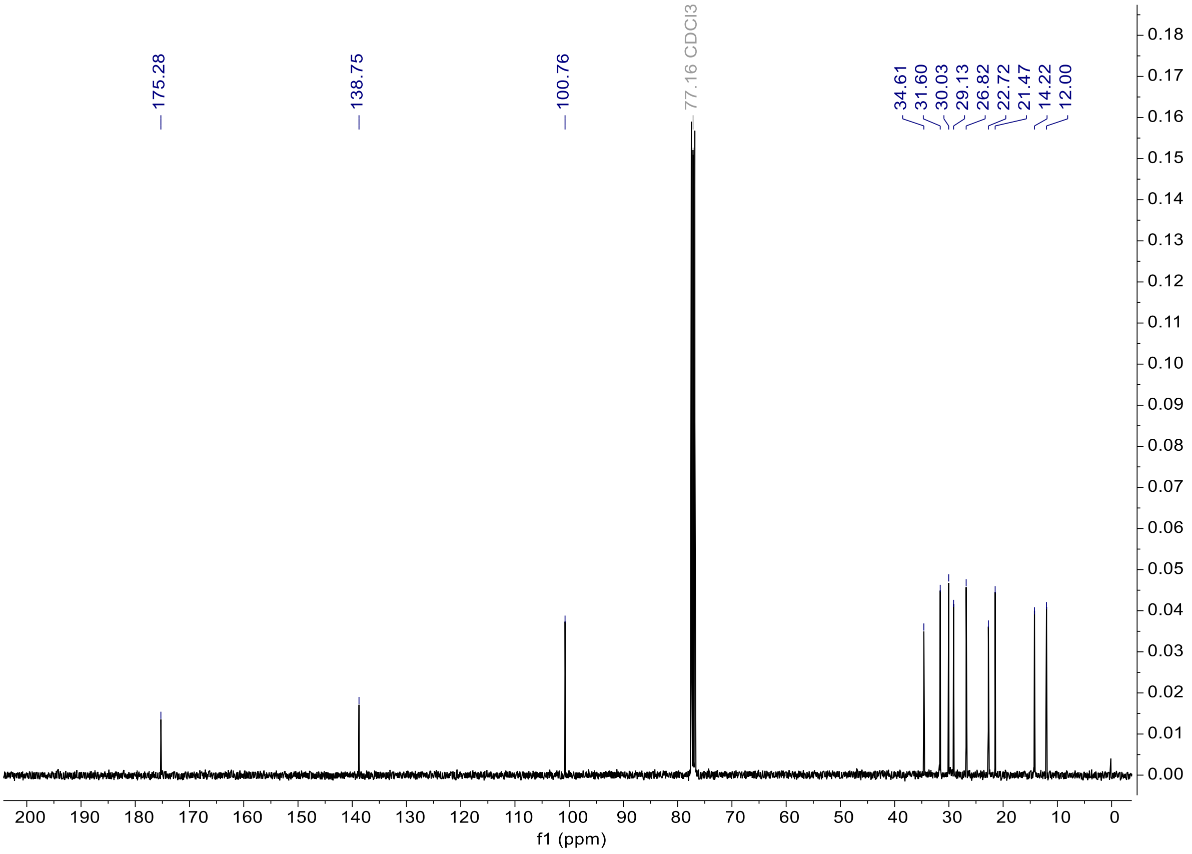

NMR spectra for **7j**

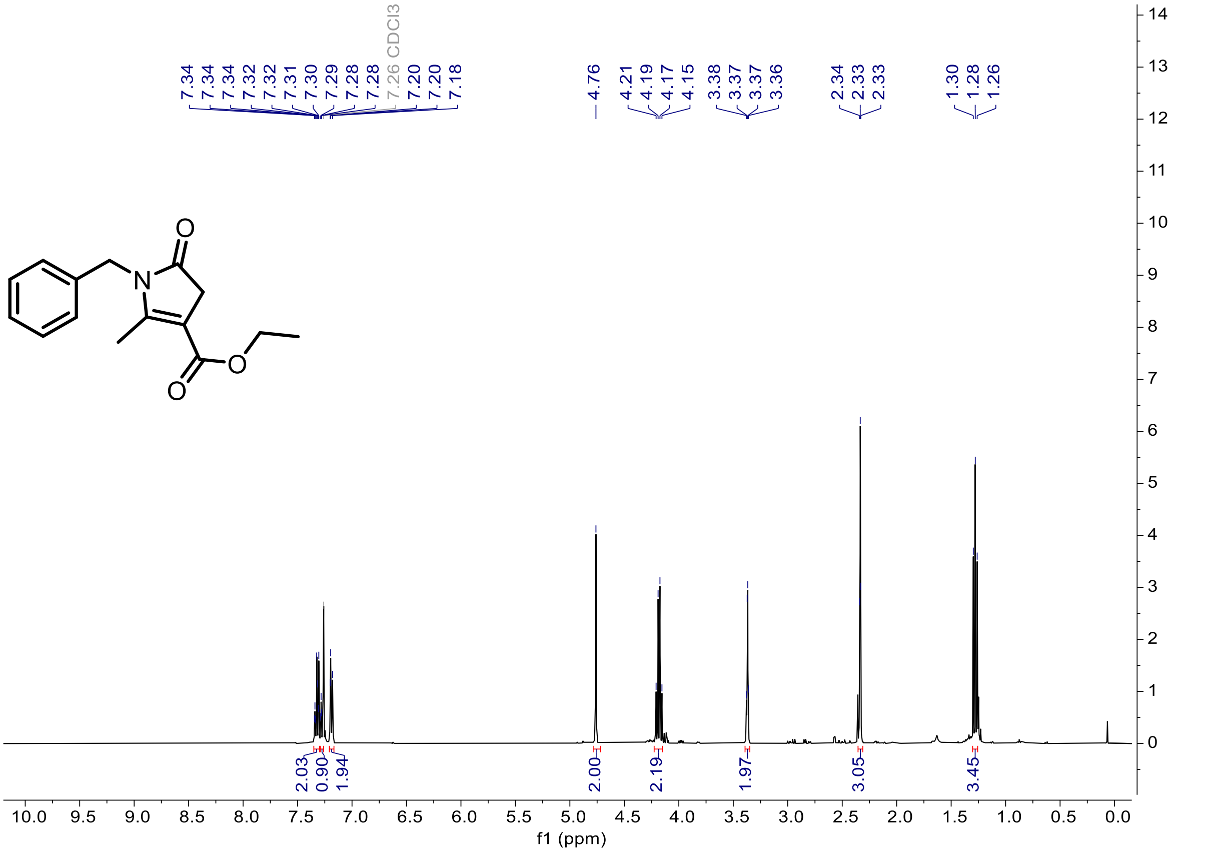

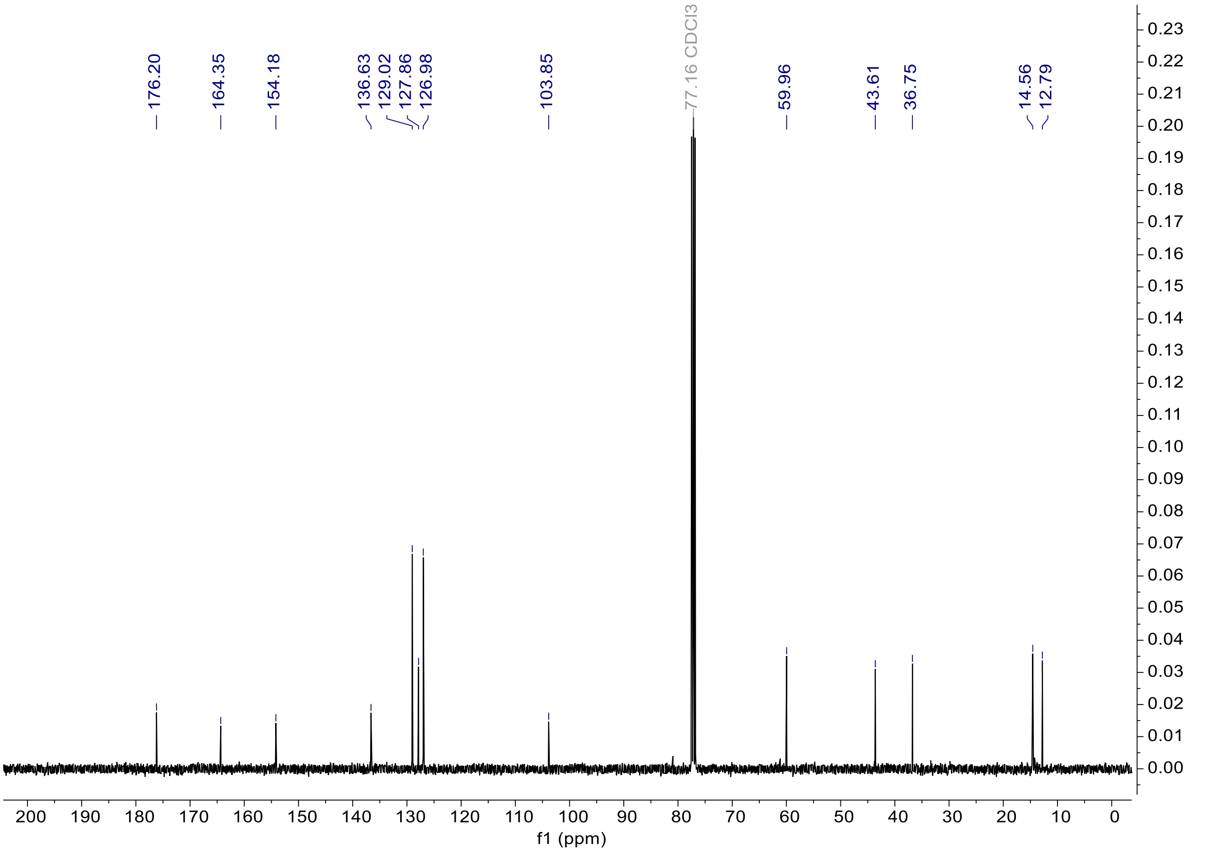

NMR spectra for **7k**

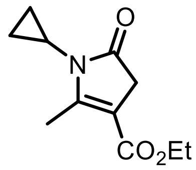

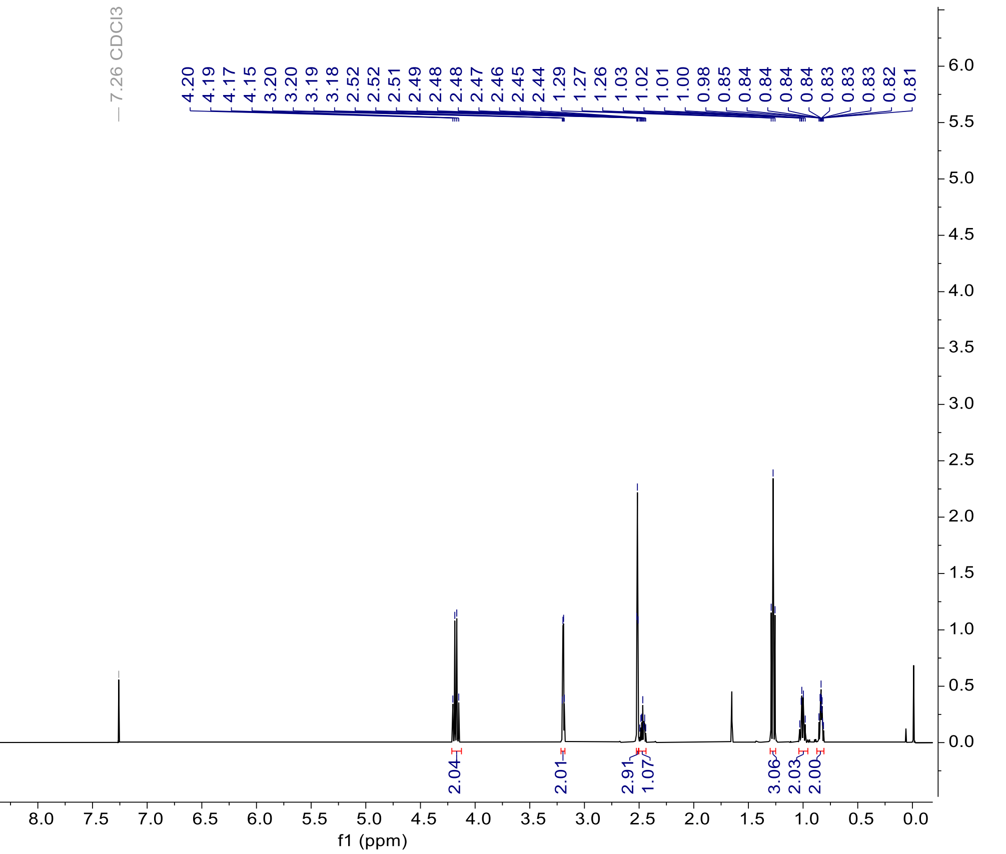

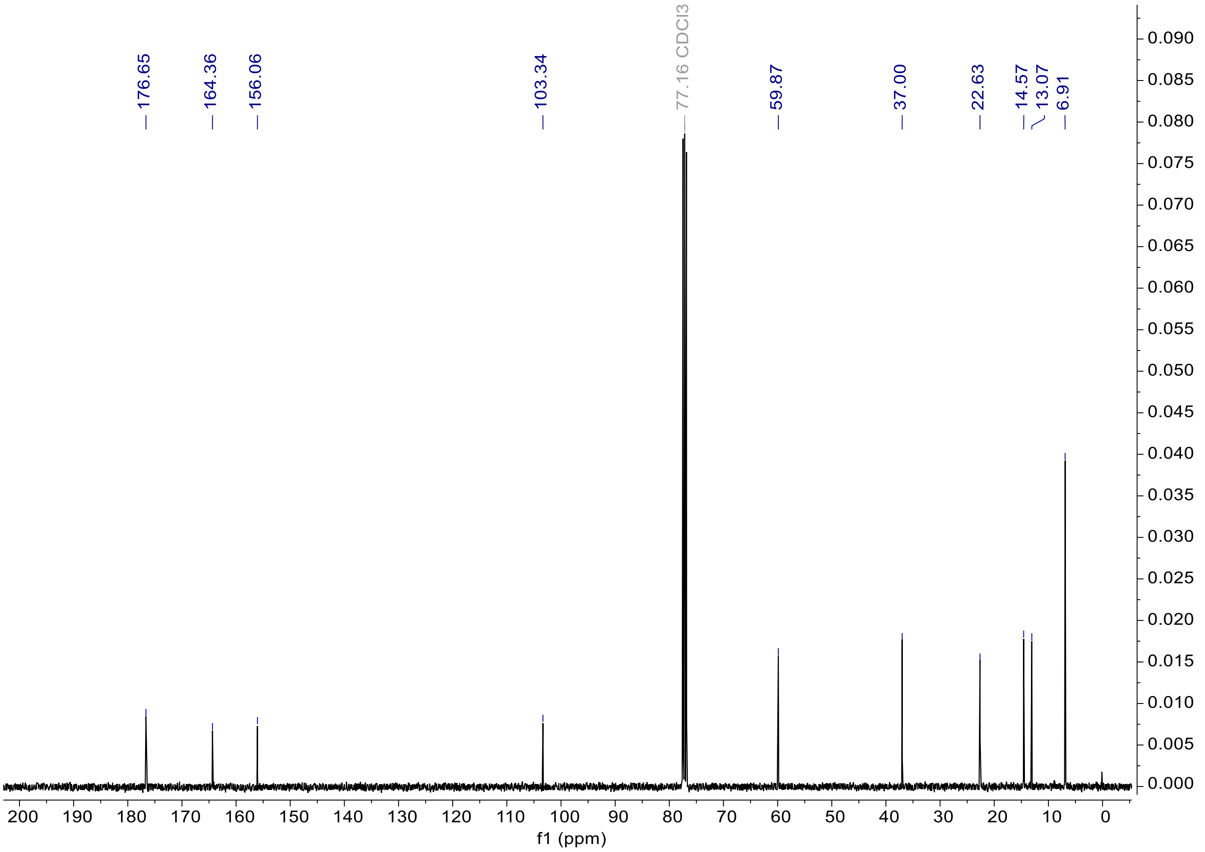

Data for **7l**

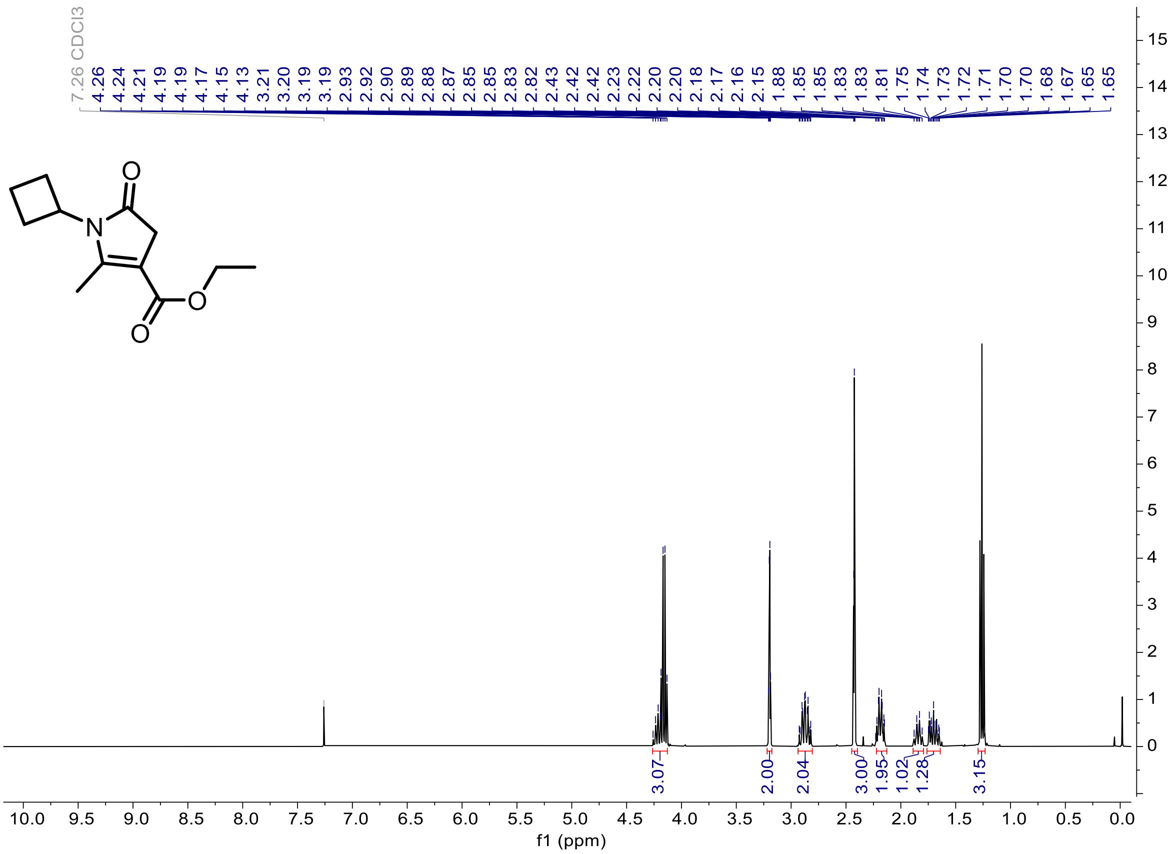
O
N
O
O
7.26 CDCl3
4.26 4.24 4.21 4.19 4.19 4.17 4.15 4.13 3.21 3.20 3.19 3.19 2.93 2.92 2.90 2.89 2.88 2.87 2.85 2.85 2.83 2.82 2.43 2.42 2.42 2.23 2.22 2.20 2.20 2.18 2.17 2.16 2.15 1.88 1.85 1.85 1.83 1.83 1.81 1.75 1.74 1.73 1.72 1.71 1.70 1.70 1.68 1.67 1.65 1.65
3.07 2.00 2.04 3.00 1.95 1.02 1.28 3.15
f1 (ppm)

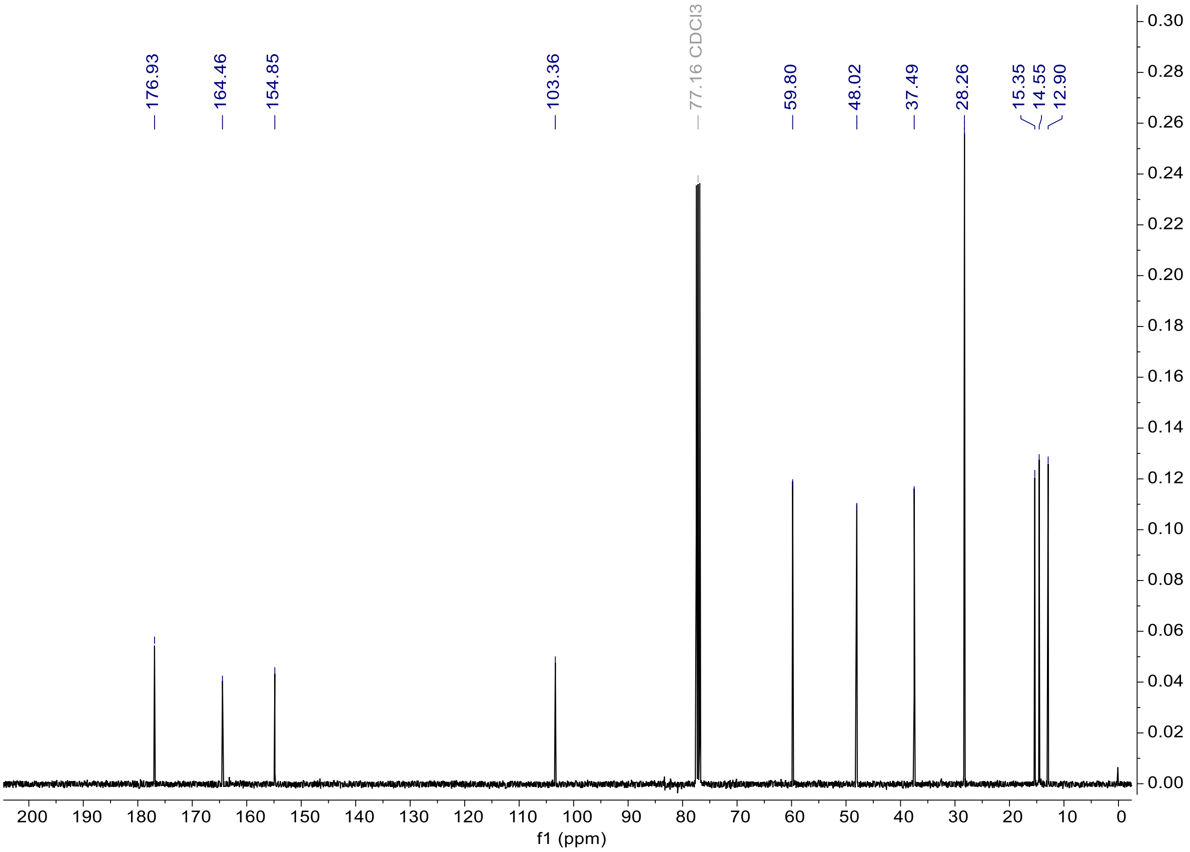
176.93
164.46
154.85
103.36
77.16 CDCl3
59.80
48.02
37.49
28.26
15.35
14.55
12.90
f1 (ppm)

Data for **7m**

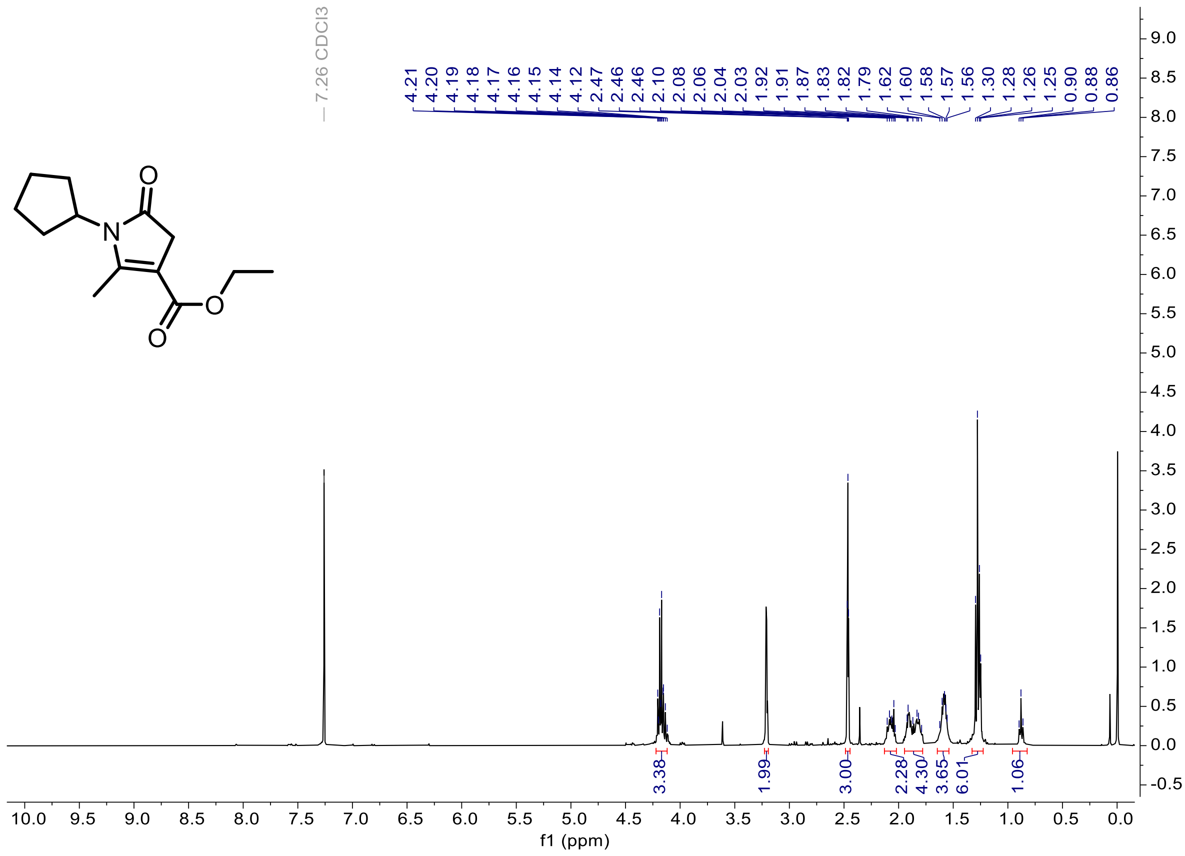

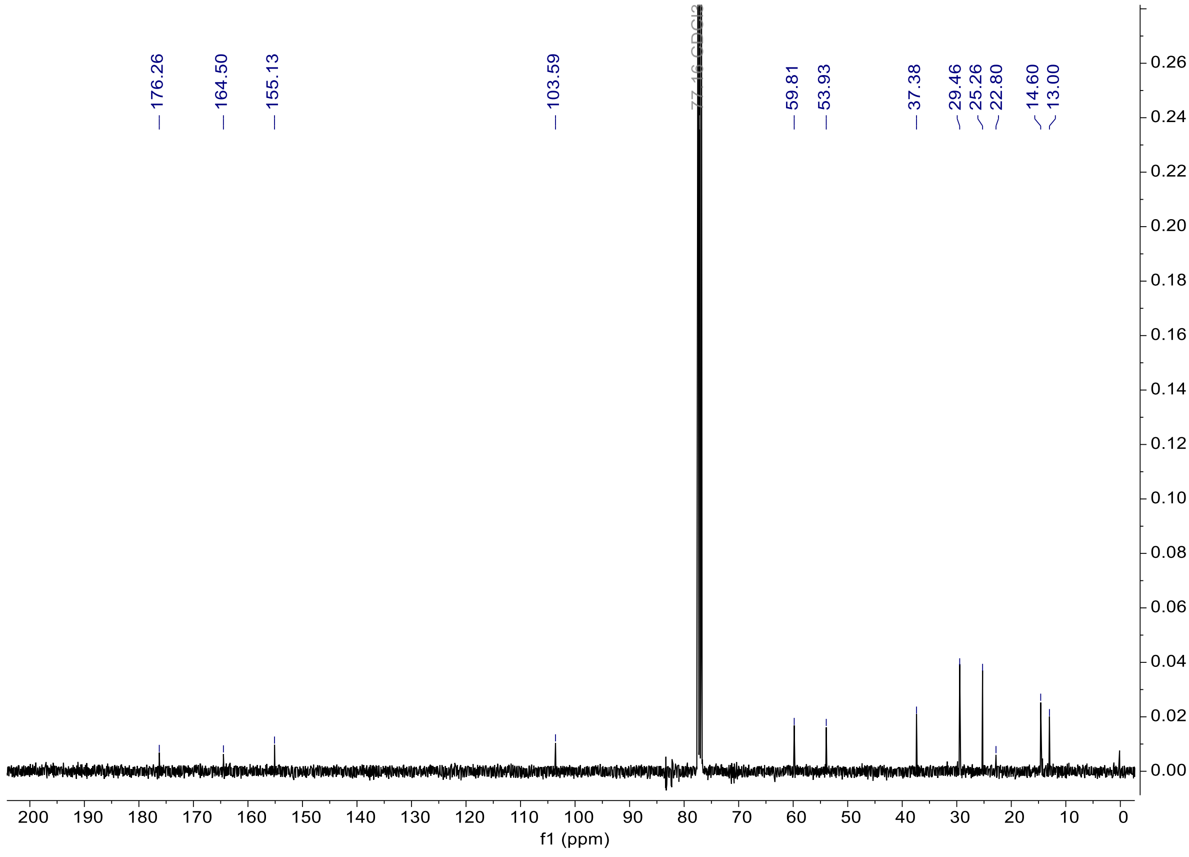

NMR spectra for **7n**

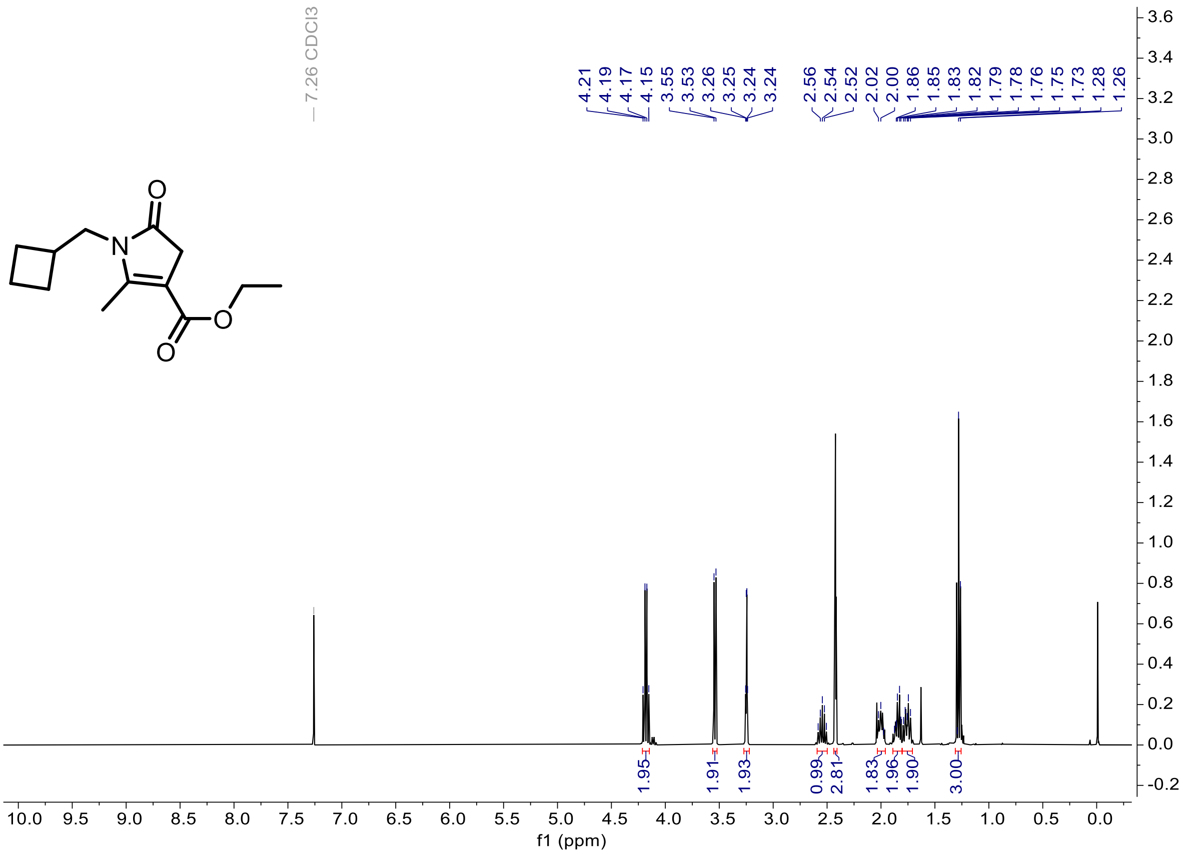

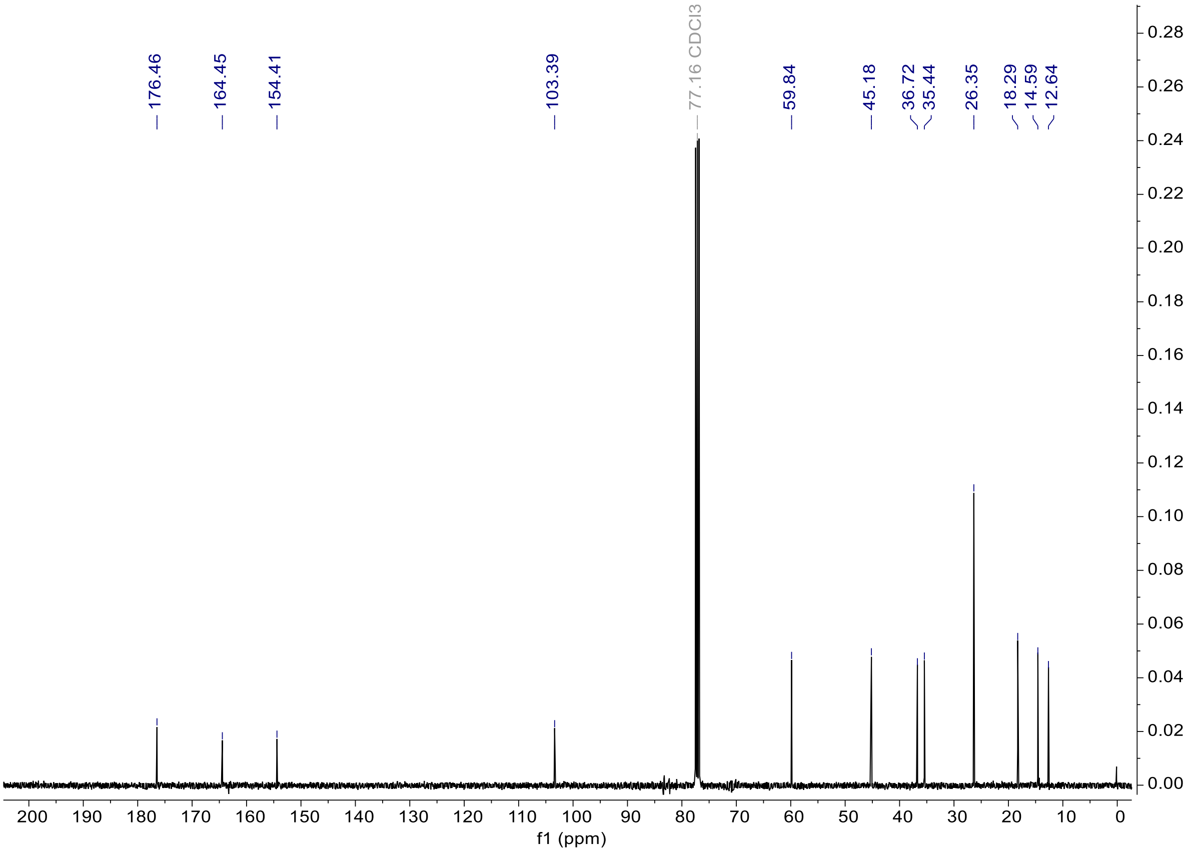

NMR spectra for **7o**

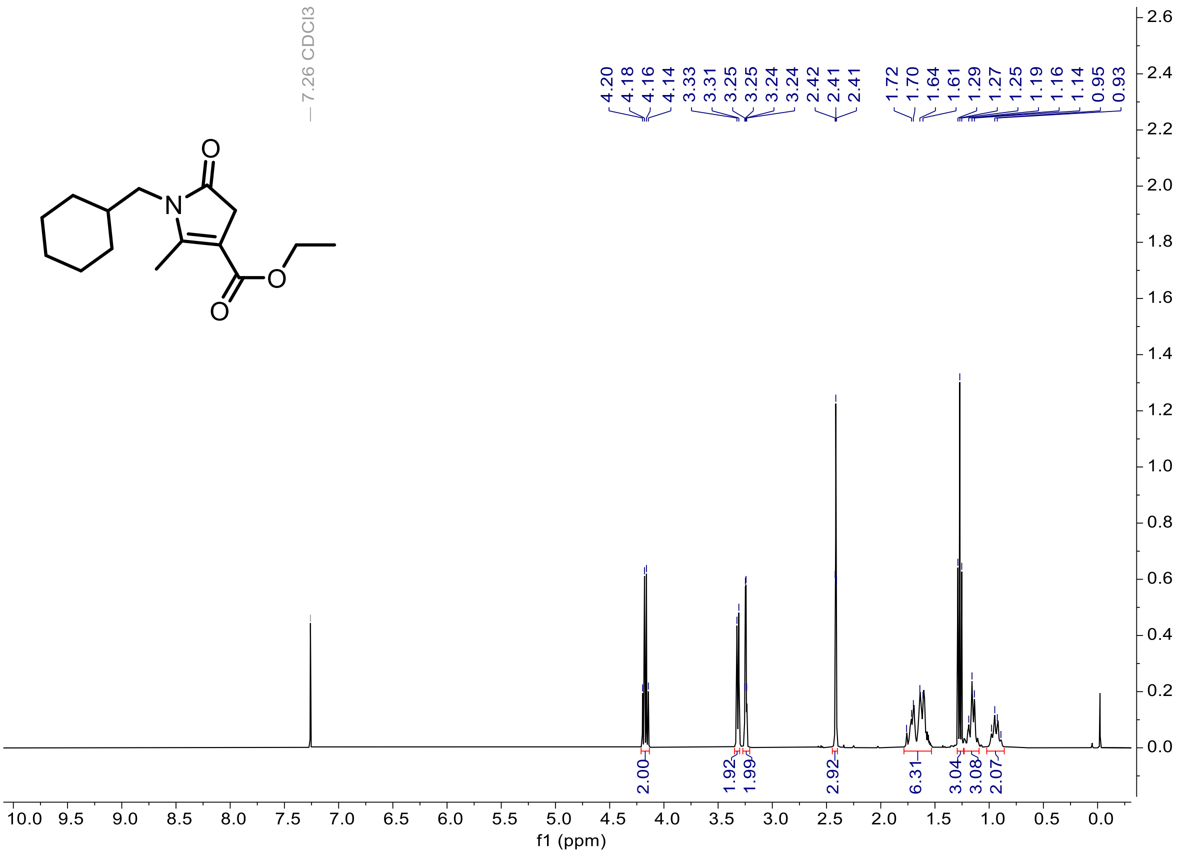

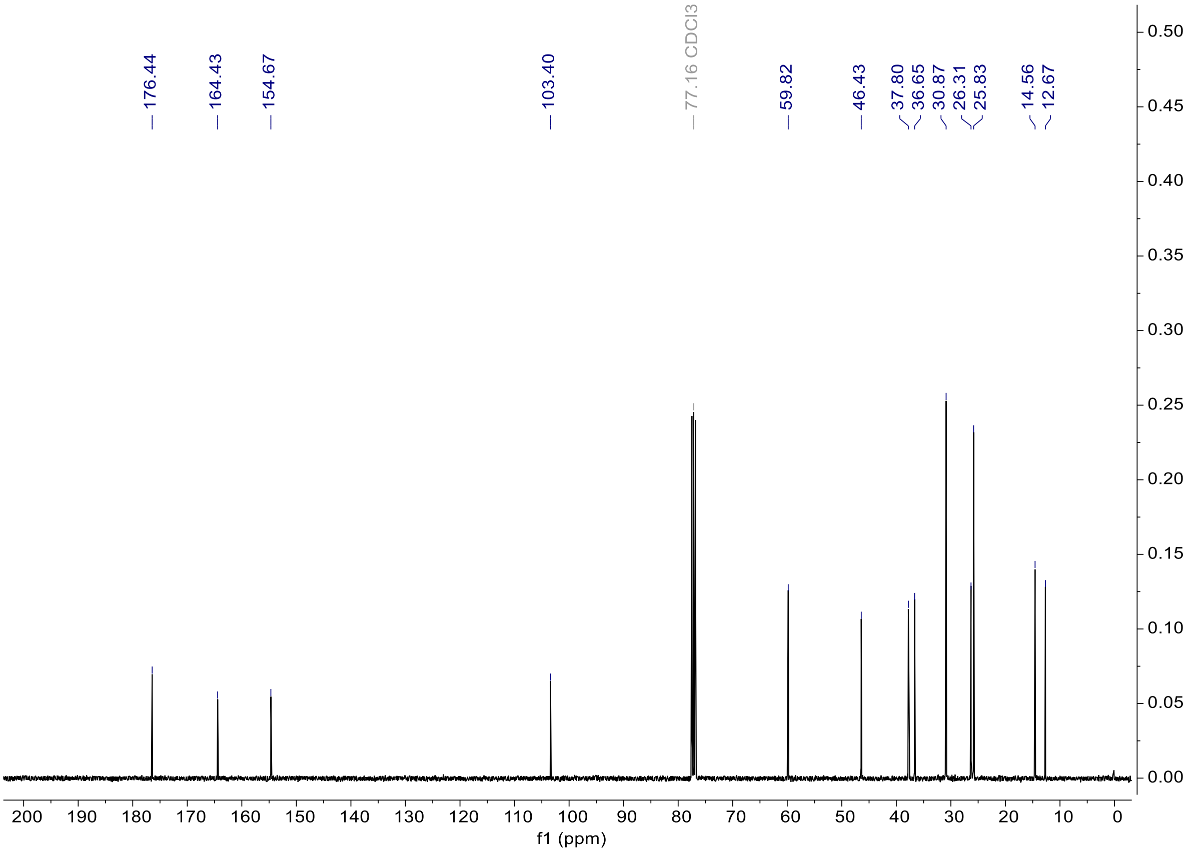

NMR spectra for **7p**

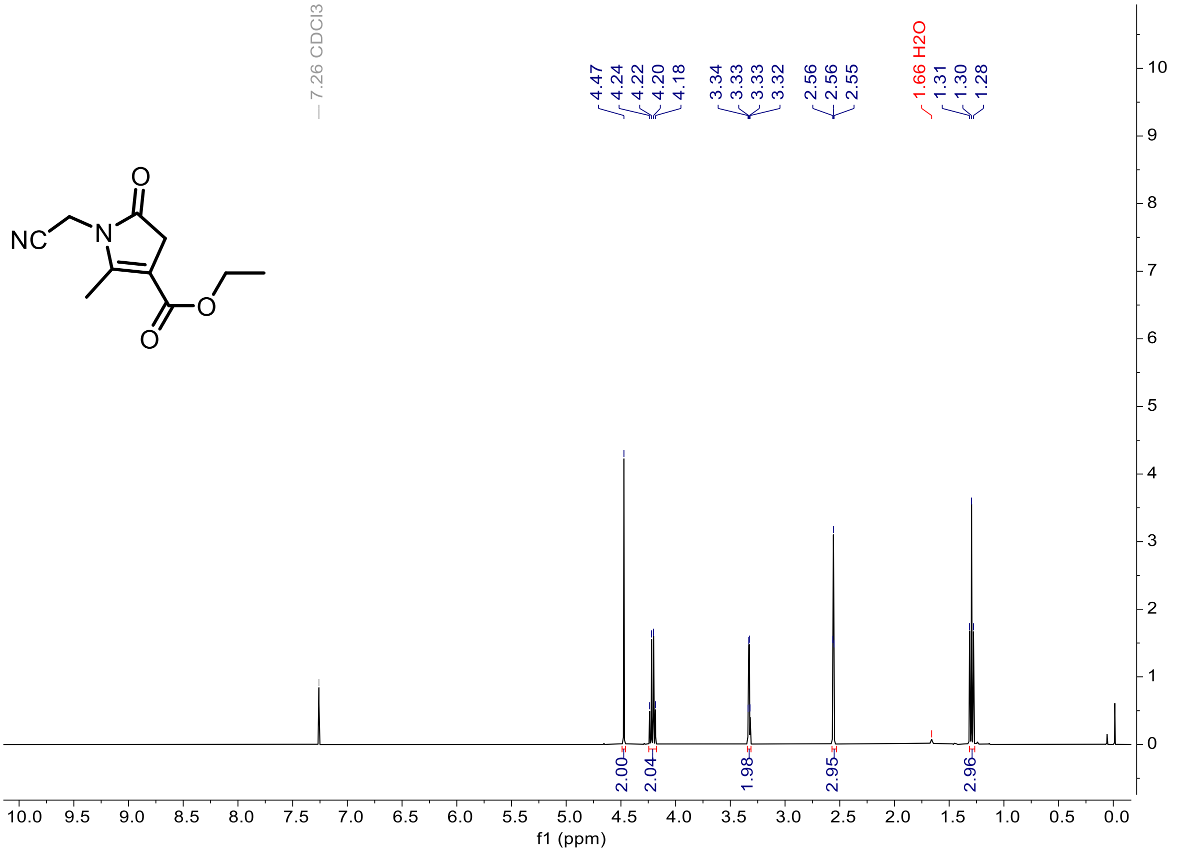

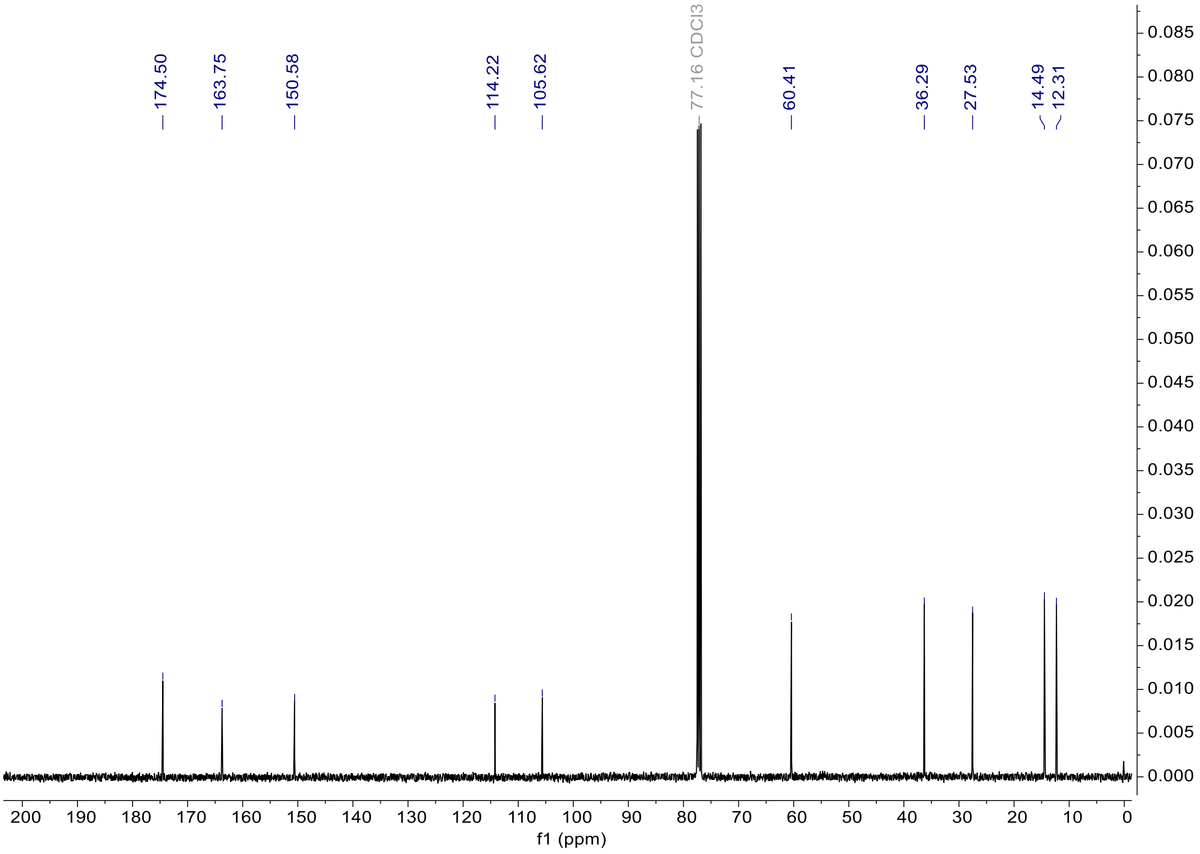

NMR spectra for **7q**

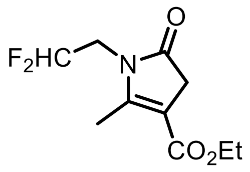

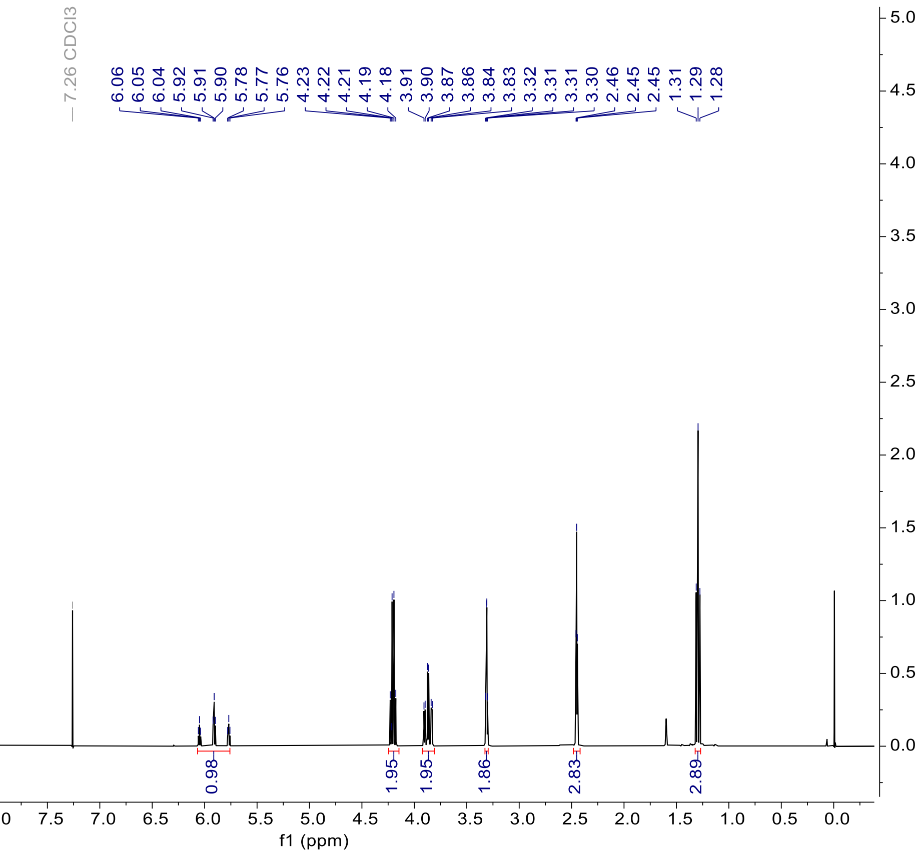

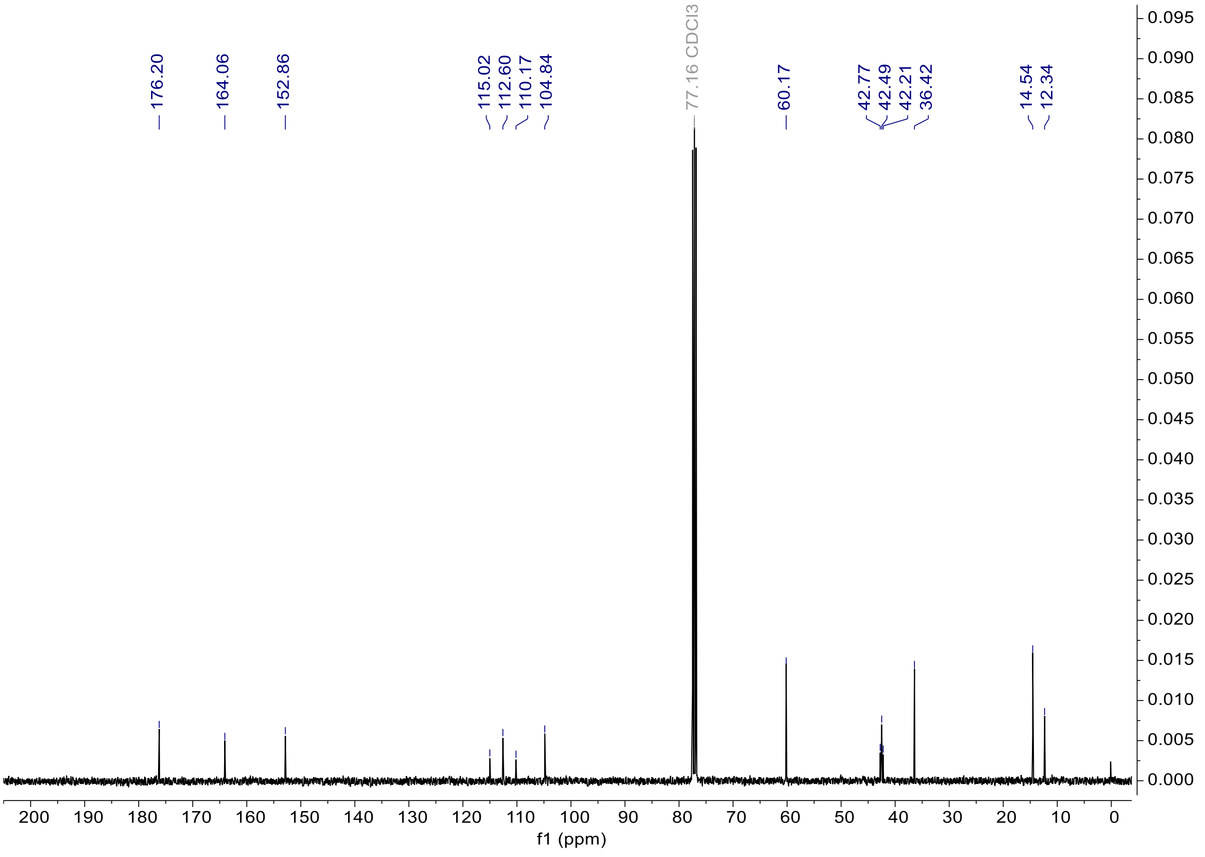

NMR spectra for **7r**

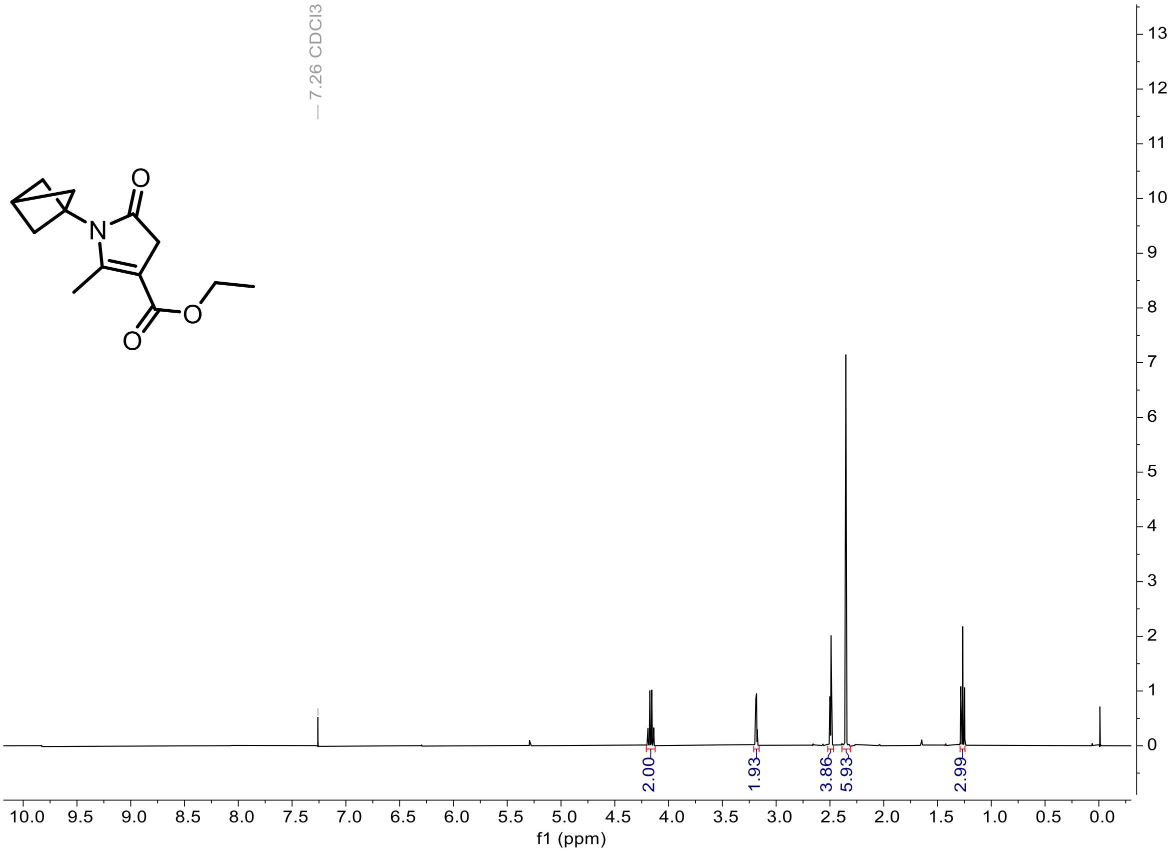

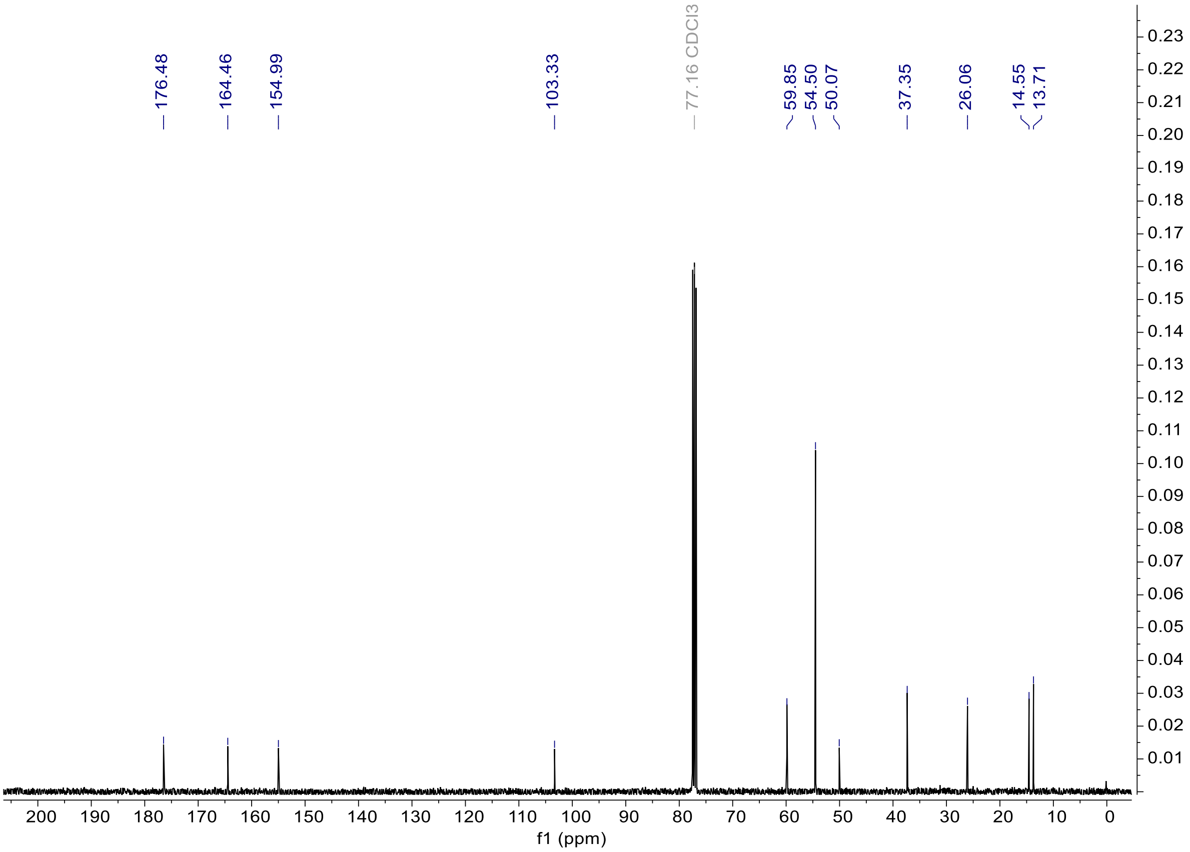

NMR spectra for **7s**

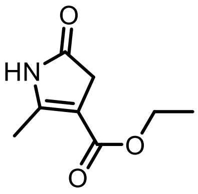

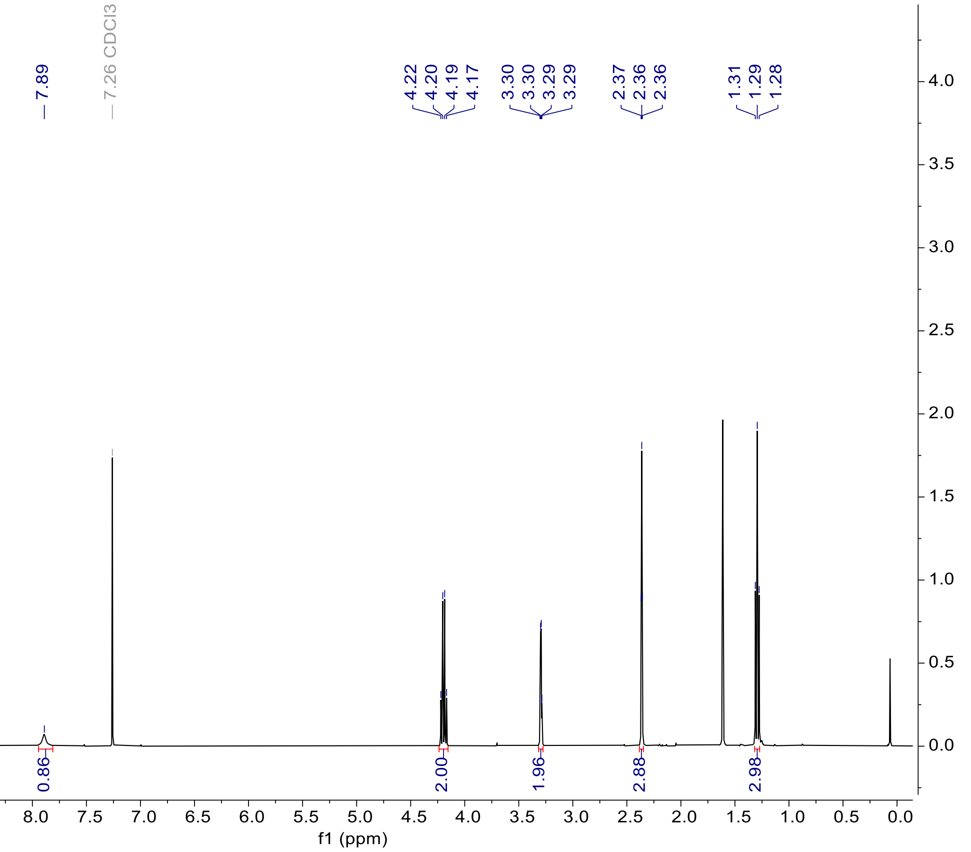

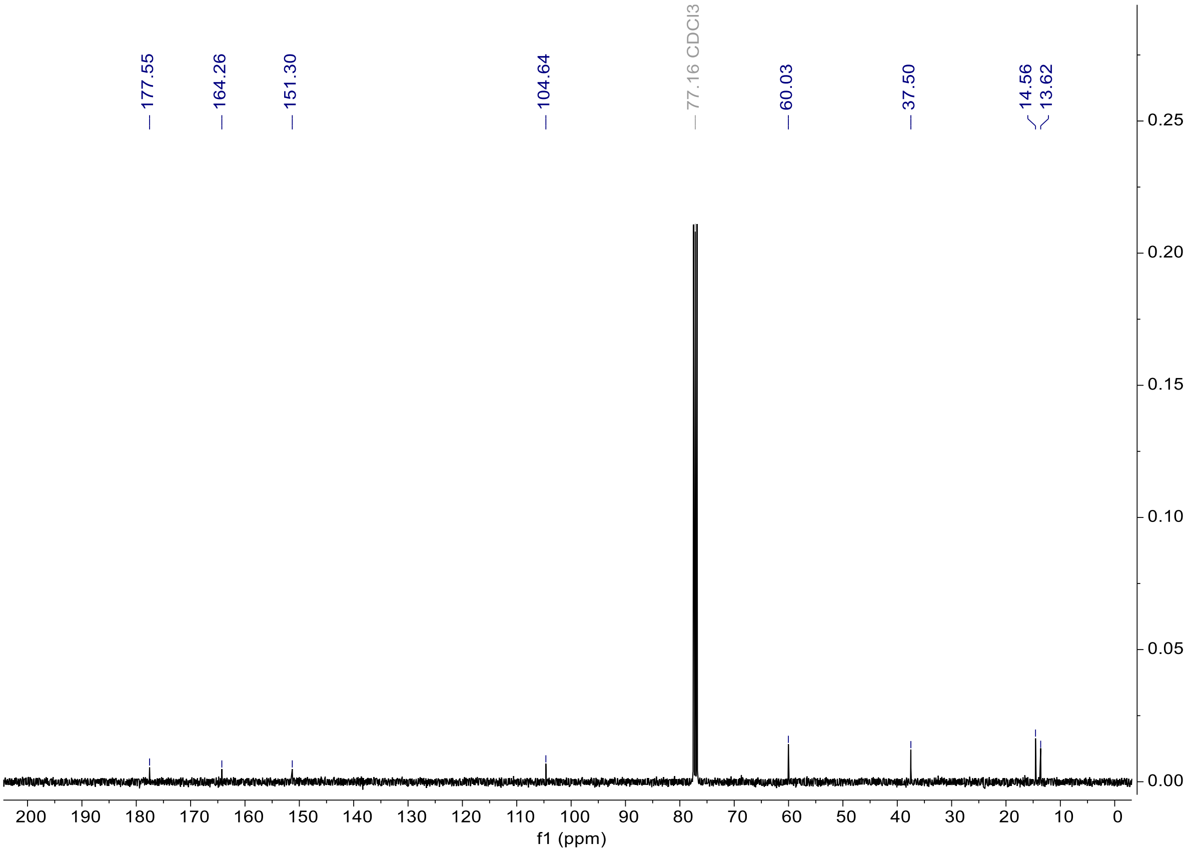

NMR spectra for **7t**

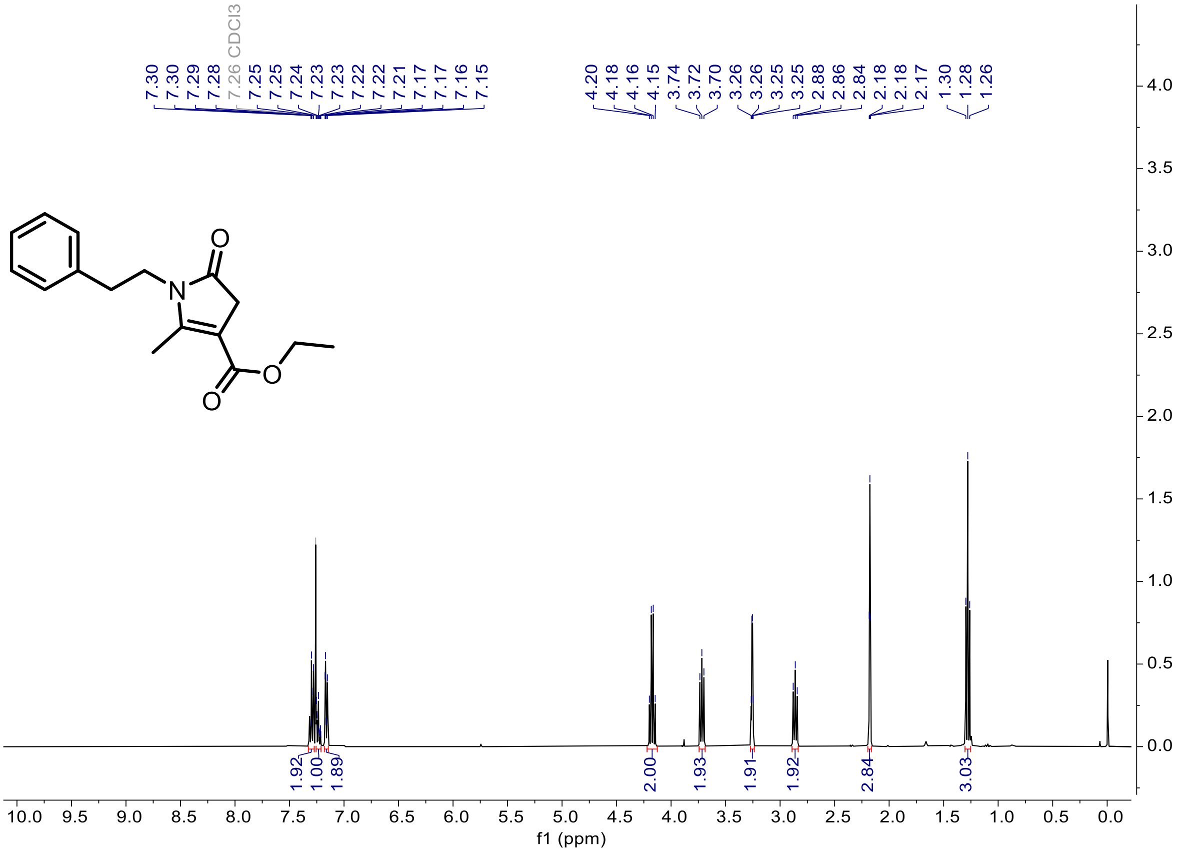

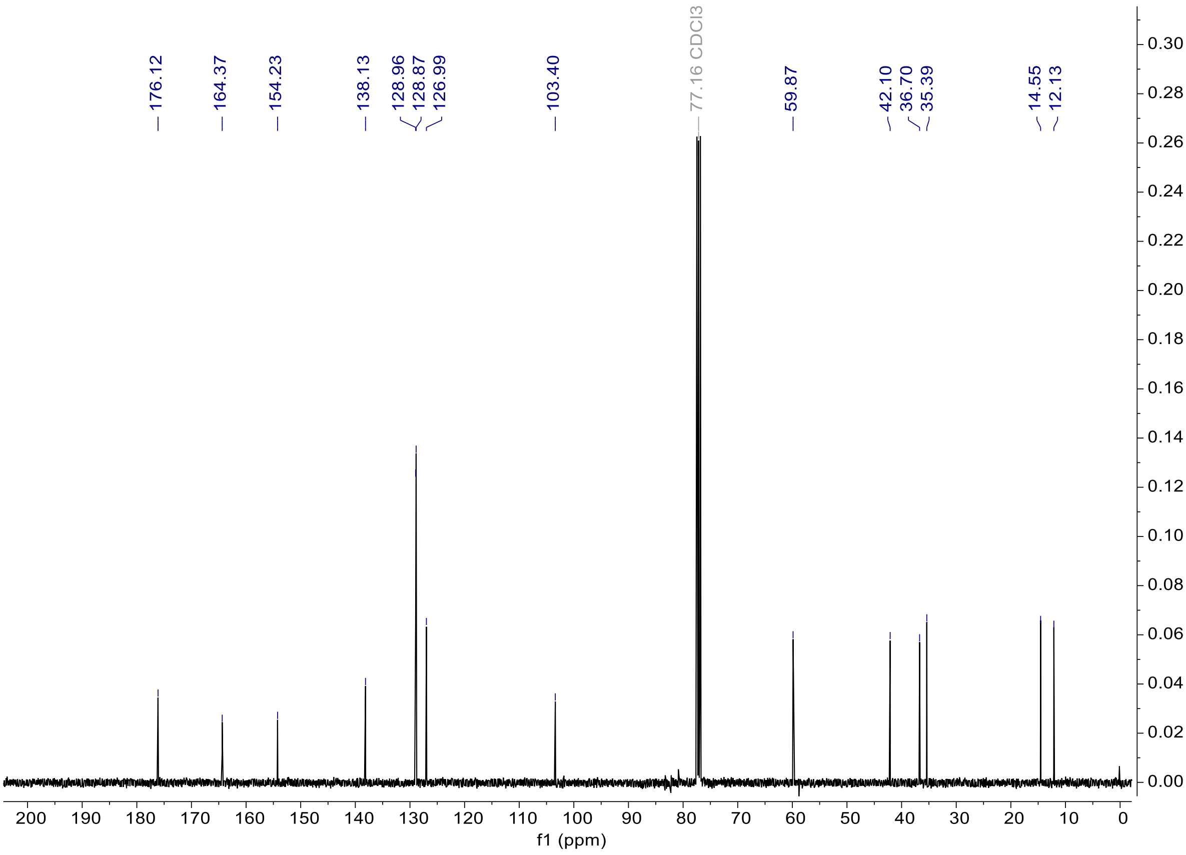

NMR spectra for **7u**

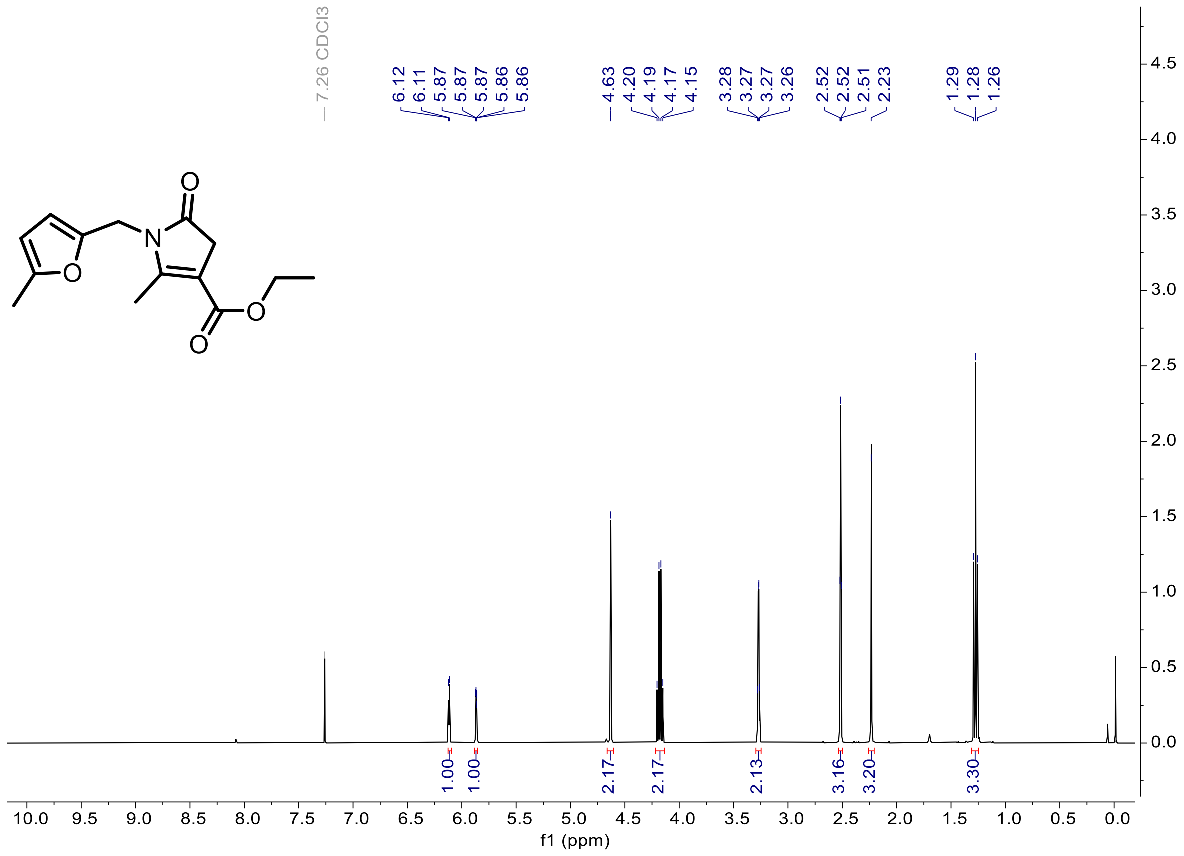

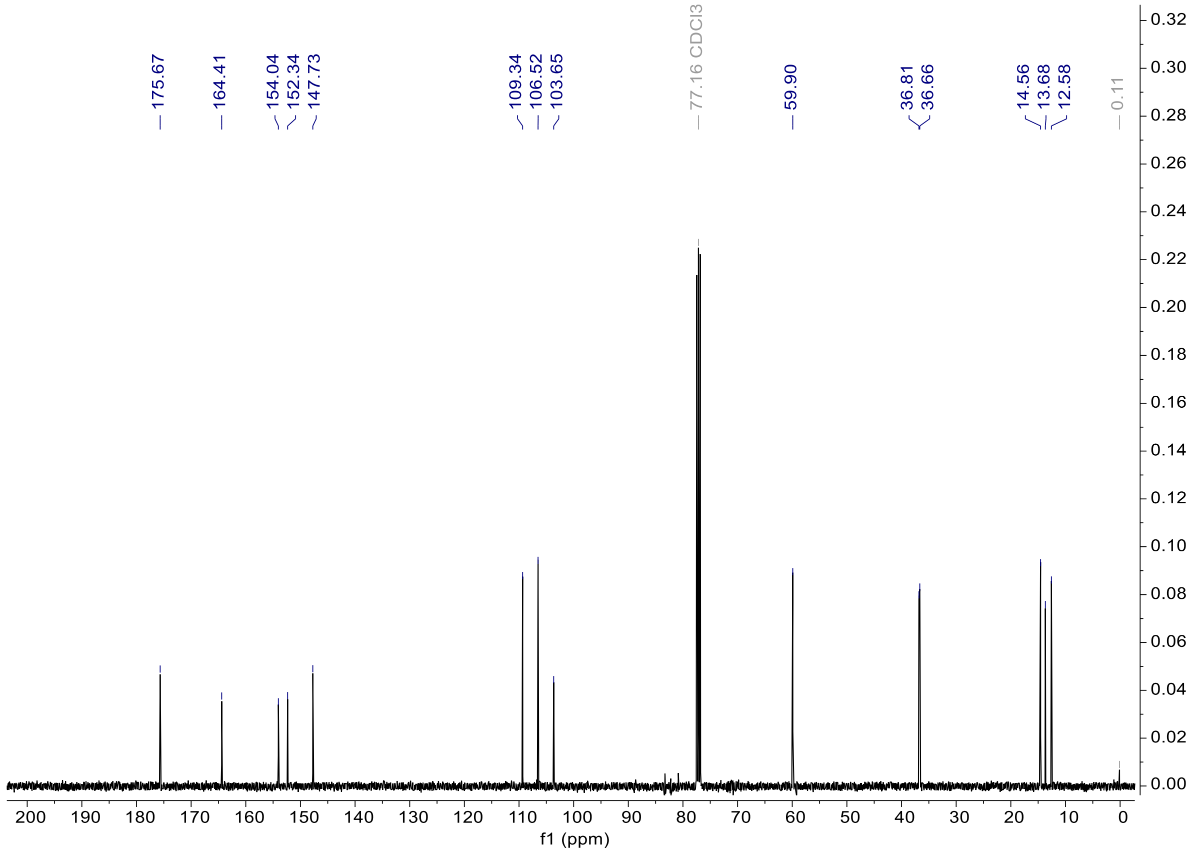

NMR spectra for **7v**

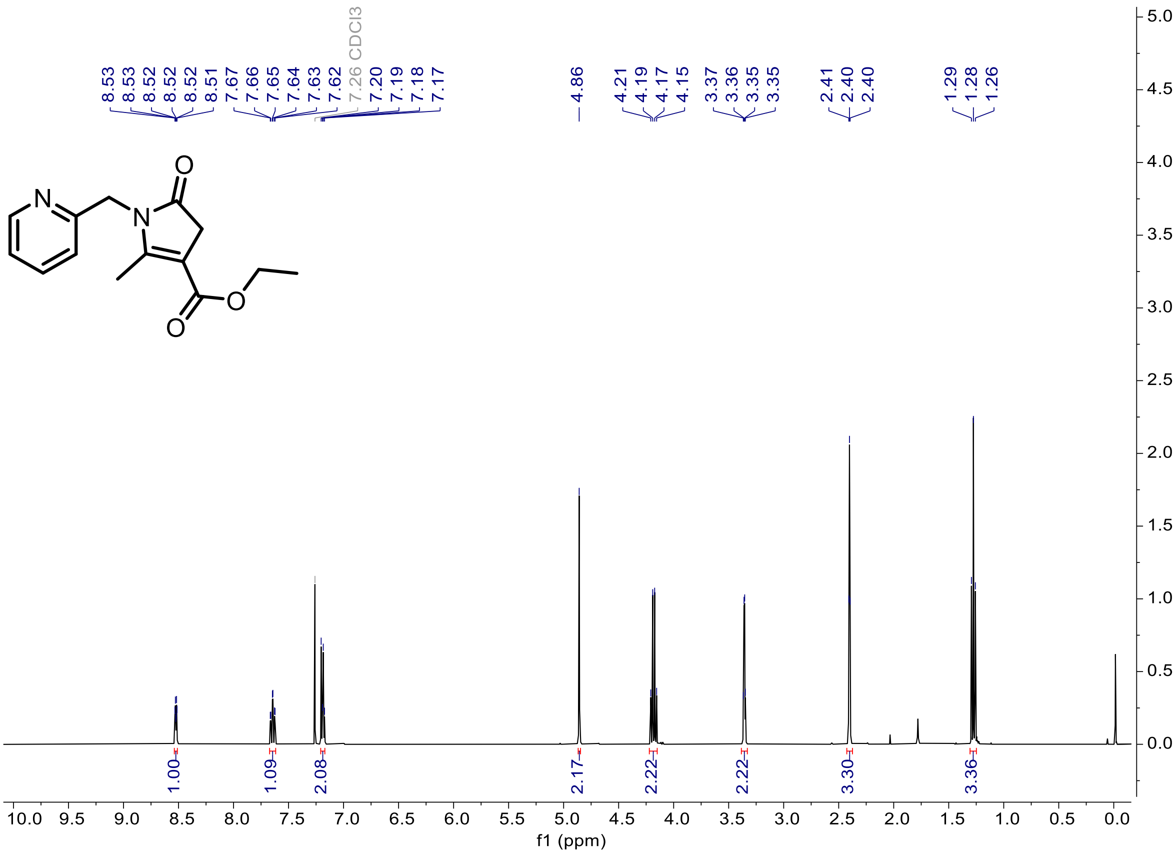

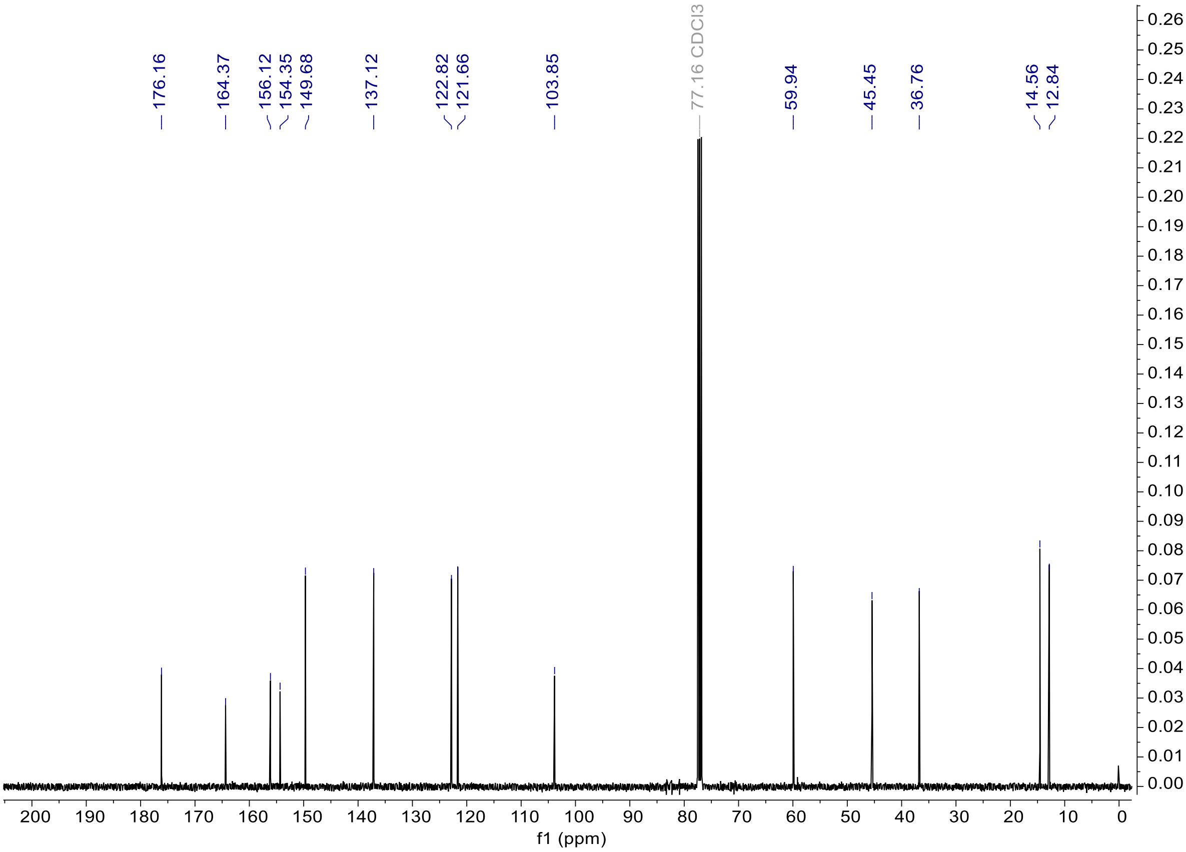

NMR spectra for **7w**

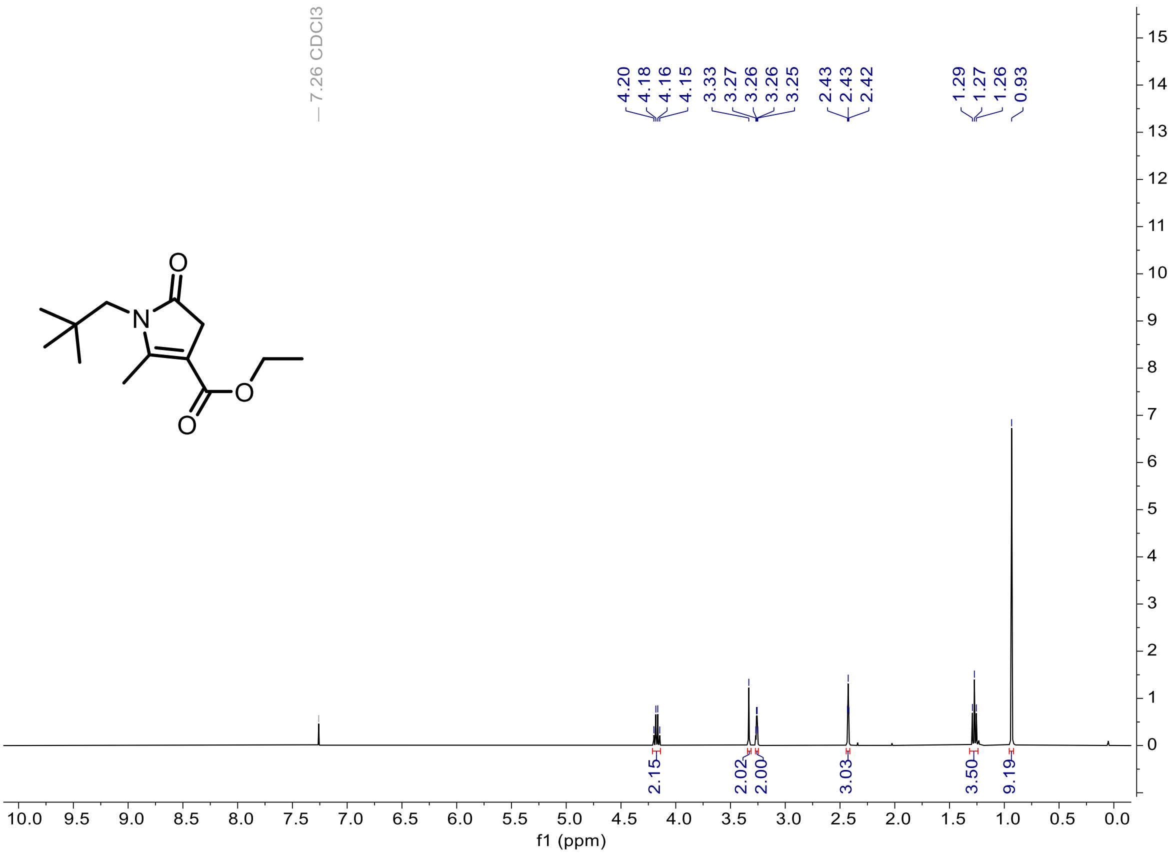

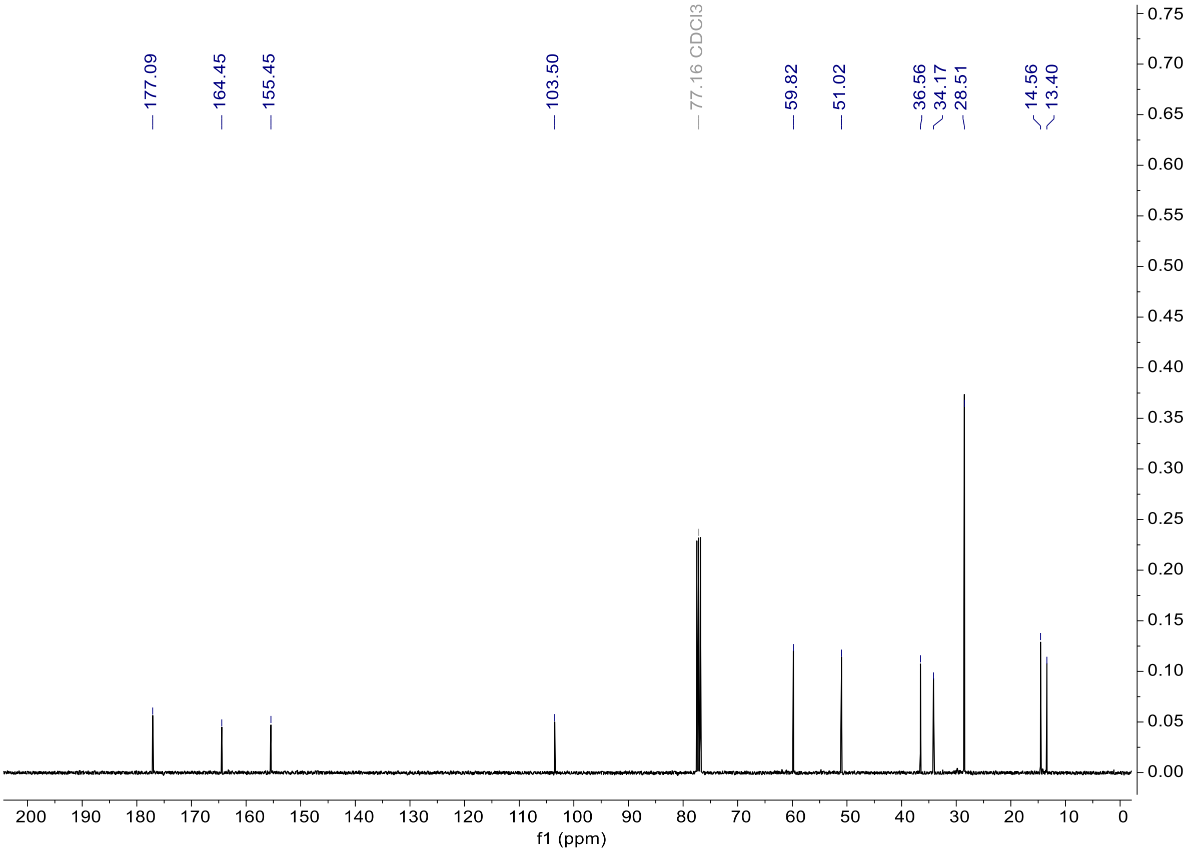

NMR spectra for **7x**

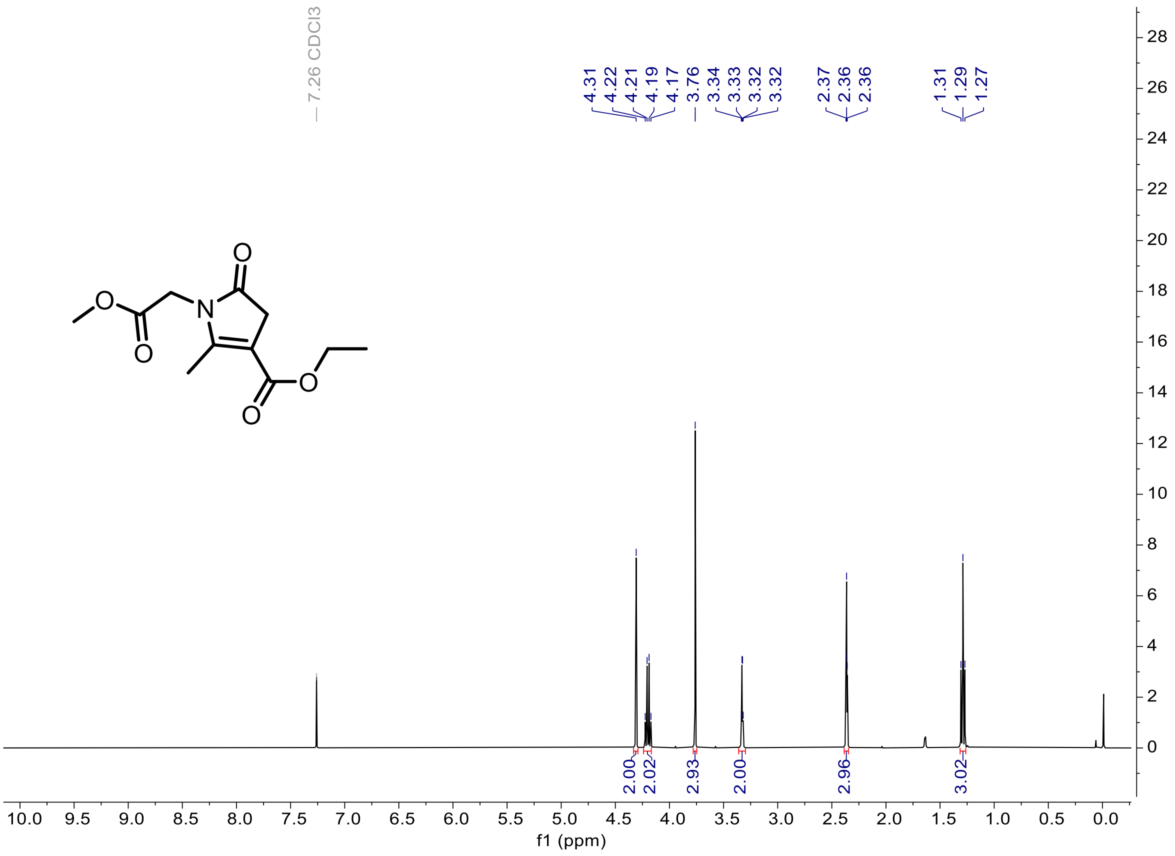

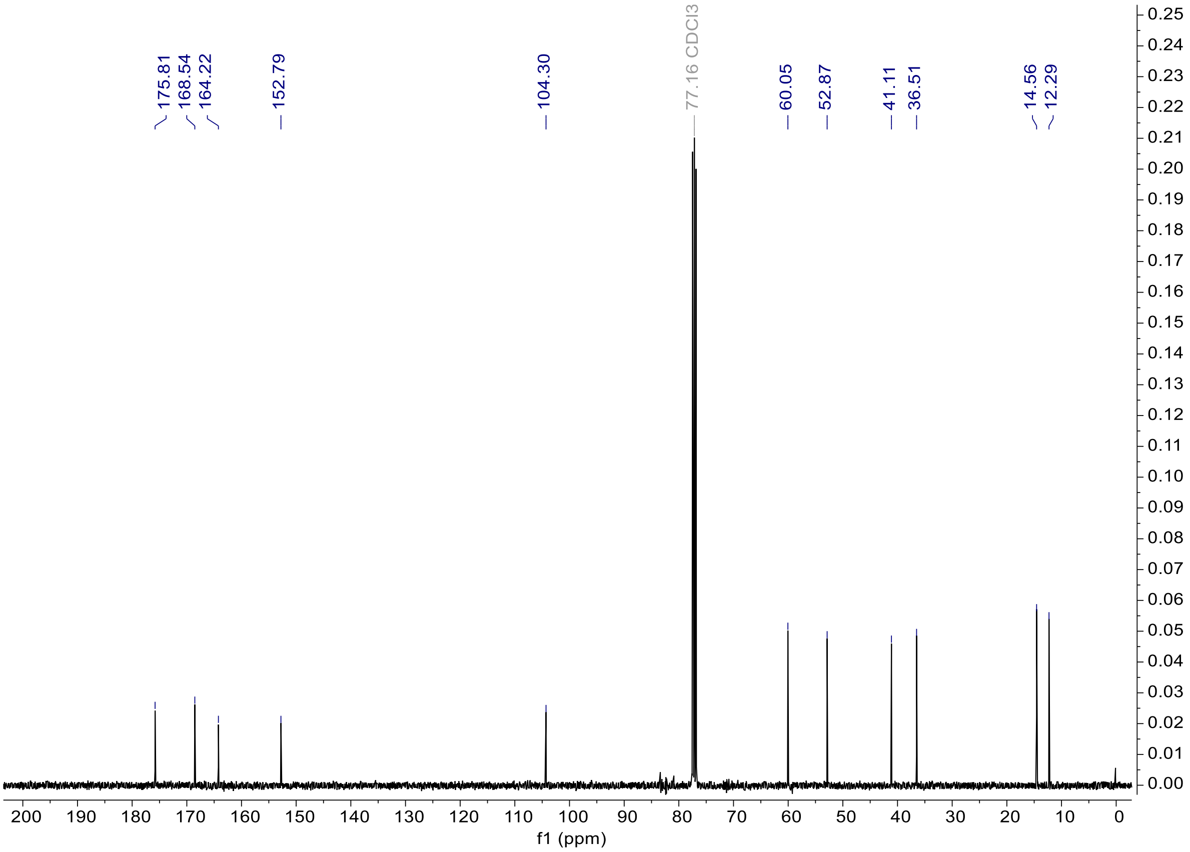

NMR spectra for **7y**

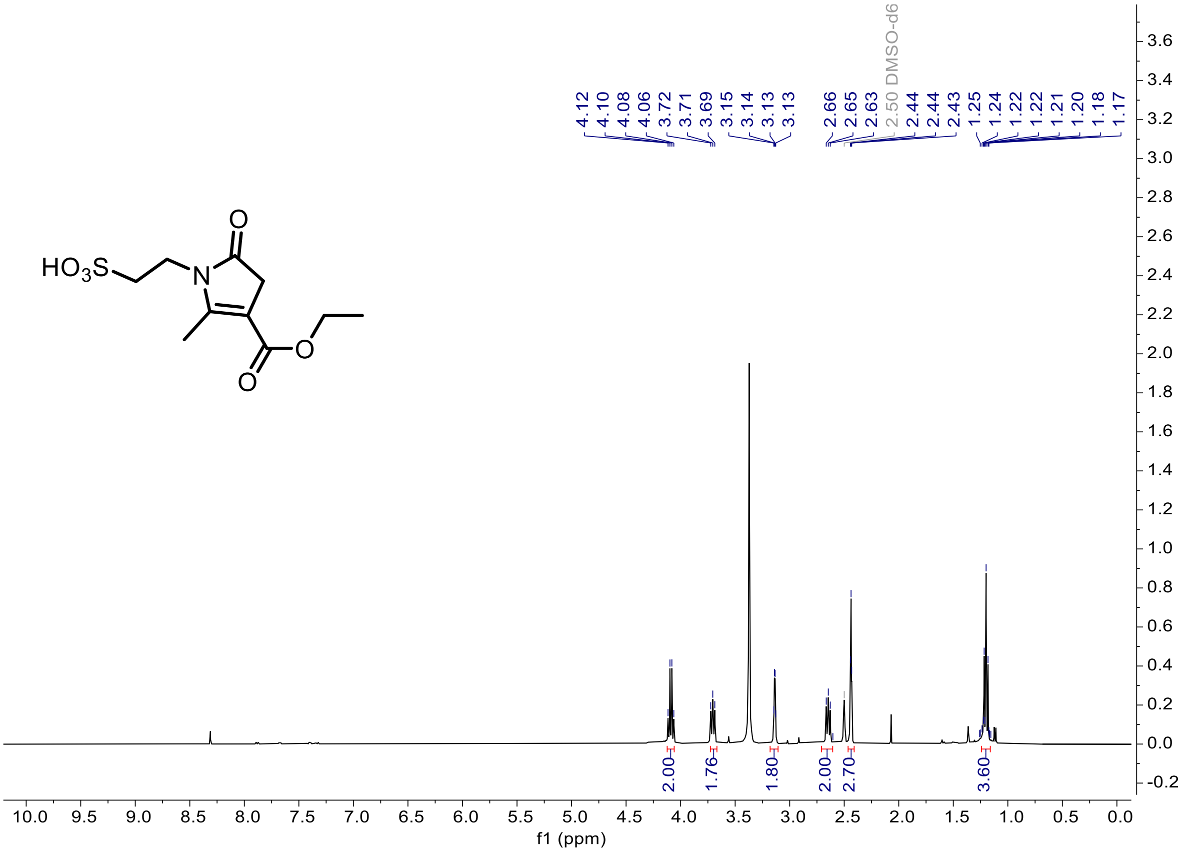

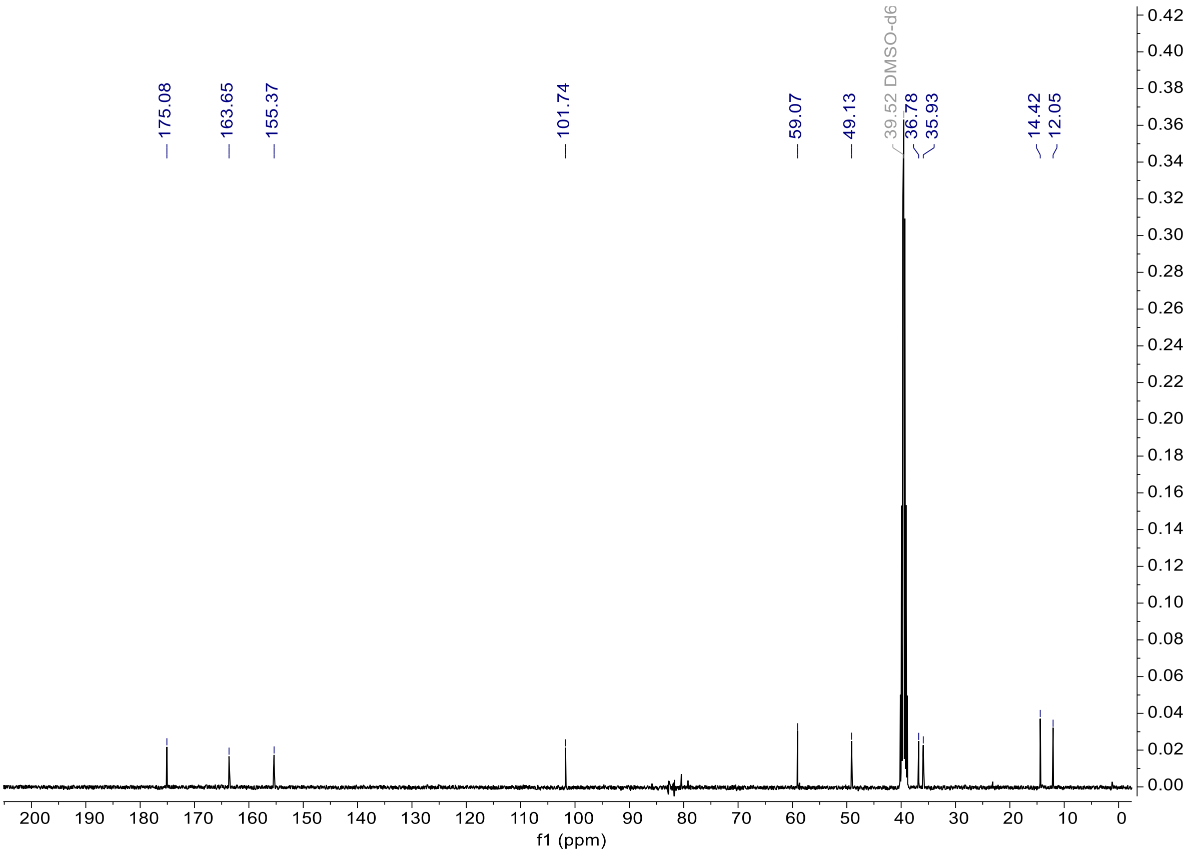

NMR spectra for **7z**

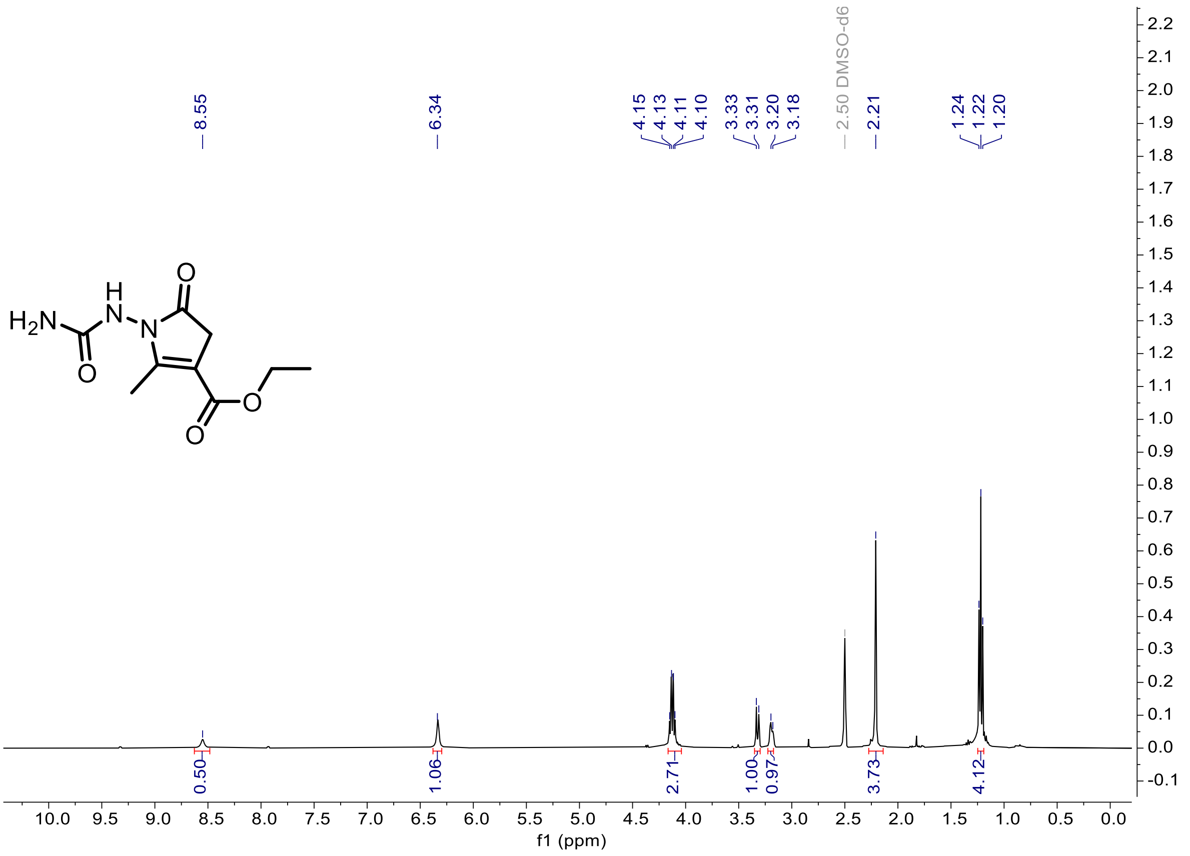

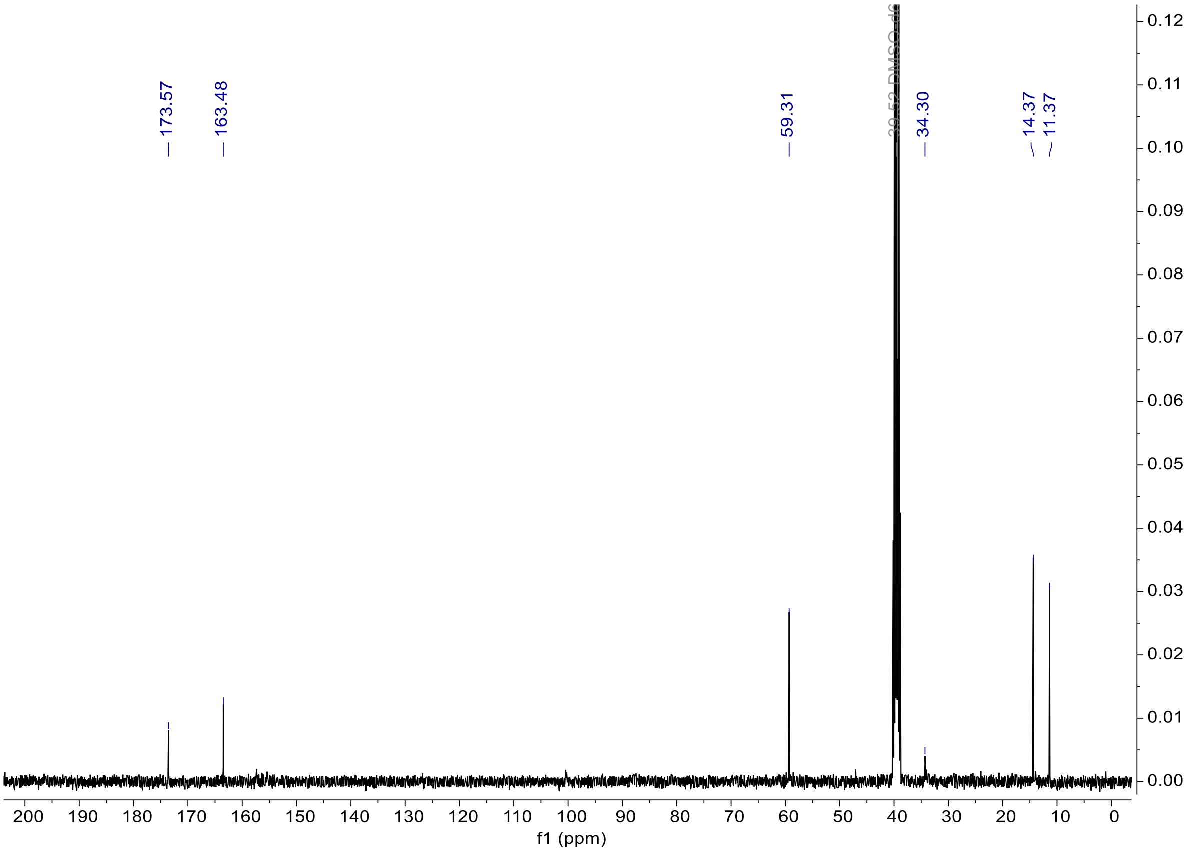

NMR spectra for **7za**

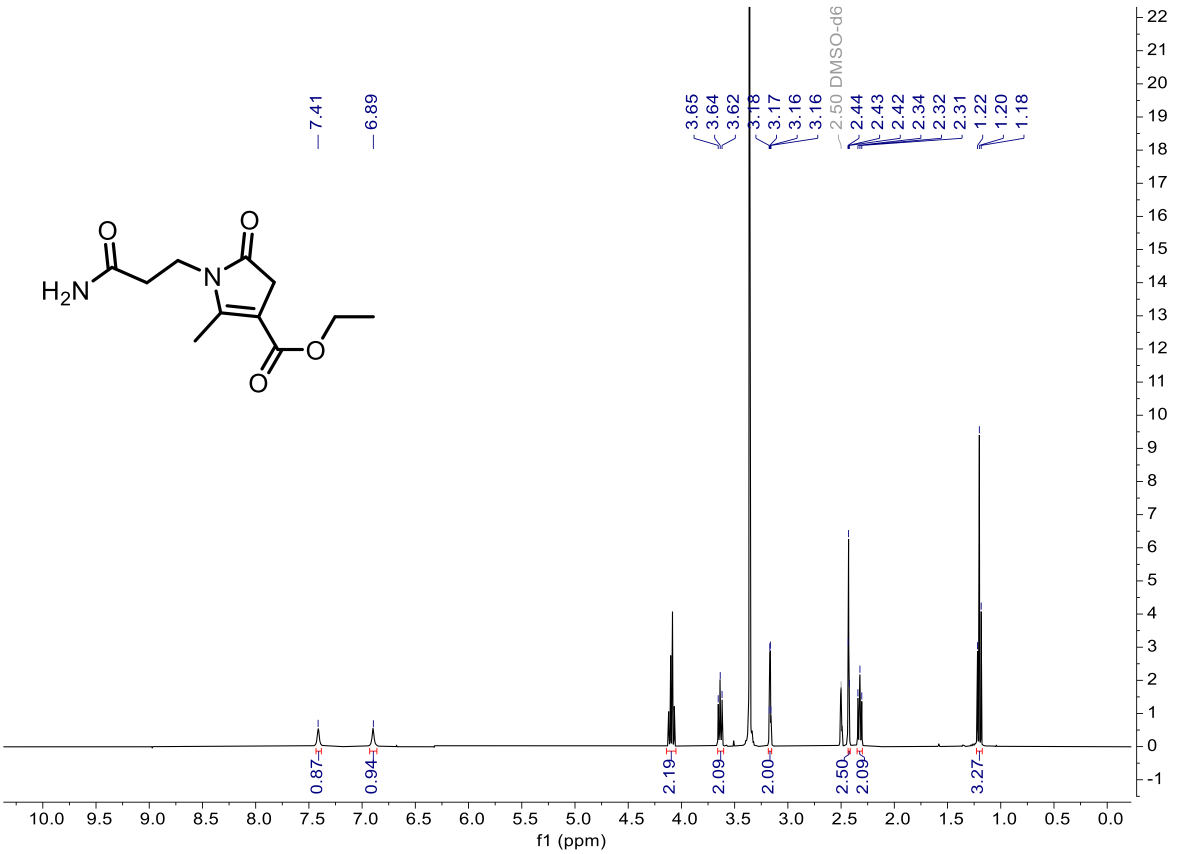

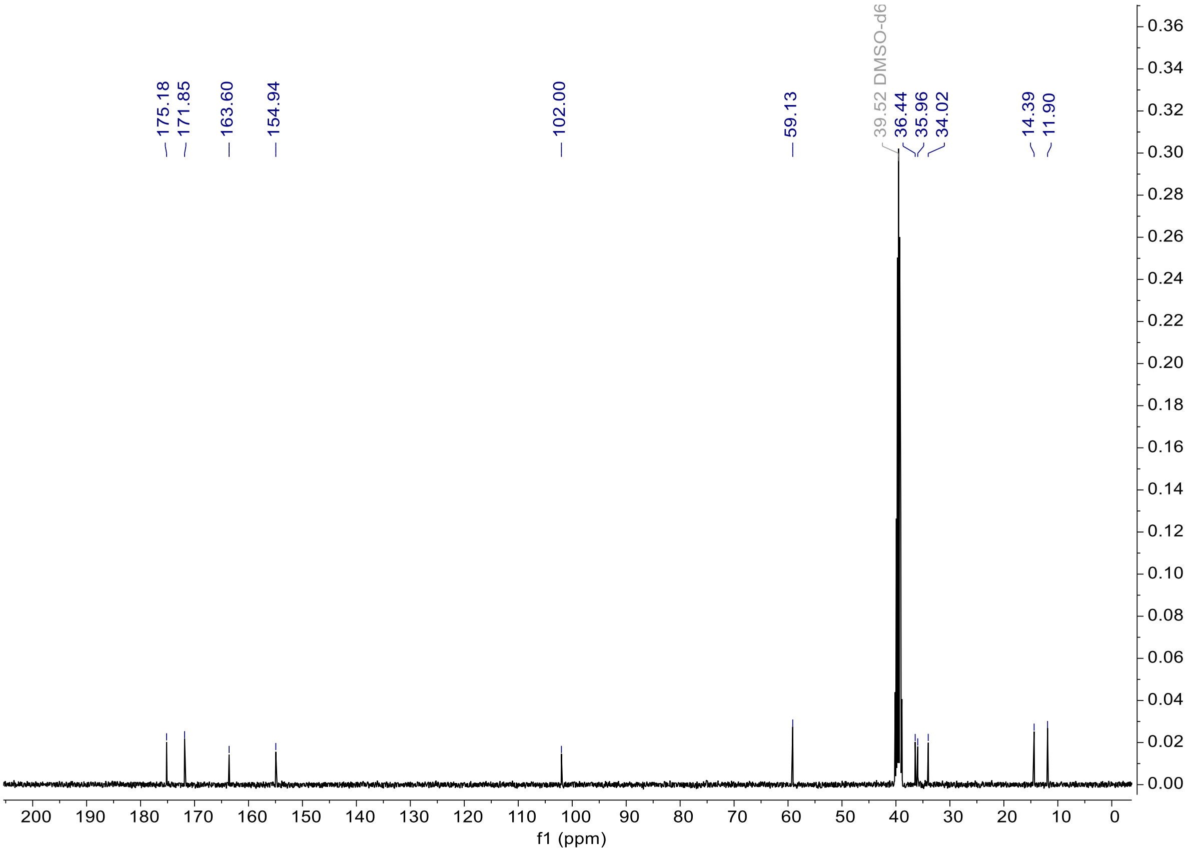

NMR spectra for **7zb**

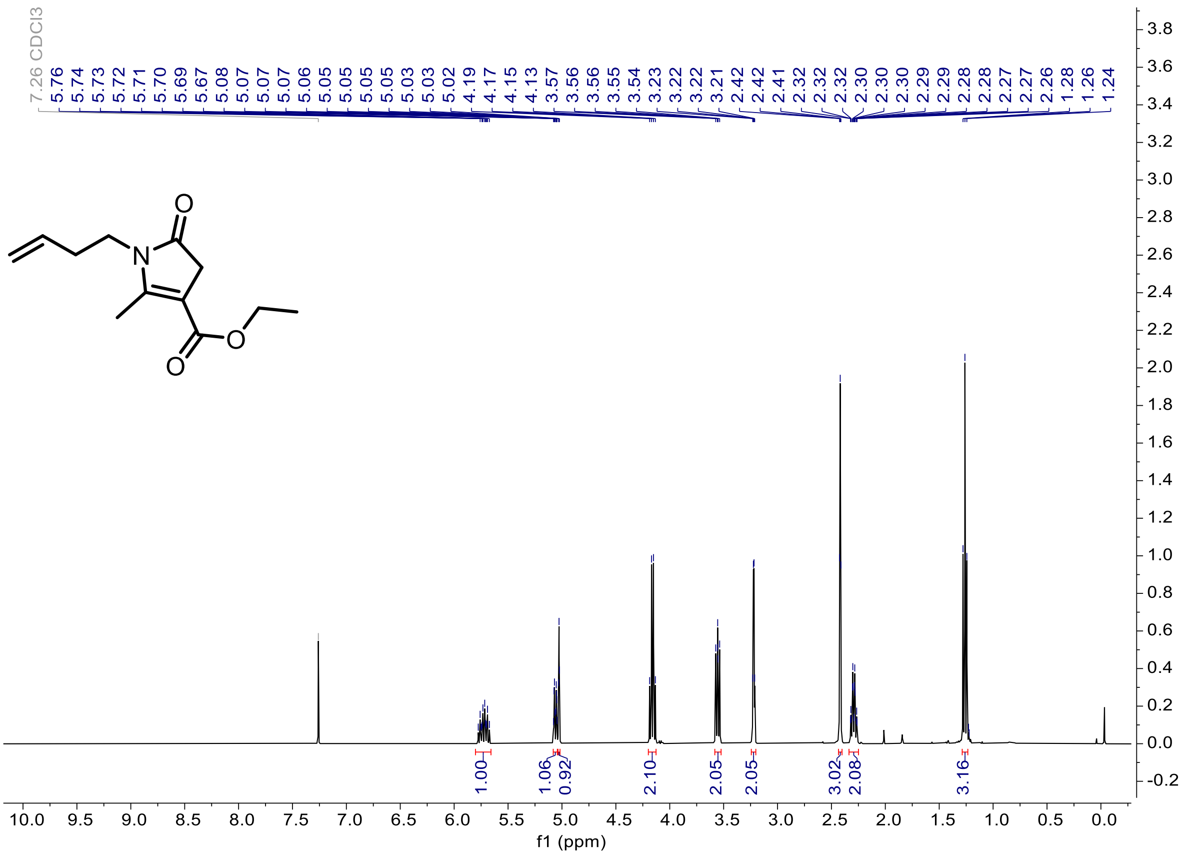

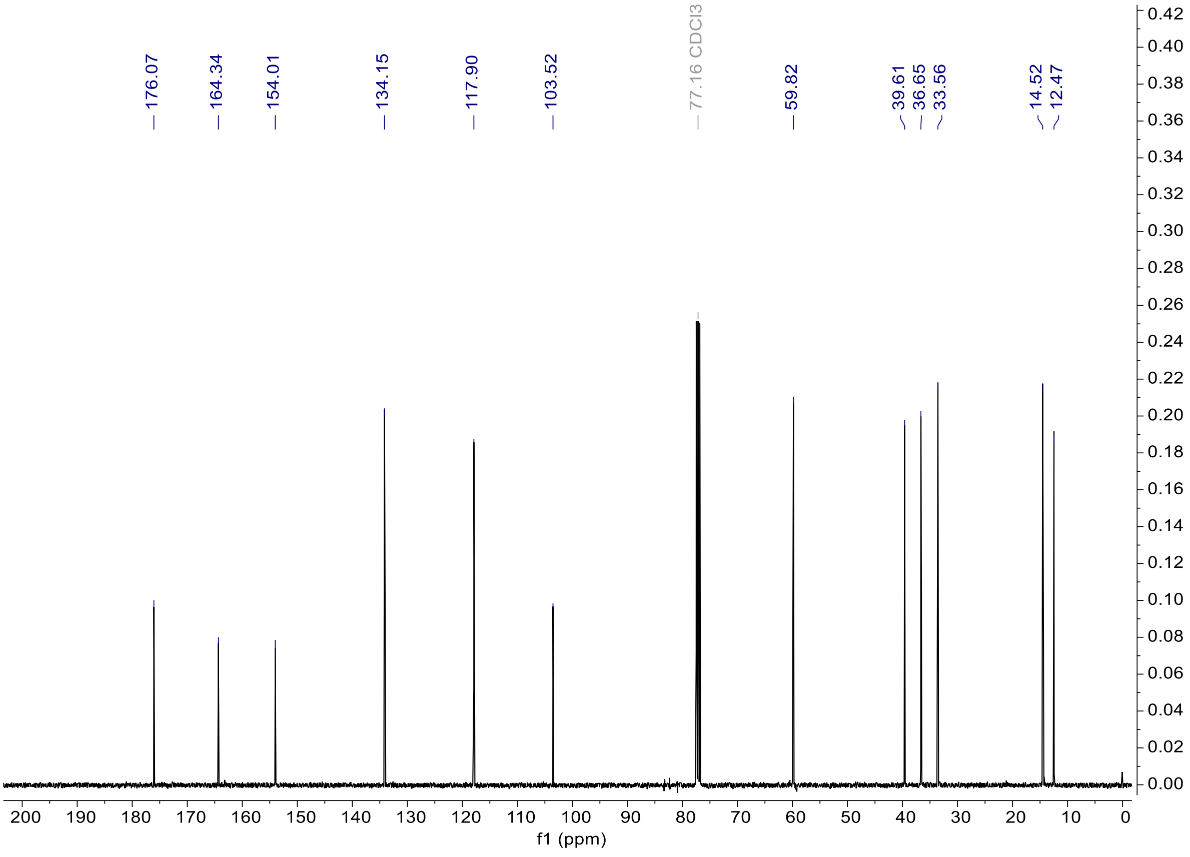

NMR spectra for **7zc**

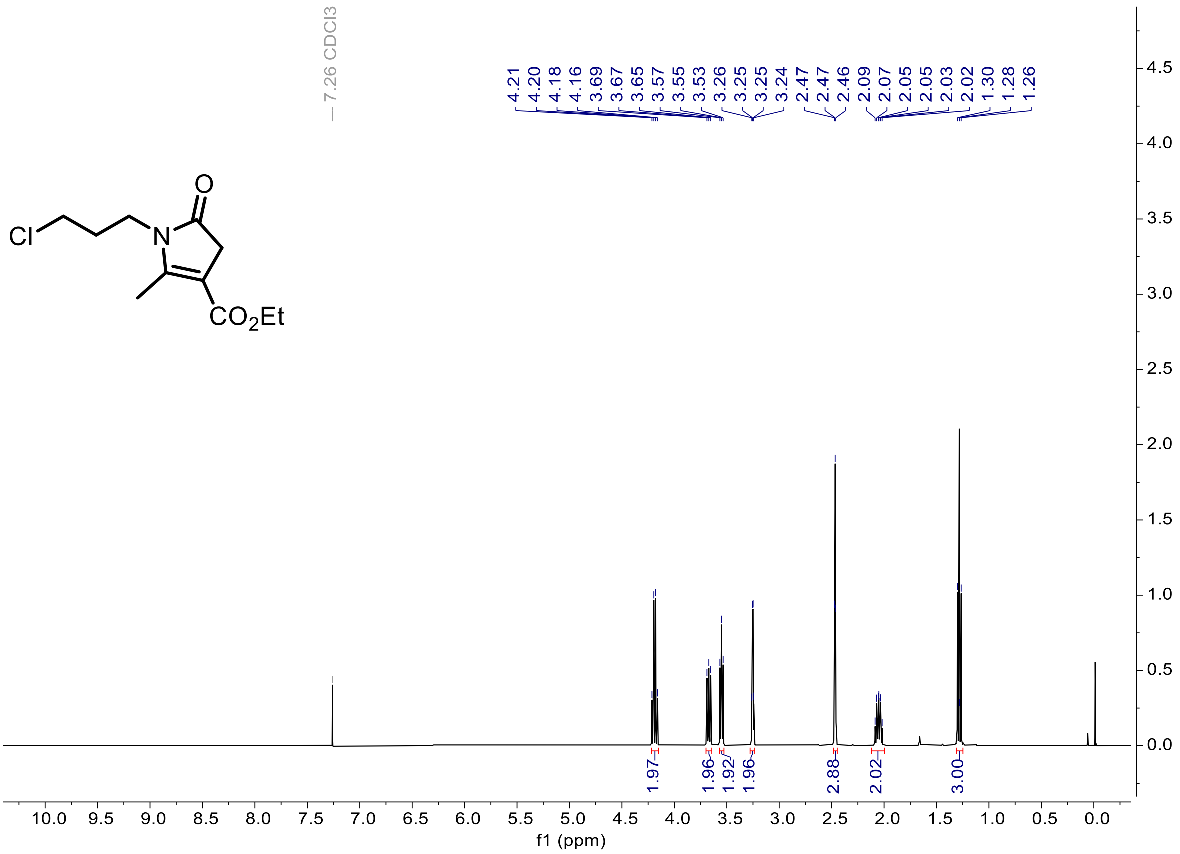

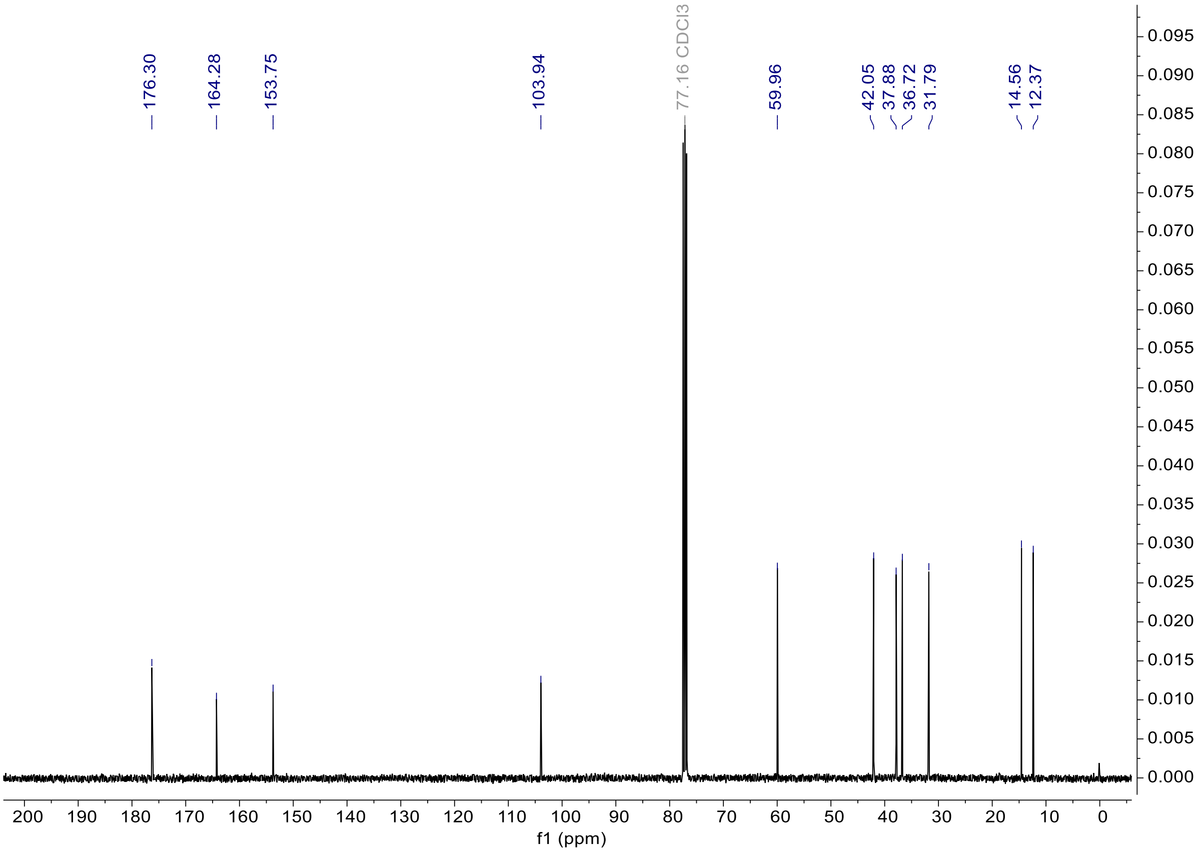

NMR spectra for **7zd**

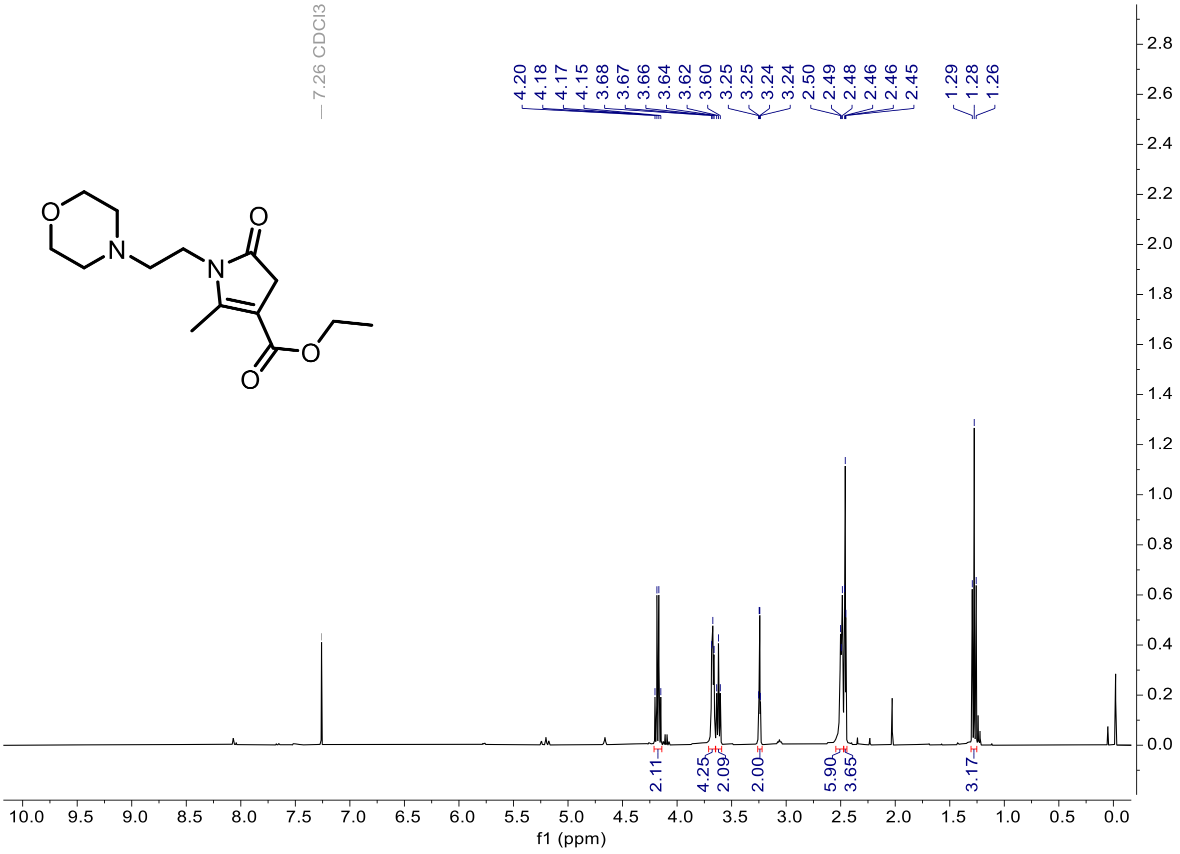

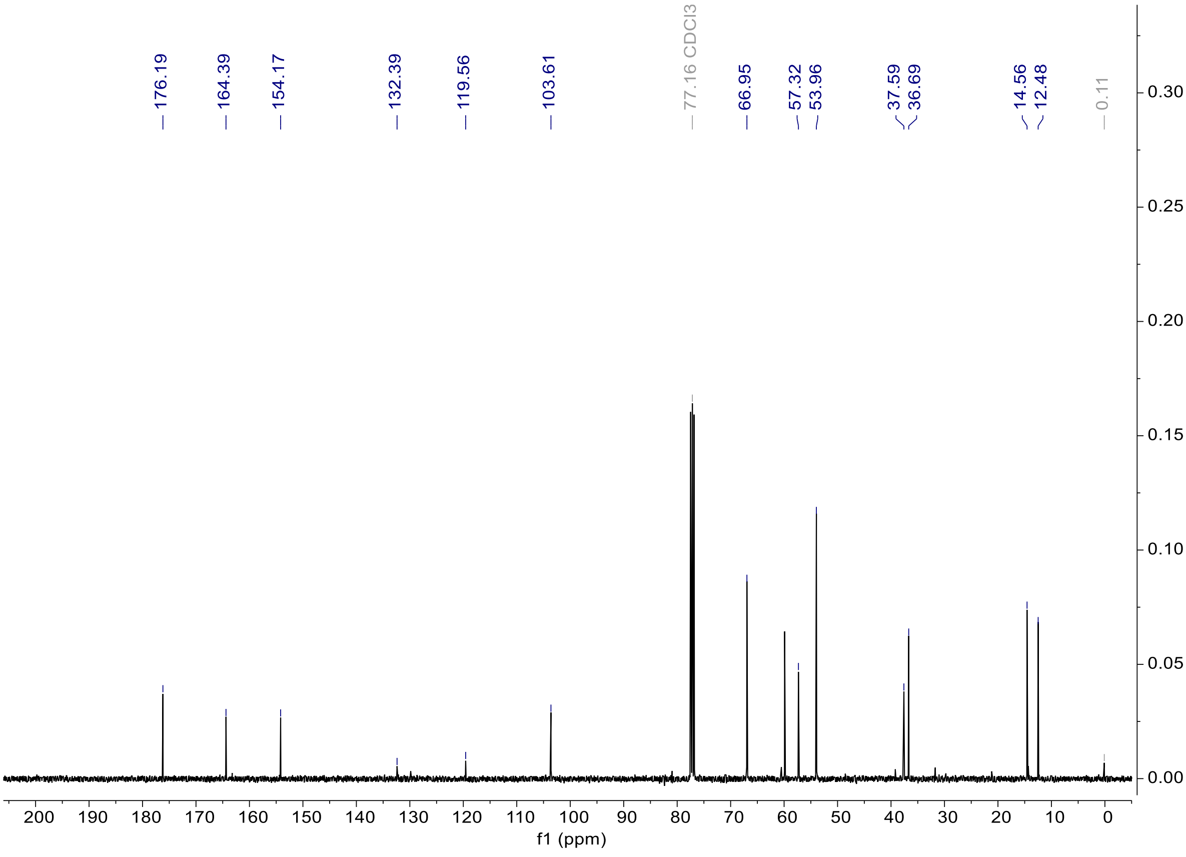

NMR spectra for **7ze**

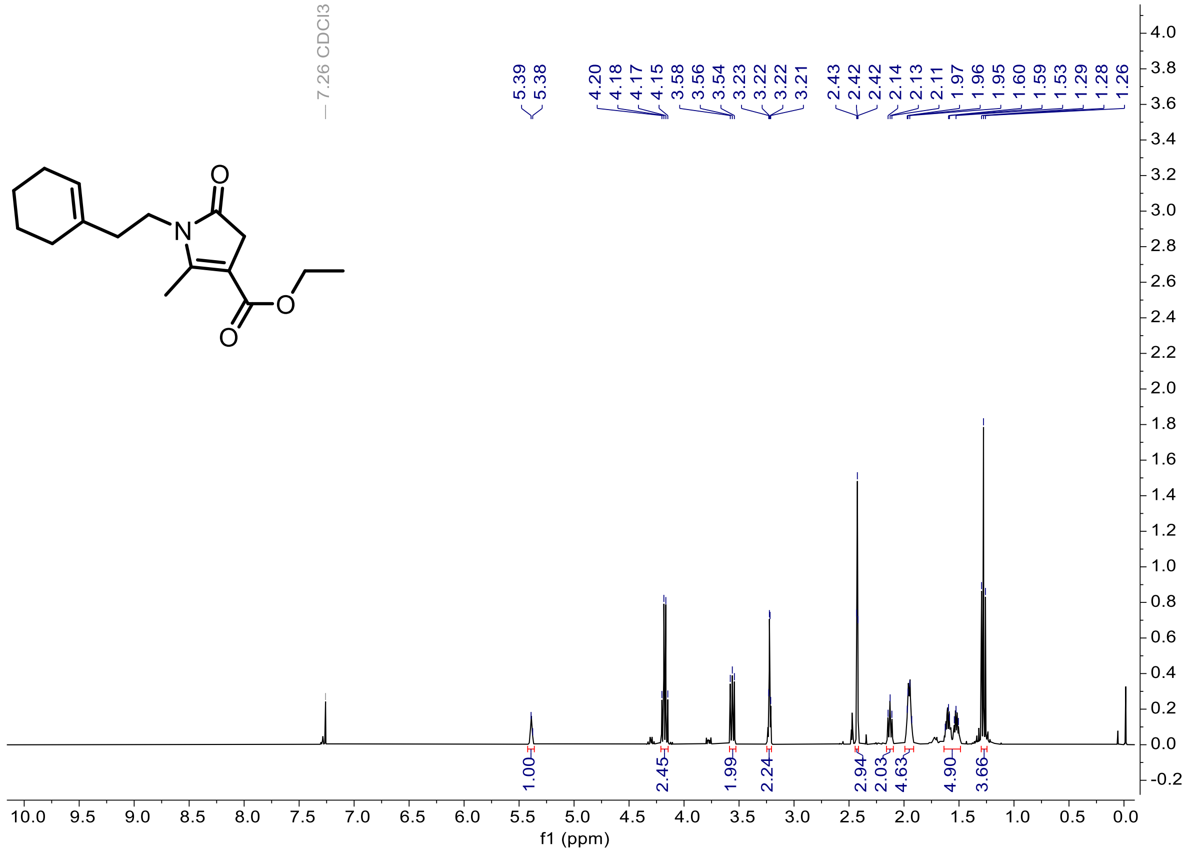

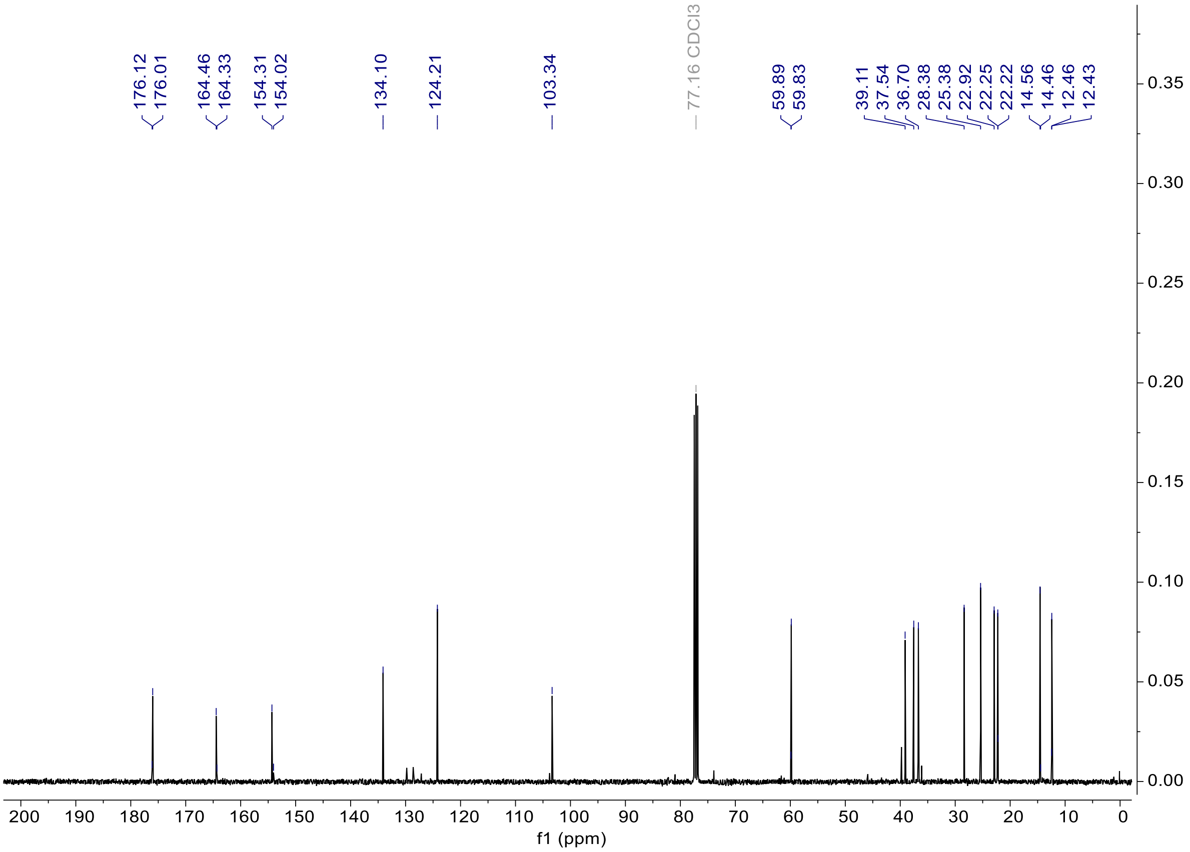

NMR spectra for **7zf**

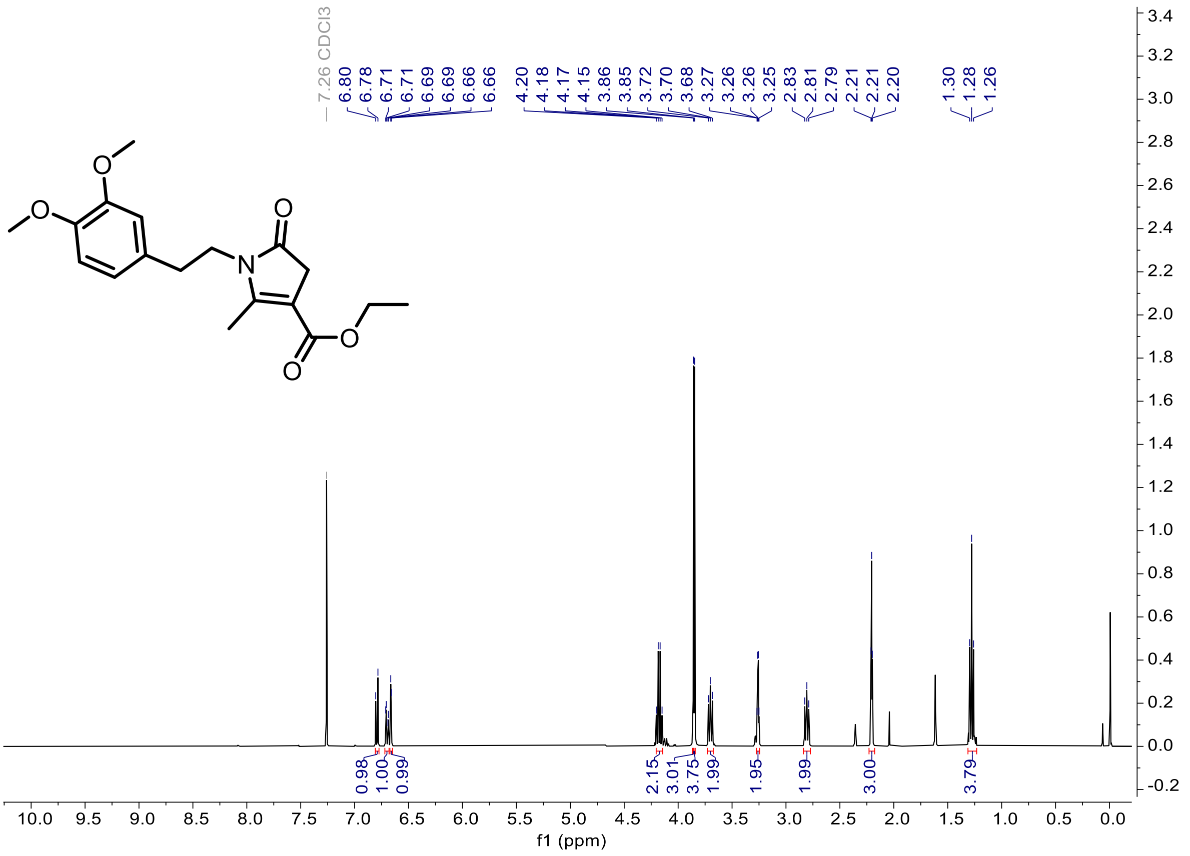

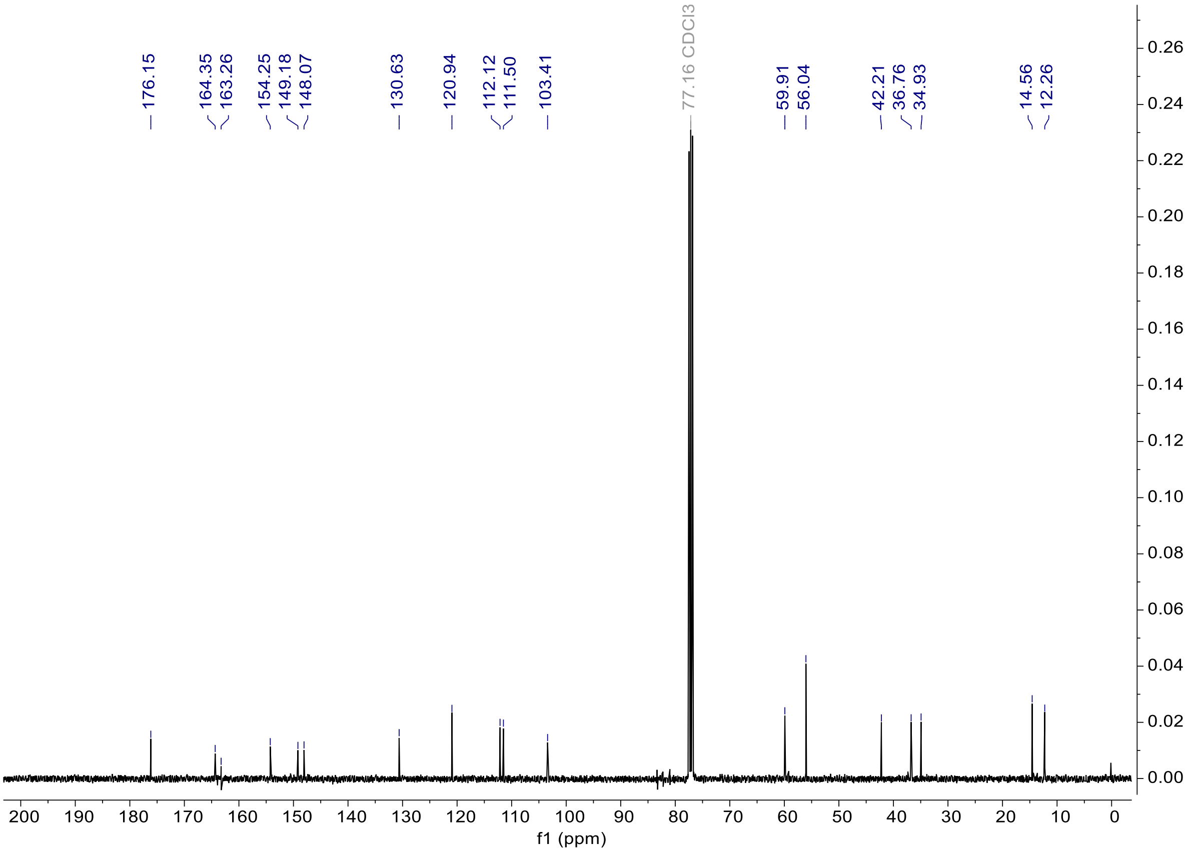

NMR spectra for **7zg**

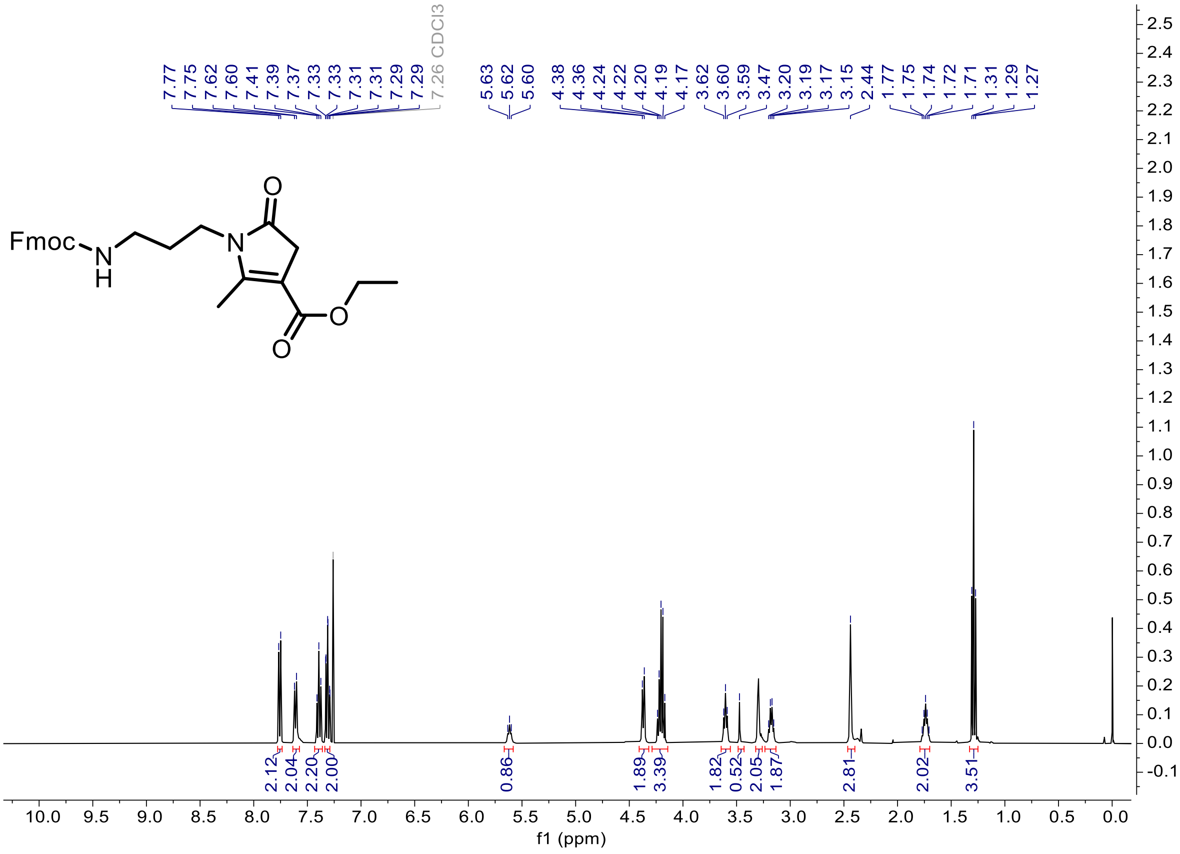

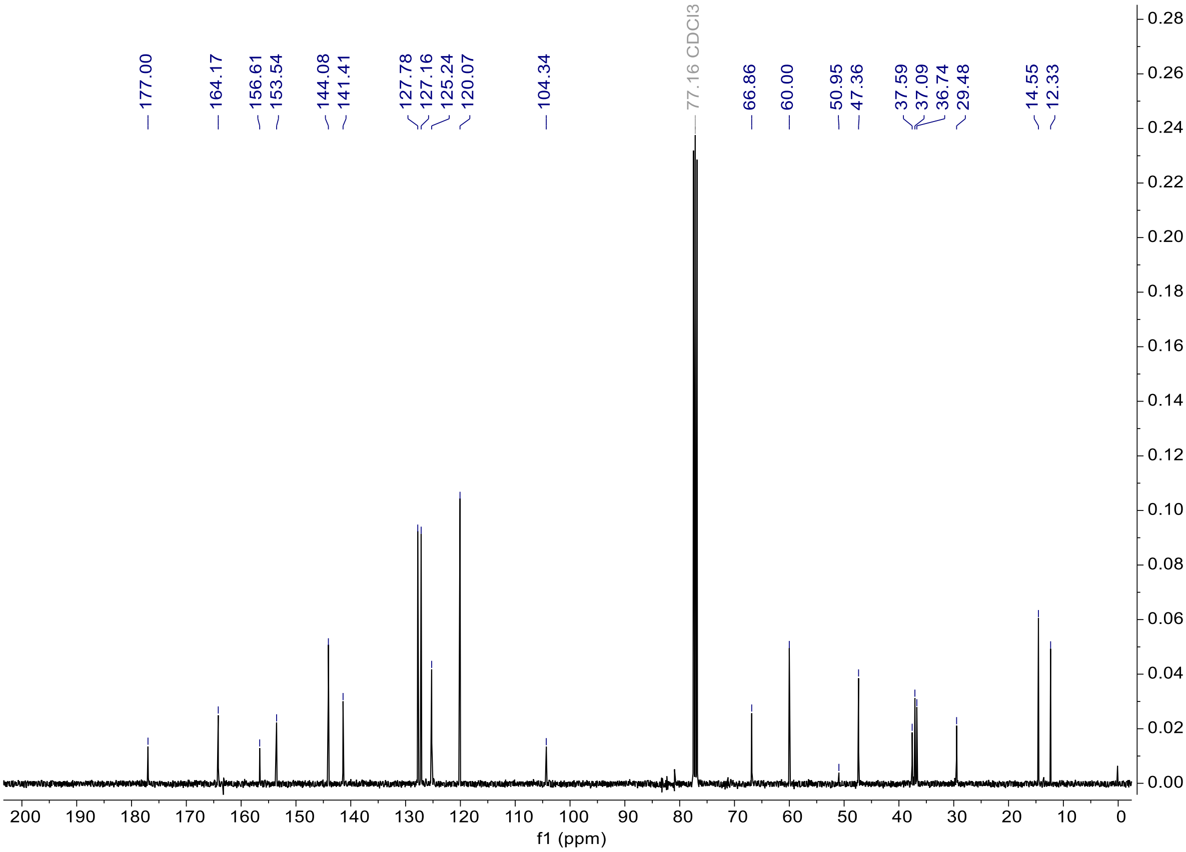

NMR spectra for **7zh**

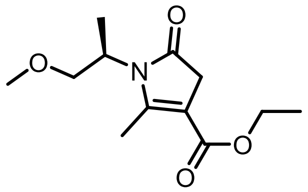

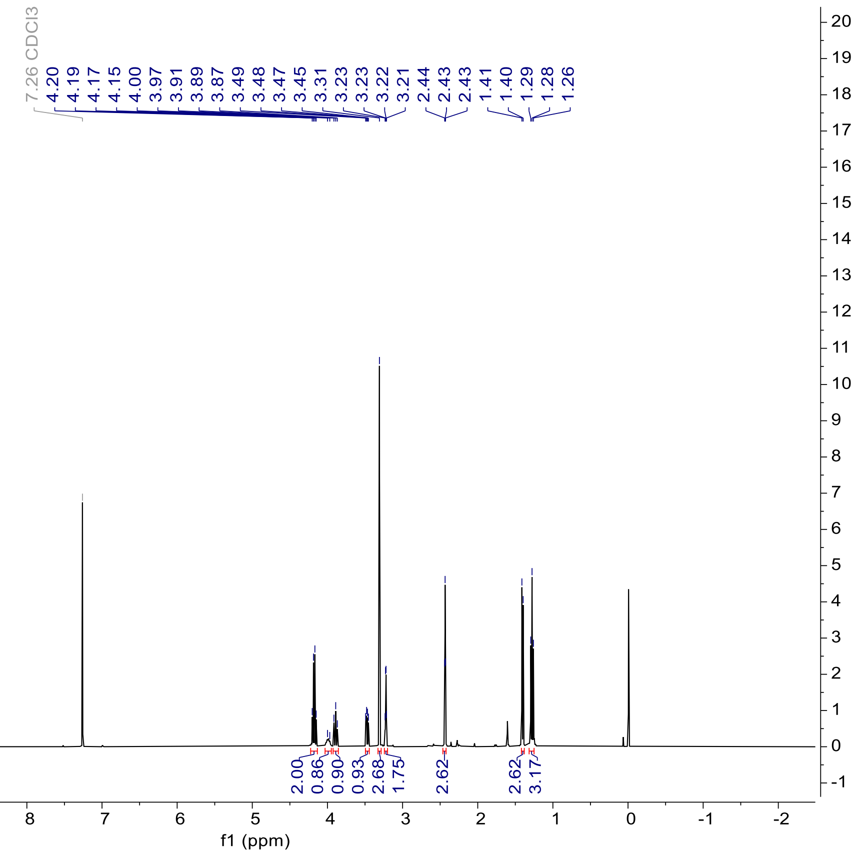

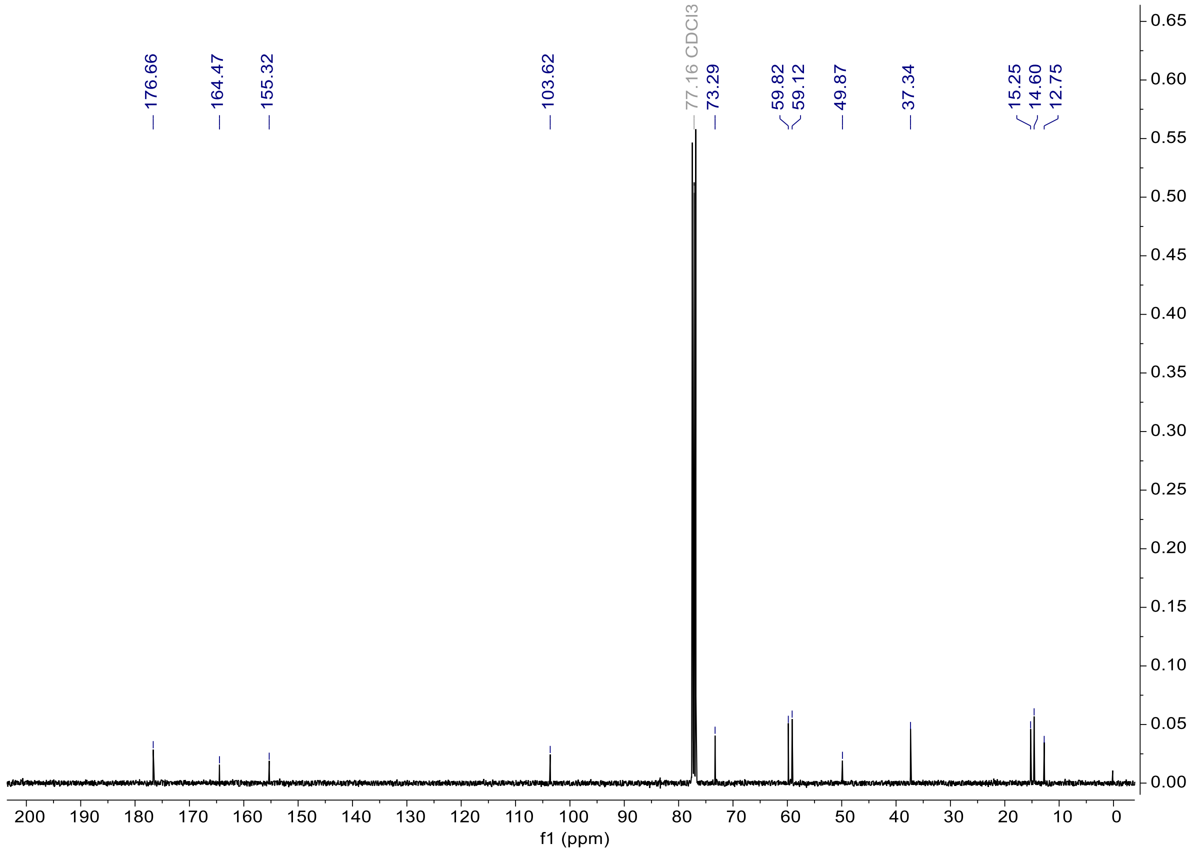

NMR spectra for **7zi**

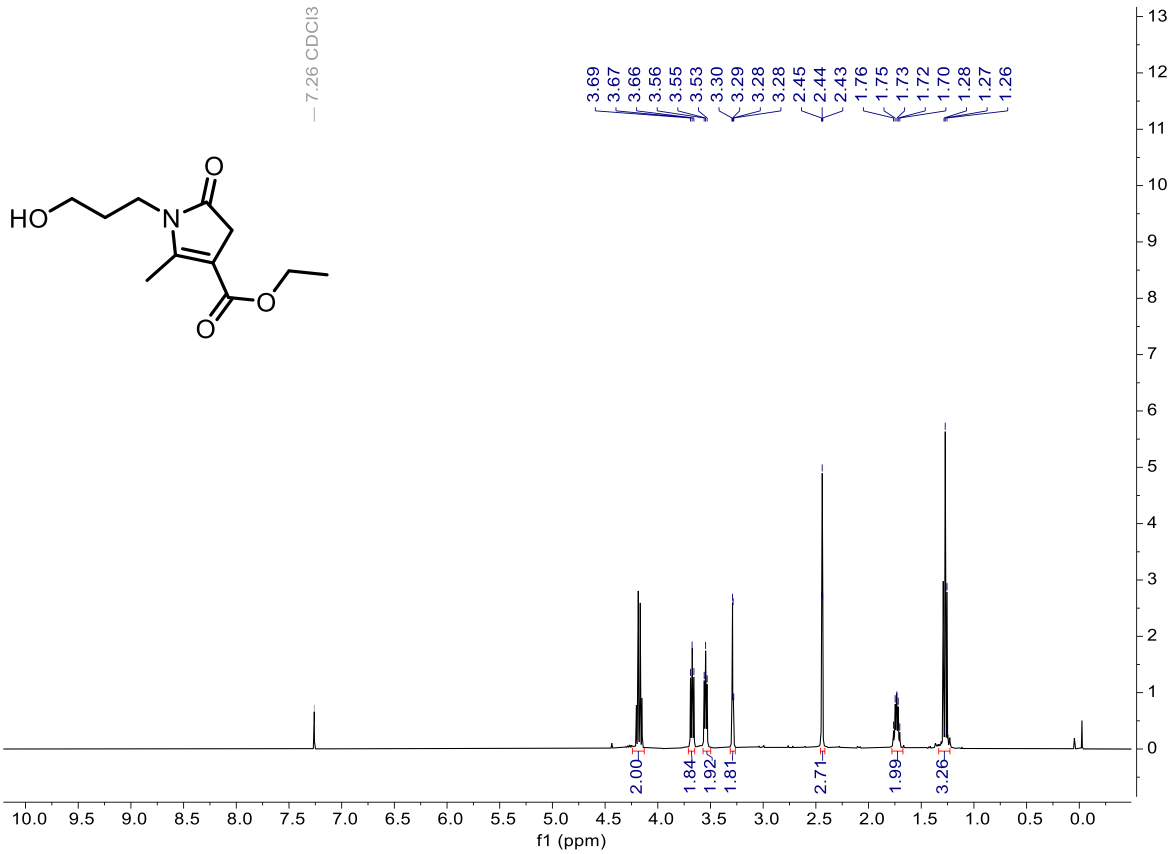

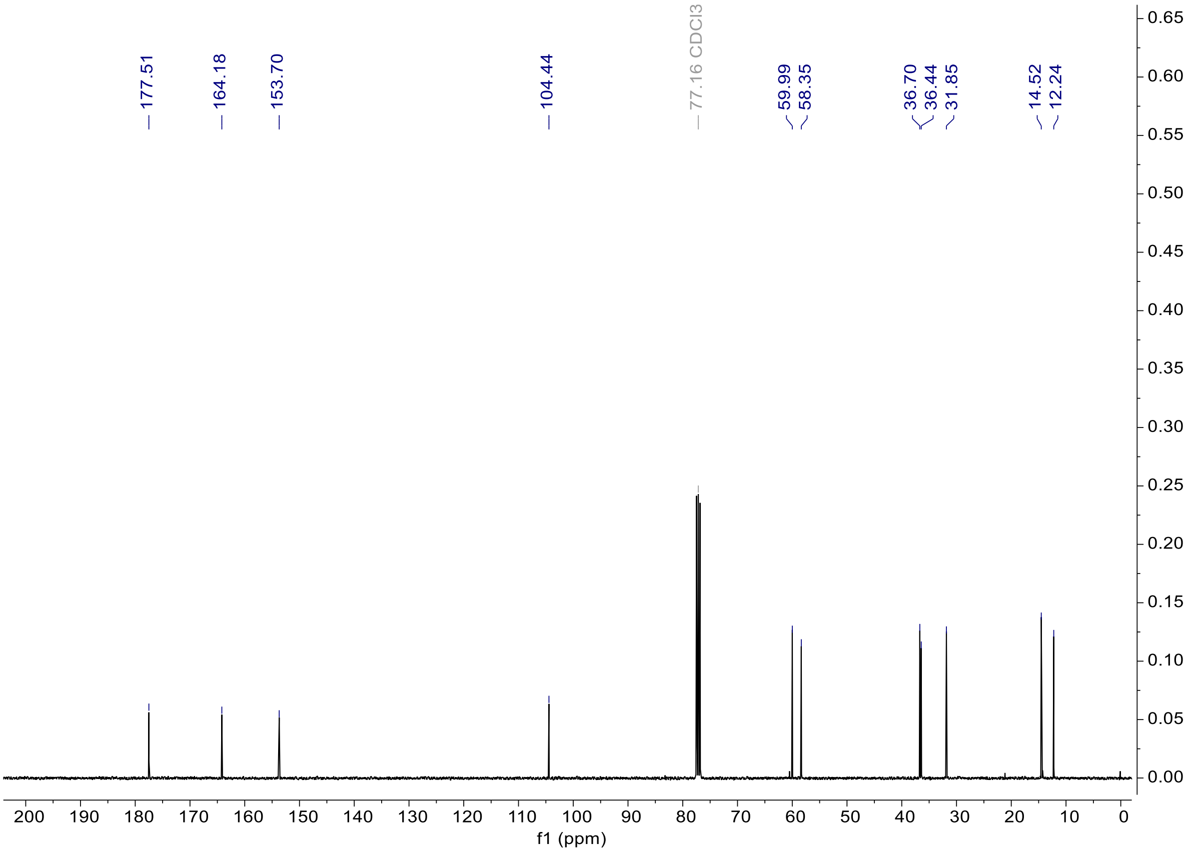

5) References

[1] Robinson, W. X. Q.; Mielke, T.; Melling, B.; Cuetos, A.; Parkin, A.; Unsworth, W. P.; Cartwright, J.; Grogan, G. Comparing the Catalytic and Structural Characteristics of a 'Short' Unspecific Peroxygenase (UPO) Expressed in Pichia pastoris and Escherichia coli. *ChemBioChem*, **2023**, *24*, e202200558

[2] Akhmedov, Sh. T.; Sadykhov, N. S.; Ismailov, V. M.; Akhundova, M. A.; Sadovaya, N. K.; Karimov, F. N.; Zefirov, N. S. *Chem. Heterocycl. Compd.* **1986**, *22*, 1291–1295

[3] Oyejobi, A.; Gao, D.; Wang, J.; Tang, X.; Wang, L. The Green Synthesis of 2-Pyrrolin-5-ones from Biorenewable Glyoxal, Diketones and Amines. *Eur. J. Org. Chem*. **2024**, *27*, e202400063.

[4] Ronaghi, N.; Fialho, D. M.; Jones, C. W.; France, S.; Conversion of Unprotected Aldose Sugars to Polyhydroxyalkyl and *C*-Glycosyl Furans via Zirconium Catalysis. *J. Org. Chem.* **2020**, *85*, 15337–15346

[5] Cores, A.; Estévez, V.; Villacampa, M.; Menéndez, J. C. Three-component access to 2-pyrrolin-5-ones and their use in target-oriented and diversity-oriented synthesis *RSC Adv.* **2016**, *6*, 39433

[6] Arcadi, A.; Attanasi, O. A.; Liao, Z.; Serra-Zane, F. The Reaction of Meldrum's Acid with Conjugated Azoalkenes: A Convenient Route to 1-Amino-1*H*-pyrrol-2(3*H*)-ones. *Synthesis* **1994**, 605–608

[7] Danahay, H.; Gosling, M.; Fox, R.; Lilley, S.; Charlton, H.; Hargrave, J.D.; Schofield, T.B.; Hay, D.A.; Went, N.; McMahon, P.; Marlin, F.; Scott, J.; Vile, J.; Hewison, S.; Ellam, S.; Brown, S.; Sabater, J.; Kennet, G.; Lightowler, S.; Collingwood, S.P. Optimisation of a novel series of ENaC inhibitors, leading to the selection of the long-acting inhaled clinical candidate ETD001, a potential new treatment for cystic fibrosis, *Eur. J. Med. Chem.* **2025**, 282, 117040
